# HNF4A-BAP31-VDAC1 axis synchronously regulates cell proliferation and ferroptosis in gastric cancer

**DOI:** 10.1038/s41419-023-05868-z

**Published:** 2023-06-09

**Authors:** Qingqing Zhou, Tengfei Liu, Wenjing Qian, Jun Ji, Qu Cai, Yangbing Jin, Jinling Jiang, Jun Zhang

**Affiliations:** 1 Department of Oncology, Ruijin Hospital, Shanghai Jiao Tong University School of Medicine, Shanghai, 200025 China; 2 Department of Oncology, Ren ji Hospital, Shanghai Jiao Tong University School of Medicine, Shanghai, 200127 China; 3 Operating Room, Ruijin Hospital, Shanghai Jiao Tong University School of Medicine, Shanghai, 200025 China; 4 Shanghai Institute of Digestive Surgery, Ruijin Hospital, Shanghai Jiao Tong University School of Medicine, Shanghai, 200025 China

**Keywords:** Gastric cancer, Translational research

## Abstract

B cell receptor associated protein 31 (BAP31) is closely associated with tumor progression, while the role and mechanism of BAP31 in gastric cancer (GC) remains unknown. This study explored that BAP31 was upregulated in GC tissues and high expression indicated poor survival of GC patients. BAP31 knockdown inhibited cell growth and induced G1/S arrest. Moreover, BAP31 attenuation increased the lipid peroxidation level of the membrane and facilitated cellular ferroptosis. Mechanistically, BAP31 regulated cell proliferation and ferroptosis by directly binding to VDAC1 and affected VDAC1 oligomerization and polyubiquitination. HNF4A was bound to BAP31 at the promoter and increased its transcription. Furthermore, knockdown of BAP31 inclined to make GC cells vulnerable to 5-FU and ferroptosis inducer, erastin, in vivo and in vitro. Our work suggests that BAP31 may serve as prognostic factor for gastric cancer and act as potential therapeutic strategy for gastric cancer.

## Introduction

Globally, gastric cancer (GC) severely affects human health and living quality, with high incidence rate and mortality rate [1, 2]. Despite the decreasing incidence of GC, recent statistical data indicate an increasing occurance in young population, which may change the age spectrum of GC patients [3]. Since GC is a heterogeneous disease both molecularly and phenotypically [4], an enormous potential exists for its treatment by using target therapy in combination with sequential lines of chemotherapy.

The B Cell Receptor Associated Protein 31 (BAP31) gene is located on X chromosome at q28, encoding a ubiquitously expressed membrane protein in endoplasmic reticulum (ER) [5]. As a chaperone protein in ER, BAP31 plays an important role in diverse biological processes such as the transport of membrane proteins from ER, and mediates crosstalk with mitochondria in caspase 8-mediated apoptosis, mitochondria dysfunction, etc [6–9]. BAP31 is upregulated in lung cancer, breast cancer, liver cancer, kidney cancer and many other kinds of cancer [10]. Furthermore, BAP31 may serve as a potential biomarker for prognostic prediction in non-small cell lung carcinoma and hepatocellular carcinoma [11, 12]. Also, it has been found that BAP31 exerts a significant influence on tumor proliferation in cervical cancer [13]. Similar phenotypes appeared at human embryonic stem cells, in which BAP31 attenuation induced cell apoptosis and inhibited cell proliferation [14]. BAP31 physically associated with epithelial cell adhesion molecule (EpCAM), and EpCAM expression significantly decreased with BAP31 knockdown, while BAP31 overexpression increased its level and enhanced cell adhesion [15]. Despite these progresses made in understanding the role played by BAP31 in cancers, research pertaining to unravel the role of BAP31 in GC is scarce.

As a novel form of regulated cell death, ferroptosis is characterized by iron overload, lipid reactive oxygen species (ROS) accumulation, and lipid peroxidation [16]. Increasing evidences have shown that ferroptosis extensively participates in physiological processes and various diseases, especially in cancer initiation, progression, and suppression [17, 18]. Examining the potentials of ferroptosis in cancer therapy is a fast-growing field of study, where several ferroptosis inducers have been found to be beneficial and promising in pre-clinical studies [19]. Currently, fluoropyrimidine with platinum-based chemotherapy serve as the standard regimen in advanced GC, in which drug resistance occurs frequently [20]. Cancer cells exhibit an increased iron demand compared with normal cells, and the iron dependency can make cancer cells more vulnerable to ferroptosis and creates the potential of ferroptosis to be a new promising way to kill therapy-resistant cancers [21]. Intriguingly, increasing evidence has emerged that ferroptosis inducer may help sensitize chemotherapy in gastric cancer. Zhang et al. demonstrated that CAFs secreted exosomal miR-522 to inhibit ferroptosis in cancer cells by targeting ALOX15 and blocking lipid-ROS accumulation, and blocking this axis enhanced sensitivity of chemotherapy [22]. There were studies demonstrating that inhibition of STAT3-ferroptosis regulatory axis alleviated chemoresistance and ferroptosis activity affected oxaliplatin resistance [23, 24]. Since ferroptosis exerts synergistic effects in combination with currently approved treatment [25], which gives us a hint for its potential application in clinic.

Here, this study demonstrates that BAP31 expression increased in GC and closely related with worse prognosis. BAP31 overexpression accelerated GC cell growth and inhibited ferroptosis, whereas BAP31 knockdown suppressed cell growth and promoted ferroptosis. Mechanistically, BAP31 was transcriptionally activated by HNF4A, and further inhibited ferroptosis and facilitated cell proliferation through binding with voltage-dependent anion channel 1 (VDAC1) by promoting its degradation through the ubiquitin-proteasome system and affecting VDAC1 oligomerization. Overall, we depicted the mechanism of BAP31 in the development of GC and tested the feasibility of treating GC with ferroptosis inducer in combination with chemotherapy.

## Results

### Increased BAP31 expression predicts worse prognosis in GC patients

**Fig. 1:**
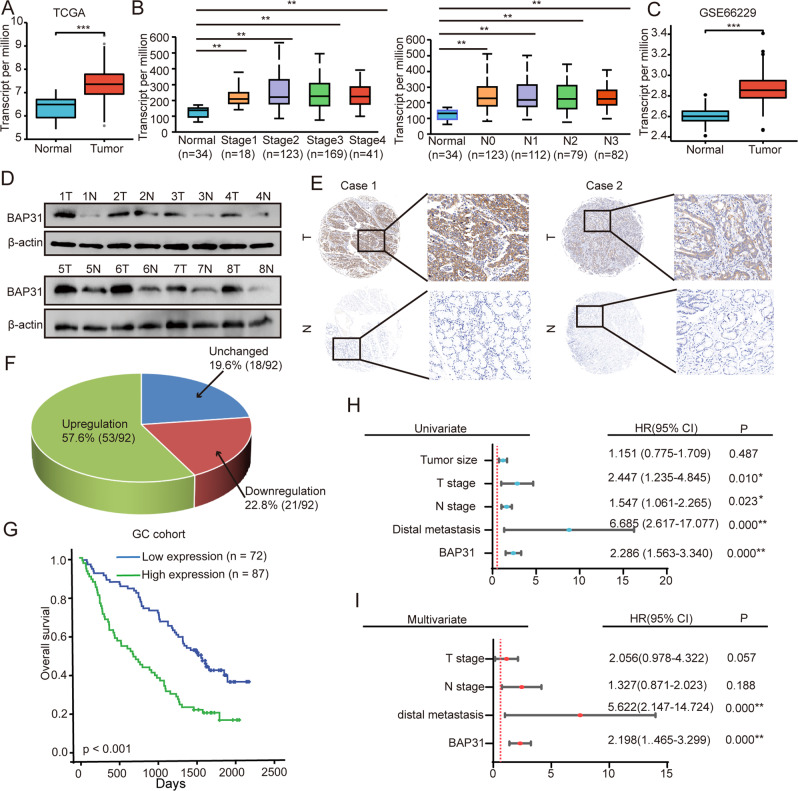
GC with increased BAP31 expression indicate a worse prognosis. **A** BAP31 was analyzed in GC tissues and in non-cancerous gastric tissues derived from TCGA data. **B** TCGA datasets were used to analyze the expression of BAP31 in different GC stages, and nodal metastasis. **C** BAP31 expression in GC tissues as well as noncancerous gastric tissues was analyzed using the GSE66229 dataset. **D** An analysis of BAP31 expression in GC tissues compared to noncancerous gastric tissues by western blot. **E** Representative IHC images showing the level of BAP31 in GC tissues and the corresponding noncancerous tissues. **F** Pie chart illustrating the proportion of upregulation, unchanged and downregulation in BAP31 for comparison between GC tissues and noncancerous tissues. **G** Kaplan–Meier analysis of overall survival in GC patients with differential BAP31 expression in 159 samples. Univariate (**H**) and multivariate (**I**) Cox proportional hazard analyses were conducted to evaluate the HR of BAP31 in GC. *p < 0.05, **p < 0.01.

It has been reported that BAP31 is increased in multiple cancers [10], including colon adenocarcinoma (COAD), liver hepatocellular carcinoma (LIHC), brain lower grade glioma (LGG), etc., which is corroborated by data of different cohorts from TCGA (The Cancer Genome Atlas) database (Supplementary Fig. S1A). To explore the biological behavior of BAP31 in GC, the expression of BAP31 in GC was analyzed using TCGA. The expression of BAP31 was higher in GC tissues compared with noncancerous tissues (Fig. 1A). Furthermore, the mRNA level of BAP31 positively related to tumor malignancies (Fig. 1B). In addition, the expression of BAP31 was increased in GC tissues compared with noncancerous tissues using GSE66229 datasets (Fig. 1C). Western blots were conducted in GC samples from our laboratory, showing that BAP31 expression in GC tissues was higher than that in noncancerous tissues (Fig. 1D). Besides, immunohistochemistry (IHC) assay implied a higher BAP31 protein expression in GC tumor tissues at a ratio of 57.6% (53/92) (Fig. 1E, F). Overall, above studies suggest that BAP31 expression is significantly enhanced in GC.

It is of note that BRCA, ESCA, HNSC and LGG patients with low BAP31 expression had longer overall survival (OS) than those with high expression (log-rank, *p* < 0.05), as determined in Kaplan–Meier analysis of the TCGA GC cohort (Supplementary Fig. S1B). Considering the increased BAP31 expression in GC, we next investigated the clinical significance of BAP31 in GC patients. We observed BAP31 level in 159 GC tissues with IHC assay, and patients were separated into low and high level group according to IHC score (Fig. 1E). The results suggested that BAP31 expression was positively associated with tumor size, T stage and Ki-67 level (Table 1). Kaplan–Meier survival analysis revealed that higher level of BAP31 indicated a shorter OS time (*p* < 0.001, Fig. 1G). Furthermore, univariate and multivariate COX proportional hazard analyses implied that BAP31 might serve as an independent factor in GC patients (Fig. 1H–I). In conclusion, these findings suggest BAP31 to be a valuable predictive factor in GC patients.

**Table 1:** Correlation between BAP31 levels in GC patients and their clinicopathological characteristics.

Clinicopathological features	Number	Low expression *N* (%)	High expression *N* (%)	*p* value
Age				
<60	45	28 (32.2)	17 (23.6)	0.232
≥60	114	59 (67.8)	55 (76.4)	
Gender				
Male	55	55 (63.2)	49 (68.1)	0.523
Female	104	32 (36.8)	23 (31.9)	
Tumor size				
≤5 cm	94	65 (74.7)	39 (54.2)	0.007**
>5 cm	55	22 (25.3)	33 (45.8)	
T stage				
I-II	20	16 (18.4)	4 (5.6)	0.015*
III-IV	139	71 (81.6)	68 (94.4)	
N stage				
0-I	78	44 (50.6)	34 (47.2)	0.674
II-III	81	43 (49.4)	38 (52.8)	
Distal metastasis				
Negative	152	83 (97.6)	69 (95.8)	0.502
Positive	5	2 (2.4)	3 (4.2)	
Nerve invasion				
Negative	123	68 (78.2)	55 (76.4)	0.790
Positive	36	19 (21.8)	17 (23.6)	
Vascular invasion				
Negative	117	64 (73.6)	53 (73.6)	0.995
Positive	42	23 (26.4)	19 (26.4)	
Ki67				
Negative	35	25 (28.7)	10 (14.1)	0.027*
Positive	123	62 (71.3)	61 (85.9)	
P53				
Negative	37	22 (25.3)	15 (22.1)	0.640
Positive	118	65 (74.7)	53 (77.9)	
HER-2				
Negative	83	43 (50.0)	40 (57.1)	0.374
Positive	73	43 (50.0)	30 (42.9)	
CEA				
Negative	15	11 (23.9)	4 (16.0)	0.435
Positive	56	35 (76.1)	21 (84.0)	
EGFR				
Negative	15	10 (27.8)	5 (15.2)	0.204
Positive	53	26 (72.2)	28 (84.8)	

*p < 0.05; **p < 0.01.

### BAP31 overexpression facilitates GC cell growth and promotes G1/S transition

**Fig. 2:**
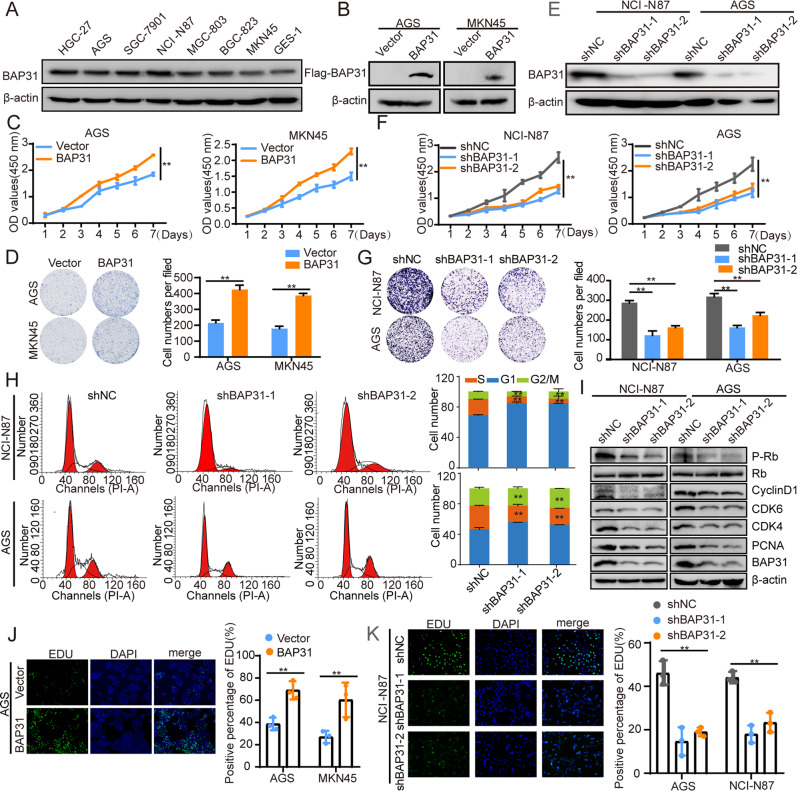
BAP31 promotes GC cell growth. **A** Western blot was applied to determine the endogenous level of BAP31 in GC cell lines. **B** Western blot was applied to detect the expression of BAP31 in GC cells with BAP31 overexpression. **C** CCK8 assay was utilized to evaluate the influence of BAP31 overexpression on GC cell growth. **D** Colony formation assay was used to evaluate the influence of BAP31 overexpression on GC cell growth. **E** Western blot was applied to detect the expression of BAP31 in GC cells with BAP31 knockdown. **F** CCK8 assay was utilized to evaluate the influence of BAP31 knockdown on GC cell growth. **G** Colony formation assay was used to evaluate the influence of BAP31 knockdown on GC cell growth. **H** Cell cycle distributions in BAP31 knockdown GC cells were detected by flow cytometry. **I** Western blot was applied to detect the cell cycle-related proteins with BAP31 knockdown GC cells. EdU assay was used to assess growth on GC cells with BAP31 overexpression (**J**) or BAP31 knockdown (**K**). *p < 0.05, **p < 0.01.

To investigate the role of BAP31 in GC progression, GC cells were chosen for loss- or gain-of-function studies based on their low or high endogenous BAP31 expression, respectively (Supplementary Fig. S2A, Fig. 2A). The results demonstrated that BAP31 overexpression promoted GC cell growth and colony formation (Fig. 2B–D). Conversely, BAP31 knockdown inhibited GC cell growth and colony formation (Supplementary Fig. S2B, Fig. 2E–G). To further elucidate the underlying mechanism, flow cytometry was used to analyze cell cycle distribution of NCI-N87 and AGS cells, which indicated BAP31 attenuation led to G1/S arrest (Fig. 2H). Correspondingly, the expression of related factors such as cyclin-dependent kinase 4 (CDK4), cyclin-dependent kinase 6 (CDK6), cyclin D1, p-Rb (Ser807/811), and proliferating cell nuclear antigen (PCNA) were remarkably decreased in BAP31-knockdown group versus control group (Fig. 2I). DNA synthesis, as measured by EdU incorporation increased in BAP31-overexpressing and downregulated in BAP31-knockdown GC cells (Supplementary Fig. S2C, D and Fig. 2J, K). Moreover, the expression of BAP3 positively related with PCNA (R = 0.36, *p* < 0.01) and Ki-67 (R = 0.47, *p* < 0.01) in TCGA dataset (Supplementary Fig. S2E, F). Consequently, these studies illustrate that BAP31 facilitates cell growth via promoting cell cycle G1/S transition.

### BAP31 knockdown promotes ferroptosis and p38 MAPK pathway in GC cells

**Fig. 3:**
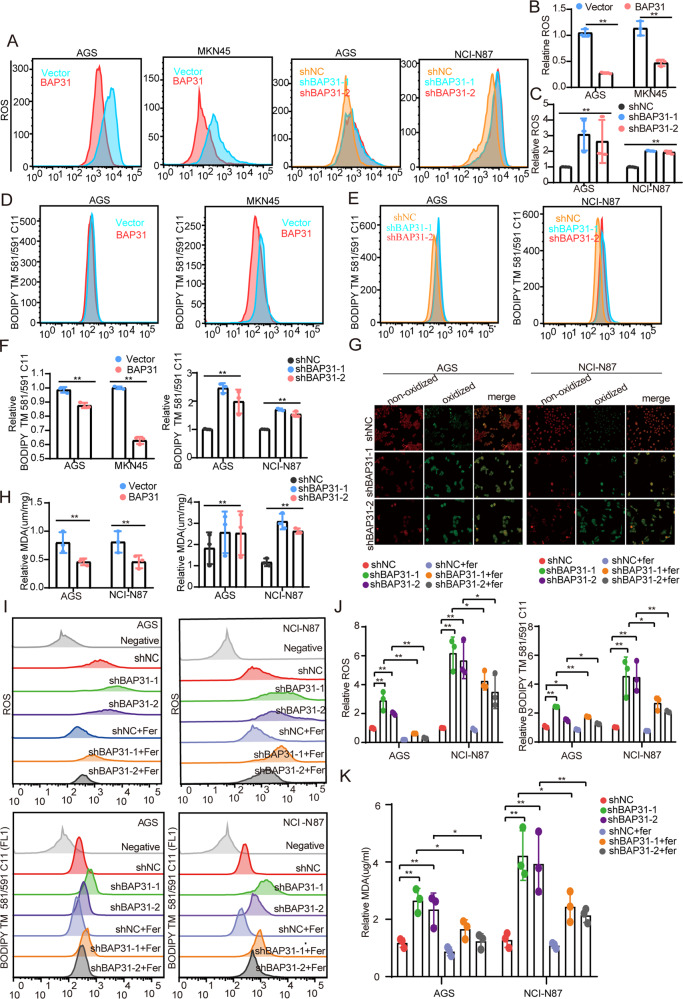
BAP31 attenuation facilitates lipid peroxidation and ferroptosis. **A**–**C** Flow cytometry was applied to detect ROS fluorescence on GC cells under BAP31 overexpression or knockdown, and cells were pre-treated with erastin (5 μM) for 12 h. **D**–**F** The GC cells with BAP31 overexpression or BAP31 knockdown pretreated with erastin (5 μM) for 12 h, and lipid ROS production was tested. **G** Confocal imaging detected lipid peroxidation under BAP31 knockdown in GC cells pretreated with erastin (5 μM) for 12 h. **H** BAP31-overexpressing or BAP31-knockdown GC cells pretreated with erastin (5 μM) for 12 h, and MDA content was assessed. **I**, **J** BAP31-knockdown GC cells pretreated with erastin (5 µM) for 12 h in the presence or absence of 2 μmol/L Fer-1, and then lipid ROS production was observed. **K** GC cells pretreated with erastin (5 µM) for 12 h in the presence or absence of 2 μmol/L Fer-1, and MDA content was assayed. *p < 0.05, **p < 0.01.

Ferroptosis is an iron-dependent form of necrotic cell death. Increased lipid peroxidation and enhanced ROS are characteristics of ferroptosis, making it different from other forms of cell death such as apoptosis, necrosis, and autophagy [26]. It has been reported that BAP31 – an integral ER membrane protein – is involved in ROS production through p22 ^phox^ and Keap1/Nrf2/HO-1 signaling pathway in microglia [27]. However, whether BAP31 plays a role in ferroptosis remains uncertain. ROS and lipid peroxidation accumulation are considered as classical hallmarks of ferroptosis [26]. The results demonstrated that BAP31 overexpression decreased the content of ROS and lipid peroxidation level, and its knockdown increased the content of ROS and lipid peroxidation level (Fig. 3A–F). Once lipid peroxidation occurs, the fluorescence color would switch from red to green, which was used in our study to identify the increase in lipid peroxidation level induced by BAP31 knockdown (Fig. 3G). This result was further confirmed by a decrease in the level of malon-dialdehyde (MDA) with BAP31 overexpression, and its elevated level upon BAP31 knockdown (Fig. 3H). As ferroptosis inhibitor, ferrostatin-1 could hinder ROS accumulation and lipid peroxidation induced by BAP31 knockdown (Fig. 3I, J). Furthermore, an elevated level of MDA upon BAP31 knockdown was counteracted by ferrostatin-1 (Fig. 3K). Recent studies suggested that lipid peroxide tended to activate p38 mitogen-activated protein kinase (MAPK) pathway [28]. Our results showed that BAP31 overexpression downregulated the level of P-p38, while BAP31 knockdown upregulated the level of P-p38 (Supplementary Fig. S3A), and immunofluorescence assay indicated that BAP31 knockdown was advantageous to nuclear translocation of p38 (Supplementary Fig. S3B). These findings imply that BAP31 knockdown enhances ferroptosis and activates p38 MAPK pathway in GC cells.

### Suppression of BAP31 sensitizes GC cells to ferroptosis inducer, erastin

**Fig. 4:**
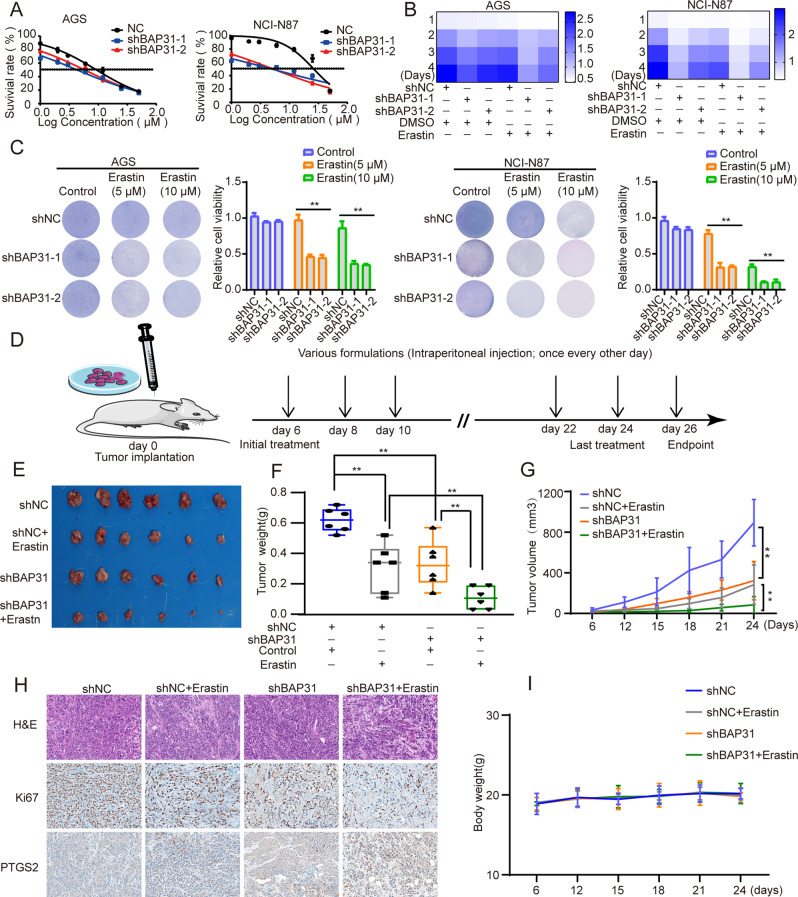
Knockdown of BAP31 sensitizes GC cells to ferroptosis inducer, erastin. **A** The IC50s of erastin were analyzed in AGS and NCI-N87 cells with BAP31 knockdown. **B** Heatmap demonstrated concerted reaction to 5 μM erastin combined with BAP31 attenuation in GC cells. **C** Clone formation assays exhibited concerted reaction to erastin (5 μM, 10 μM) combined with BAP31 attenuation in GC cells. **D** Subcutaneous xenograft model and injection schedule were formulated in nude mice. NCI-N87 cells transfected with shNC or shBAP31 were subcutaneously injected into the flanks of nude mice administrated without or with erastin, and representative images of dissected tumors (**E**), tumor weight (**F**) and tumor growth curves (**G**) of mice. **H** HE and IHC assay for Ki-67 and PTGS2 were performed in isolated tumor tissues. **I** Body weight was measured in the above-mentioned mice. *p < 0.05, **p < 0.01.

As a classical inducer of ferroptosis, erastin triggers ferroptosis by targeting numerous molecules including the cystine-glutamate transport receptor, VDAC, and p53 [29]. Erastin enhances therapeutic effects of chemotherapy and radiotherapy, which suggests an inspiring therapeutic strategy. As the above findings demonstrated that BAP31 knockdown activated ferroptosis, we further treated GC cells with erastin and found that cells with BAP31 knockdown were more sensitive to erastin (Fig. 4A). We discovered that BAP31 knockdown in combination with erastin amplified the inhibitory effects in cell growth induced by single treatment in AGS and NCI-N87 cells (Fig. 4B, C). Next, we established a xenograft model by injecting GC cells subcutaneously to further confirm the results in vivo (Fig. 4D). The results demonstrated that BAP31 knockdown or erastin (20 mg/kg) alone inhibited tumor growth significantly, where there was an enhancement in the anti-tumor effects once BAP31 attenuation and erastin treatment were combined (Supplementary Fig. S4A and Fig. 4E–G). Afterwards, the tumor tissues were isolated and subsequently an immunohistochemistry analysis was performed by staining Ki-67 and PTGS2, the marker for assessment of ferroptosis in vivo. The results showed that tumors from BAP31 knockdown group exhibited reduced expression of Ki-67, which was lower in the combination group, as expected (Fig. 4H and Supplementary Fig. S4B). The PTGS2 level increased with BAP31 knockdown, and was further elevated once it was combined with erastin treatment (Fig. 4H). Animal body weight did not change significantly, which indicated few adverse effects (Fig. 4I). Thus, our data provide persuasive evidence that targeting BAP31 and inducing ferroptosis may act as an efficient strategy in GC treatment.

### BAP31 diminishes ferroptosis by inhibiting VDAC1 expression via ubiquitin-proteasome pathway in GC cells

**Fig. 5:**
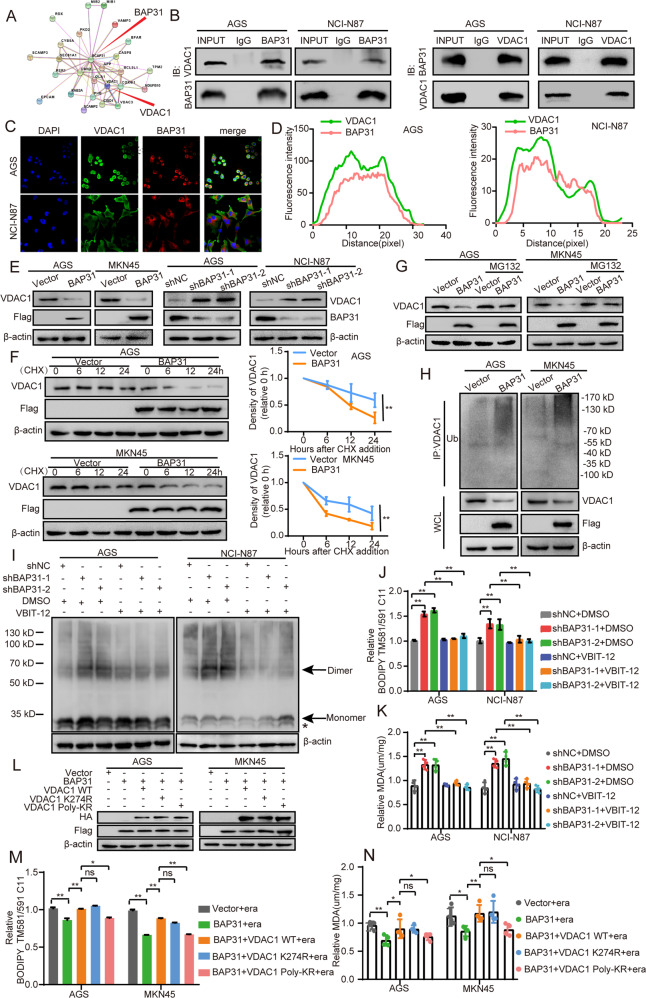
BAP31 interact with VDAC1 and affect its protein stability. **A** STRING database was utilized to predict proteins that interact with BAP31. **B** Immunoprecipitation was conducted to examine the relationship between BAP31 and VDAC1. **C**, **D** The interaction between BAP31 and VDAC1 were observed through immunofluorescence assays. **E** Western blot was used to assess the protein level of VDAC1 in BAP31-overexpressed or BAP31-knockdown GC cells. **F** BAP31 overexpression group and control group were pretreated with cycloheximide (CHX), then the protein level of VDAC1 was detected. **G** BAP31 overexpression group and control group were pretreated with MG132 6 h, then the protein level of VDAC1 was detected. **H** The ubiquitination of VDAC1 in BAP31 overexpression group and control group. **I** We attenuated BAP31 in AGS and NCI-N87, and treated with DMSO or VBIT-12 respectively. Then, cells in each group were harvested and incubated in the presence of ethylene glycol bis (succinimidyl succinate) to cross-link proteins and then subject to western blot to assess the oligomeric status of VDAC1. Arrows indicate monomer and dimer forms of VDAC1. Asterisk indicates the intramolecular cross-linked bands. BAP31 knockdown GC cells pretreated with erastin (5 µM) for 12 h in the presence or absence of 10 µM VBIT-12 (VDAC1 oligomerization inhibitor), where lipid ROS production (**J**) and MDA content (**K**) were assayed. **L** The expression of BAP31 and VDAC1 was detected in BAP31-overexpressing cells transfected with VDAC1 WT or two ubiquitination mutants (VDAC1 K274R and Poly-KR). BAP31-overexpressing GC cells transfected with VDAC1 WT or two ubiquitination mutants treated with erastin (5 µM) for 12 h, where lipid ROS production (**M**) and MDA content (**N**) were tested. ns.p > 0.05, *p < 0.05, **p < 0.01.

As an ER membrane protein, BAP31 could impair adaptation to ER stress conditions via forming a BAP31-STX17 protein complex [30]. In hepatocellular carcinoma, BAP31 promotes tumor proliferation via protein interaction with SERPINE2 [31]. To explore the mechanism through which BAP31 promotes GC development, we used the STRING database for identifying the interacting proteins of BAP31, which resulted in identification of VDAC1 as one of such proteins (Fig. 5A). VDACs are regarded as a multi-functional protein that regulate metabolic states in tumor cells, including Ca^2+^ homeostasis, oxidative stress, and the transport of metabolites between mitochondria and cytosol [32, 33]. We verified the interaction between BAP31 and VDAC1 in GC cells via co-immunoprecipitation (Co-IP) assay (Fig. 5B), while there was no interaction between BAP31 and VDAC2/3 (Supplementary Fig. S5A). And immunofluorescence assay also showed the co-location between BAP31 and VDAC1 in GC cells (Fig. 5C, D). Emerging evidence have shown that VDAC1 plays a critical role in the process of ferroptosis [34, 35]. We next evaluated whether BAP31 inhibited ferroptosis through regulation of VDAC1. We found that VDAC1 mRNA expression was unaffected by BAP31 (Supplementary Fig. S5B), while its protein level decreased under overexpression of BAP31 and increased with BAP31 knockdown (Fig. 5E). Therefore, we postulated that BAP31 might influence VDAC1 protein stability. A cycloheximide (50ug/ml) chase assay was performed that VDAC1 protein stability in GC cells got sabotaged under BAP31 overexpression compared to control cells (Fig. 5F). These results indicated that overexpression of BAP31 promoted VDAC1 degradation. Next, GC cells with BAP31 overexpression was treated with MG132. We found that BAP31-induced VDAC1 degradation got vanished in the presence of the MG132 (Fig. 5G). We also observed that BAP31 overexpression enhanced VDAC1 ubiquitination (Fig. 5H). These results demonstrated BAP31 reduced VDAC1 expression via the ubiquitin-proteasome pathway in GC cells.

To confirm the role played by VDAC1 in BAP31-inhibited ferroptosis, we explored the cell ferroptosis state of expressing VDAC1 in BAP31-overexpressing cells (Supplementary Fig. S5C). We found that BAP31 overexpression promoted cell proliferation and suppressed cell ferroptosis, which could be reversed by VDAC1 overexpression (Supplementary Fig. S5D–G). As VDAC1 oligomerization induced by VDAC1 overexpression played vital physiological roles in the regulation of VDAC1 function [36], we used VBIT-12 – a VDAC1 oligomerization inhibitor – to treat BAP31 knockdown cells. BAP31 knockdown increased VDAC1 oligomerization, which was counteracted by VBIT-12 (Fig. 5I). The elevated lipid peroxidation level caused by BAP31 knockdown was dramatically attenuated by VBIT-12 (Supplementary Fig. S5H and Fig. 5J, K), which suggested that VDAC1 oligomerization inhibitor prevented GC cells from BAP31 knockdown-induced VDAC1 oligomerization and ferroptosis. VDAC1 exhibited two types of ubiquitination: lysine 274 (K274) for VDAC1 monoubiquitnation and lysines 12, 20, 53, 109, and 110 (Poly-K) for VDAC1 polyubiquitination [37]. In order to determine which type of VDAC1 ubiquitination was affected by BAP31, we expressed VDAC1 WT, K274R, or Poly-KR in BAP31-overexpressing cells (Fig. 5L), where the lower lipid peroxidation level upon BAP31 overexpression could be reversed by VDAC1 WT and K274R, but not Poly-KR (Fig. 5M, N and Supplementary Fig. S5I). This indicated that BAP31 inhibited ferroptosis mainly through inducing VDAC1 polyubiquitination. Taken these observations together, it is concluded that BAP31 reduces VDAC1 expression via the ubiquitin-proteasome system.

### HNF4A upregulates BAP31 expression through binding at its promoter in GC

**Fig. 6:**
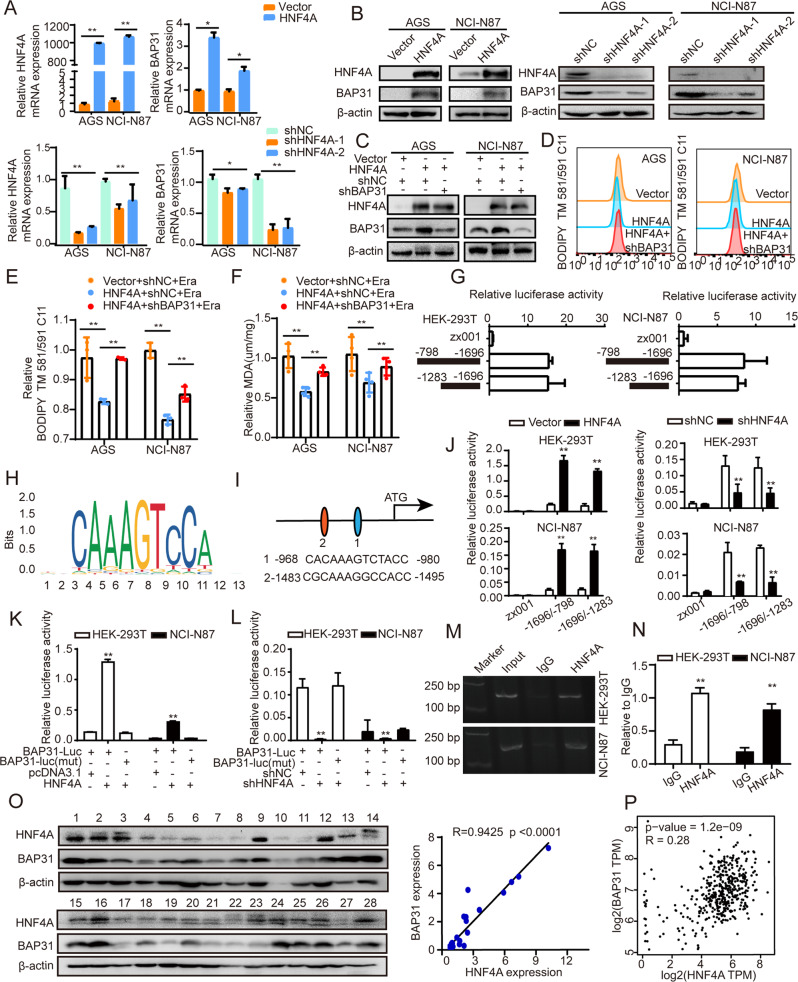
HNF4A binds at BAP31 promoter and augments BAP31 transcription. **A**, **B** The mRNA and protein level of BAP31 were detected in GC cells with HNF4A overexpression or knockdown. **C** The level of HNF4A and BAP31 was detected in HNF4A-overexpressing cells transfected with BAP31 shRNA. **D**–**F** Lipid ROS and MDA content were assayed in HNF4A-overexpressing cells treated with BAP31 shRNA treated with erastin (5 µM) for 12 h. **G** Relative luciferase activities were assessed in HEK-293T and NCI-N87 cells transfected with different truncations of BAP31 promoter. **H** HNF4A binding motif was predicted in JASPAR. **I** JASPAR analysis revealed two potential HNF4A-binding sites (scores >10) within the promoter region of BAP31. **J** HEK-293T and NCI-N87 cells transfected with different truncations of BAP31 promoter, in the presence of HNF4A overexpression or knockdown respectively, then relative luciferase activities were assessed. **K**, **L** Relative luciferase activities were assessed in HEK-293T and NCI-N87 cells treated with BAP31 luciferase reporter vectors (wild-type or mutant in HNF4A-binding sites, -1495/-1483 bp), in the presence of HNF4A overexpression or knockdown, respectively. **M** Agarose electrophoresis for ChIP analysis of HNF4A binding at BAP31 promoter. **N** ChIP-qPCR analysis of HNF4A binding at BAP31 promoter. **O** Western blot analysis of BAP31 and HNF4A expression in GC tissues (*n* = 28). **P** The relationship between HNF4A and BAP31 in GC tissues was analyzed using TCGA datasets (R = 0.28, *p* < 0.001). *p < 0.05, **p < 0.01.

There has been report that mir-451a binds to the BAP31 5’-UTR and decreases BAP31 expression, so as to suppress the proliferation in colorectal cancer (CRC) cells [38]. To explore the underlying mechanism responsible for its increase in GC tissues, we used JASPAR database for predicting the potential transcriptional regulators of BAP31. NRF2 closely involves in lipid peroxidation and ferroptosis [39, 40], and its binding sites are identified in the promoter of BAP31. Although there was a positive correlation between BAP31 and NRF2 in GC tissues, the mRNA and protein levels of BAP31 were barely altered under NRF2 knockdown (Supplementary Fig. S6A–C), which excluded the possibility of NRF2 involvement in BAP31 regulation. Besides, binding sites for HNF4A were also observed in the promoter of BAP31, which suppressed ferroptosis by affecting the synthesis of glutathione (GSH) [41]. The expression of BAP31 was increased in HNF4A-overexpressing cells, or decreased following HNF4A knockdown (Fig. 6A, B). To verify the role of HNF4A/BAP31 in ferroptosis and cell growth, HNF4A-overexpressing GC cells were transfected with BAP31 shRNA (Fig. 6C), which revealed that HNF4A-inhibited cell ferroptosis was reversed by BAP31 knockdown (Fig. 6D–F), and HNF4A-induced cell proliferation was attenuated by BAP31 silencing (Supplementary Fig. S6D–G).

To further explore the regulatory mechanism of BAP31, we constructed serial truncations of the BAP31 promoter and performed dual-luciferase reporter assay. We found that highest transcriptional activity within the −798~−1696 bp especially −1283~−1696 bp region in BAP31 promoter (Fig. 6G), where two putative HNF4A-binding sites were identified within these promoter regions (Fig. 6H, I). Luciferase assay showed that HNF4A overexpression increased the BAP31 promoter activity, whereas HNF4A knockdown impaired it (Fig. 6J). Furthermore, HNF4A barely increased the activity of the BAP31 promoter containing a mutated putative HNF4A binding site 2 (Fig. 6K, L). Furthermore, the ChIP assay suggested that HNF4A bound at the BAP31 promoter (Fig. 6M, N), which indicated a direct transcriptional regulation in GC cells. Western blot in human GC tissues revealed a distinct positive correlation between HNF4A and BAP31 protein level (Fig. 6O, R = 0.9425, *p* < 0.001). Furthermore, similar results were also presented using TCGA GC cohort (Fig. 6P). Thus, these results reveal that HNF4A enhances BAP31 level via binding at its promoter in GC cells.

### Suppression of BAP31 sensitizes GC cells to 5-FU and the united strategy of employing 5-FU with erastin amplifies anti-tumor effect

**Fig. 7:**
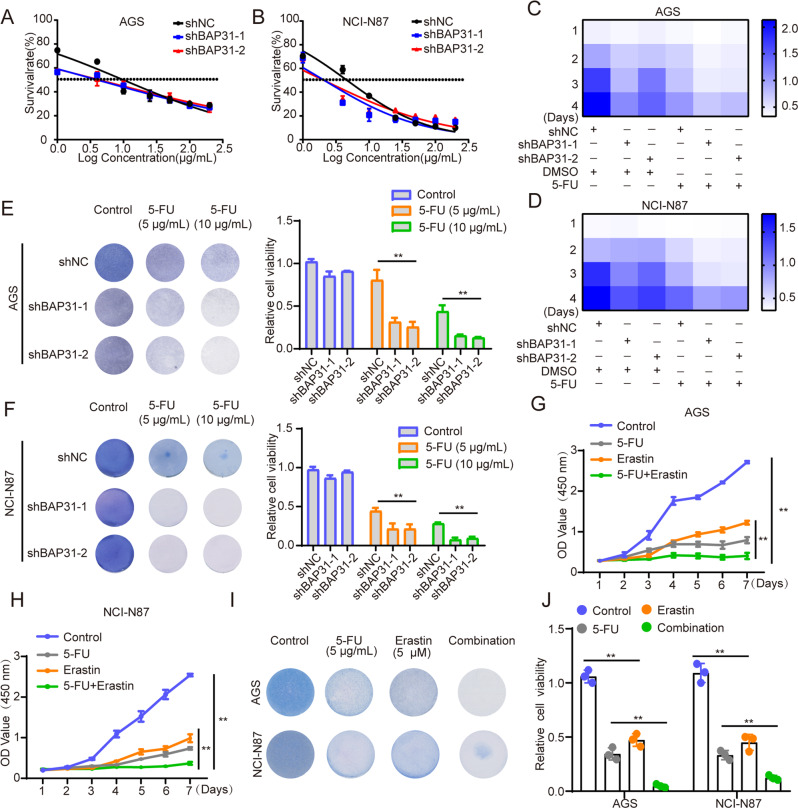
Knockdown of BAP31 sensitizes GC cells to 5-FU and combining 5-FU with erastin amplifies anti-tumor effects. **A**, **B** The IC50s of 5-FU in AGS and NCI-N87 cells with BAP31 knockdown were analyzed. **C**, **D** Heatmap demonstrated synergistic response to 5 μg/ml 5-FU combined with BAP31 knockdown in AGS and NCI-N87 cells. **E**, **F** Clone formation assays showed synergistic response to 5-FU (5 μg/ml, 10 μg/ml) combined with BAP31 knockdown in AGS and NCI-N87 cells. **G**–**J** CCK8 and clone formation assays showed combination of erastin with 5-FU treatment exhibited higher inhibitory effects on cell growth compared with mono treatment in AGS and NCI-N87. *p < 0.05, **p < 0.01.

Fluorouracil (5-FU) and cisplatin are classical treatments for GC, for which the drug resistance occurs frequently and leads to unfavorable outcome [42]. We speculated whether BAP31 influenced the therapeutic effects of 5-FU or cisplatin in GC cells. We found that BAP31 knockdown sensitized GC cells to 5-FU treatment (Fig. 7A, B), where it exerted little effect on the sensitivity to cisplatin treatment (Supplementary Fig. S7A, B). Intriguingly, the CCK8 and colony formation assays indicated that synergetic treatment of BAP31 knockdown and 5-FU exhibited stronger inhibitory effects on the cell growth (Fig. 7C–F). It has been reported that erastin overcomes ABCB1-mediated docetaxel resistance in ovarian cancer, offering an effective strategy for chemo-resistant patients [43]. The combined application of erastin with 5-FU treatment resulted in remarkable inhibitory effects on cell growth in AGS and NCI-N87 cells (Fig. 7G–J). Thus, these results suggest that ferroptosis induction could potentiate cytotoxic effect of 5-FU and may serve as a potential therapeutic strategy.

## Discussion

GC is considered as one of the most prevalent malignant carcinomas and aggressive disease, which severely threatened human health [44]. Although its total incidence is on the decline, there is an increasing tendency in the young population, where its overall outcome remains dismal [4]. Currently, carcinoembryonic antigen (CEA) and CA19-9 are most frequently used in GC patients for prognostic prediction. However, due to the insufficient specificity and sensitivity, novel and sensitive biomarkers are in urgent need. There have been reports for BAP31 as a prognosis biomarker in various kinds of cancer [11, 13, 45, 46]. This study showed that BAP31 is significantly increased in GC, and its high level in GC indicates a worse prognosis and BAP31 may serve as an independent prognostic factor. As GC is biologically and genetically heterogeneous [47], it still requires precise subtype identification for patient stratification and targeted therapies.

It has been widely reported that BAP31 may participate in the malignant development of cancer [10]. In cervical cancer, BAP31 knockdown inhibits metastasis and induces apoptosis to hinder cervical cancer progression [48]. Another study showed that BAP31 regulates cervical cancer cell proliferation via inducing cell cycle arrest and blockades metastasis via regulating cytoskeleton assemblage [13]. In CRC, silencing BAP31 suppresses cell proliferation by inducing ER stress [38]. BAP31 overexpression promotes HCC growth by stabilizing SERPINE2 [31]. This study illustrate that BAP31 promotes tumor growth in vitro and in vivo. There are several miRNAs identified to regulate BAP31 expression [38, 49]. This research has identified HNF4A as a direct regulator for transcriptional activation on BAP31, which gives a reasonable explanation for its upregulation in GC. This is in line with the facts that HNF4A acts as an oncogene to promote gastrointestinal adenocarcinomas [50], and may govern the ferroptosis [41]. HNF4A overexpression inhibited cell ferroptosis and enhanced cell proliferation, which could be reversed by treatment with BAP31 shRNA. Moreover, BAP31 and HNF4A positively correlated in GC tissues, which verifies the existence of HNF4A/BAP31 axis in GC development.

Anchored at the ER membrane, BAP31 could interact with multiple proteins and participate in a variety of biological processes [8, 51, 52]. Upon ER stress, CDIP1 is activated and its association with BAP31 is enhanced at the ER membrane, activating the mitochondrial apoptosis pathway [53]. There are findings suggesting that BAP31 interacts with translocon Sec61 and promotes the extraction from the ER membrane by Derlin-1 complex, leading to the cytoplasmic degradation of CFTRΔF508 [54]. In HeLa cells, BAP31 associates with class I MHC molecules, mediating the traffic exit from ER [55]. In HCC, BAP31 promotes tumor growth via interaction with SERPINE2 and may act as a potential therapeutic target [31]. The present study revealed that BAP31 directly interacts with VDAC1 and decreases VDAC1 protein level other than in transcriptional way. VDAC1 decayed more rapidly in BAP31-overexpressing group with the treatment of cycloheximide versus control group, while BAP31 overexpression induced VDAC1 degradation, which could be reversed by MG132. Furthermore, the ubiquitinated VDAC1 level was enhanced with BAP31-overepression. As an indispensable factor of mitochondrial function, VDAC1 holds control over energy sources, metabolism, and apoptosis [56]. VDAC1 also gets involved in crosstalk between ER and mitochondria, which also indicates a potential interaction with ER protein. In a mouse model of systemic lupus erythematosus, the inhibitor for VDAC 1/2/3 oligomerization attenuates mtDNA release, neutrophil extracellular traps, IFN signaling, and influences disease severity [57]. One recent study demonstrates that inhibiting VDAC1 alleviates ferroptosis and decreases mitochondria damage in acute liver injury [33]. In line with BAP31’s regulation in ferroptosis, we presumed that BAP31/VDAC1 complex promotes GC development through regulating ferroptosis and cell proliferation. Thus, we found that BAP31 overexpression-hindered ferroptosis could be reversed by VDAC1 WT and K274R, but not by Poly-KR, which indicated that BAP31 inhibited ferroptosis mainly through inducing VDAC1 polyubiquitination. The VDAC1 oligomerization inhibitor, VBIT-12, could reverse BAP31 knockdown-induced VDAC1 oligomerization and ferroptosis. To sum up, our studies imply that BAP31 directly degrades VDAC1 via ubiquitin-proteasome pathway, which affects VDAC1 oligomerization to regulate ferroptosis and cell growth.

Ferroptosis is a novel form of cell death, characterized by cytological changes and is accompanied by alterations in the level of ROS, lipid peroxidation and related factors such as GPX4, PTGS2 and ACSL4 [21]. Ferroptosis plays a crucial role in progression of various tumors, such as lymphocytoma, RCC and HCC [19, 41]. This study shows that BAP31 overexpression suppresses ferroptosis, while BAP31 knockdown facilitates ferroptosis. Ferroptosis could be induced by several compounds such as the experimental reagent erastin and the approved drug sorafenib [58], and ferroptosis inducers are widely being tested in clinical trials for cancer treatment and have achieved satisfactory outcomes [59]. Erastin enhances the sensitivity to chemotherapy and radiotherapy, and as drug resistance is inevitable in cancer patients, this characteristic offers a promising strategy in cancer therapy [29, 60]. It is also revealed that BAP31 attenuation tends to make GC more susceptible to erastin and chemotherapy 5-FU, partly due to activating ferroptosis. In the present study, the in vivo tumor xenograft model was established, and the combination of BAP31 ablation with erastin achieved a remarkable anti-tumor effect, thus implying a critical role to be played by ferroptosis induction in cancer treatment. Moreover, the combination of ferroptosis inducer and 5-FU treatment effectively inhibited tumor growth, which may act as a promising therapeutic strategy.

## Conclusions

**Fig. 8:**
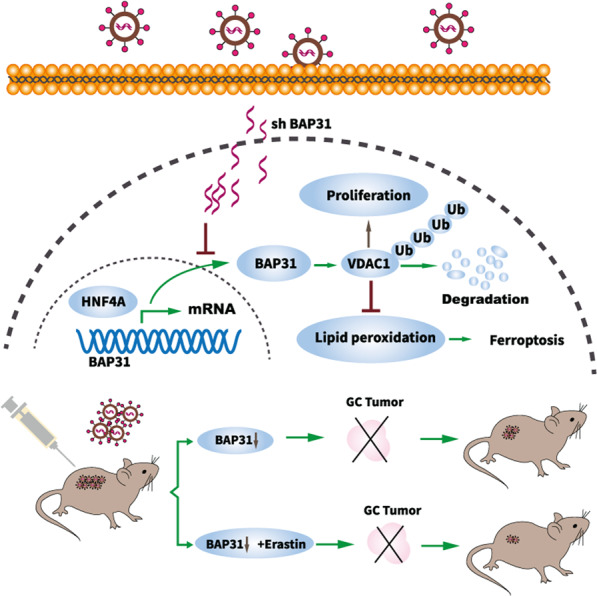
Schematic representation of HNF4A-BAP31-VDAC1 axis that synchronously regulates proliferation and ferroptosis in gastric cancer. HNF4A directly binds to BAP31 promoter and augments its transcription. BAP31 promotes cell proliferation and inhibits lipid peroxidation and ferroptosis. BAP31 overexpression directly induces VDAC1 protein degradation via the ubiquitin-proteasome pathway to promote GC progression. The shBAP31 impiars BAP31 translation and enhances the antitumor effects of ferroptosis inducer, erastin, which may serve as promising therapeutic strategy in anti -tumor treatment.

In summary, this work elucidates that BAP31 is upregulated in GC tissues and may serve as a prognostic factor for GC. The HNF4A-induced BAP31 interacts with VDAC1 and regulates cell proliferation and ferroptosis by inducing VDAC1 degradation. Furthermore, BAP31 knockdown could overcome chemotherapy drug resistance and its combination with ferroptosis inducer or chemotherapy may act as potential therapeutic strategies in GC (Fig. 8).

## Methods

### Cell lines and cell culture

NCI-N87, AGS and HEK-293T cell lines were obtained from the American Type Culture Collection (ATCC, Manassas, VA, USA). Other gastric cancer lines HGC27, SGC-7901, MKN45, MGC-803, BGC-823 and normal gastric cell line GES-1 were provided from the Chinese Academy of Science. All cells were cultured according to the standard protocol.

### Plasmids

BAP31 with flag tag was purchased from Genomeditech (Shanghai, China) and HNF4A plasmid was cloned into the pLX304-Blast-V5 vector. The BAP31 shRNA was obtained from IGEbio (Guangzhou, China). The NRF2 shRNA was purchased from GeneCopoeia (Guangzhou, China). The HNF4A shRNA was cloned into the pLKO.1 vector. VDAC1 WT (wide type) and two ubiquitination mutants (VDAC1 K274R and Poly-KR) with HA tag were obtained from IGEbio (Guangzhou, China). The target sequences are shown in Supplementary Table 1.

### Cell proliferation

Cell Counting Kit-8 (CCK8) (Bimake, USA), colony formation assays and EdU Cell Proliferation Kit (Beyotime) were used to evaluate GC cell proliferation.

### Immunohistochemistry (IHC) staining and evaluation

A tissue microarray containing 159 GC tissues was utilized for immunohistochemical assay. IHC assays and scores were conducted as reported previously [61]. This study was approved by the Ethics Committee of Ruijin Hospital, Shanghai Jiao Tong University School of Medicine. Antibodies used for IHC are shown in Supplementary Table 3.

### ROS assay and lipid peroxidation assessment, and measurement of intracellular MDA

Cells in appropriate density were evenly planted in 6 cm dishes overnight and then were pre-treated with erastin for 12 h incubated with 10 mM DCHF-DA (Thermo Fisher Scientific) or were pre-treated with erastin for 12 h and incubated with 10 μM C11-BODIPY (DojinDo) for 1 h. Before analyzing through flow cytometry, cells were washed with HBBS twice. FlowJo v10 was applied to analyze results.

For confocal analysis, cells were pre-treated with erastin for 12 h, then incubated with 3 μM C11-BODIPY for 1 h. Before detecting through microscope, cells should be washed twice. Lipid Peroxidation MDA Assay Kit (Beyotime) was applied to detect the MDA level, which reflected the level of lipid oxidation.

### Immunofluorescence confocal imaging

Cells were fixed in 4% paraformaldehyde at room temperature, and were permeabilized with PBS containing 0.1% Triton X-100 for 30 min. After that, cells were first incubated with primary antibodies overnight, then secondary antibodies and DAPI for 1 h at room temperature. Related antibodies are provided in Supplementary Table 3.

### Western blot and Co-immunoprecipitation (Co-IP) assay

Western blot and Co-IP assays were conducted as previously described [62]. Related antibodies are shown in Supplementary Table 3.

### Luciferase assay

The BAP31 promoter and 5’-truncated sequences of the BAP31 promoter were supplied by IGEbio (Guangzhou, China). Briefly, each experiment was performed with co-transfected cells expressing the corresponding reporter plasmid or indicated plasmids. The Dual-Luciferase Report Assay (Promega) system was applied to monitor luciferase activity.

### Chromatin immunoprecipitation (ChIP) assay

ChIP assays were conducted using Millipore Kit. Firstly, we crosslinked GC cells in 1% formaldehyde for 10 min before being quenched with 1× glycine. In order to break down the DNA to sizes between 200 and 1000 bases, the cell lysates were sonicated. The immunoprecipitation of DNA-containing complexes was carried out using the anti-HNF4A antibody or mouse IgG. As a result of reverse crosslinking of protein/DNA complexes with free DNA and qRT-PCR, the HNF4A-binding site within BAP31 promoter was detected. Supplementary Table 2 shows the primer sequences.

### Xenograft experiments

NCI-N87-shNC or NCI-N87-shBAP31 cells were used to establish a nude mouse model. The mice were randomly divided into several groups after injection and treated with control vehicles (PBS) or Erastin (Selleck) (20 mg/kg intraperitoneal injection every other day). Shanghai Medical Experimental Animal Care Committee guidelines were strictly followed in all mouse experiments and National Academy of Sciences and National Institutes of Health guidelines were followed.

### VDAC1 cross-linking assay

In order to determine varying VDAC1 oligomers, cellular chemical cross-linking was performed as described previously [31], using a membrane permeable cross-linker, EGS. Briefly, GC cells were treated as indicated, twice-washed through PBS, harvested by scraping, and placed into incubation in PBS (pH 7.4) at room temperature for 30 min. Then, 1.5 M Tris HCl (pH 7.8) was introduced (20 mM final concentration) and placed into incubation for 5 min at room temperature. Such mixture underwent centrifugation (10,000 × *g* for 5 min), followed by pellet undergoing lysis within iced NP-40 lysis buffer. Samples (50 μg) were diluted within relevant buffer and underwent SDS-PAGE/western blot through anti-VDAC1 antibody.

### Statistical analysis

We present all data as the mean ± standard deviation. In the case of multiple comparisons, Student’s *t* test with two tails or one-way ANOVA was used for statistical comparisons. A chi-square test was used to analyze the correlation between the expression level of BAP31 and the clinical characteristics of GC. The Kaplan–Meier method was used to calculate overall survival curves, and the log-rank test was used to compare them. The Cox proportional hazard regression model was used in a stepwise manner for analyzing univariate and multivariate data. An analysis of all statistical data was conducted with SPSS 23.0. Statistics were considered significant when a two-tailed *P*-value was less than 0.05.

## Supplementary information

Supplementary data.

Original Data File

checklist

## Data Availability

The datasets used and/or analyzed, and materials used during the current study are available from the corresponding author on reasonable request.
