# Supplementary material for: HNF4A-BAP31-VDAC1 axis synchronously regulates cell proliferation and ferroptosis in gastric cancer
Source: Cell Death Dis. 2023 Jun 9;14(6):356. doi: 10.1038/s41419-023-05868-z (PMC10256786; doi:10.1038/s41419-023-05868-z)
Supplement: Supplementary file 1 — Supplementary data. [file 41419_2023_5868_MOESM1_ESM.docx]

#### Table S1. The sequences of shRNA Target

| Identifier | Forward(5’-3’) |
| --- | --- |
| BAP31-1 | CGCCTGGTGACTCTCATTT |
| BAP31-2 | CGATGCCGTGCGCGAAATT |
| NRF2-1  NRF2-2  HNF4A-1  HNF4A-2 | GGAGTAAGTCGAGAAGTATTT  GGGATATGGTACAACCCTTGT  GCAGGAACATATGGGAACCAA  CGAGCAGATCCAGTTCATCAA |

**Supplementary Table S2.**The sequences of gene-specific primers used for qRT-PCR, vector constructs and ChIP assay

| Gene name | Forward(5’-3’) | Reverse (5’-3’) |
| --- | --- | --- |
| **Primers for qRT-PCR**  BAP31 CGGCTGGTGGAGTTGTTAGT    HNF4A CGAAGGTCAAGCTATGAGG  ACA  NRF2 AATTCAGCCAGCCCAGCACA  VDAC1 CGAGTGACCCAGAGCAACTT  GAPDH AGAAGGCTGGGGCTCATTTG  **Primers for ChIP**  BAP31  promoter GCCCGAAGCAGGGTGTAG  for HNF4A | | GATTCCTCTGGGCACGGAAA  ATCTGCGATGCTGGCAATCT  GATTTGGGAATGTGGGCAACC  TACTGTTTCCTGCGGTCCAG  AGGGGCCATCCACAGTCTTC  CCTACTACCGGGTCAGCAA |

**Supplementary Table 3.** Antibodies used in this study

| Antibody | catalog | Dilution | Company |
| --- | --- | --- | --- |
| For Western blotting | | | |
| BAP31  Flag-tag | sc-393810  TT0003 | 1:200  1:1000 | Santa Cruz  Abmart |
| P-p38 | CST-4511 | 1:500 | CST |
| p38 | CST-8690 | 1:500 | CST |
| PCNA | ab29 | 1:500 | Abcam |
| Cyclin D1 | ab226977 | 1:500 | Abcam |
| CDK4 | sc-56277 | 1:200 | Santa Cruz |
| CDK6 | sc-7961 | 1:200 | Santa Cruz |
| Rb | sc-102 | 1:500 | Santa Cruz |
| p-Rb (Ser807/811)  HA-tag | CST-8516  M20003 | 1:500  1:1000 | CST  Abmart |
| VDAC1  HNF4A | Ab15895  sc-374229 | 1:2000  1:500 | Abcam  Santa Cruz |
| β-actin | A3854 | 1:10000 | Sigma |
| Secondary antibody | HRP conjugated goat anti-rabbit IgG | 1:3000 | Sigma |
| Secondary antibody | HRP conjugated goat anti-mouse IgG | 1:3000 | Sigma |
| For Immunohistochemistry | | | |
| BAP31 | sc-393810 | 1:100 | Santa Cruz |
| Ki-67  PTGS2 | GT209407  Ab179800 | 1:50  1:50 | DAKO Abcam |
| Secondary antibody | Envision kit (HRP, rabbit/mouse, DAB+) | Ready-to-use | DAKO |
| For Immunofluorescence staining | | | |
| p38 | CST-8690 | 1:50 | CST |
| Secondary antibody | Alexa Fluor 594 anti-rabbit IgG | 1:50 | Invitrogen |

**Supplementary Figures**

A
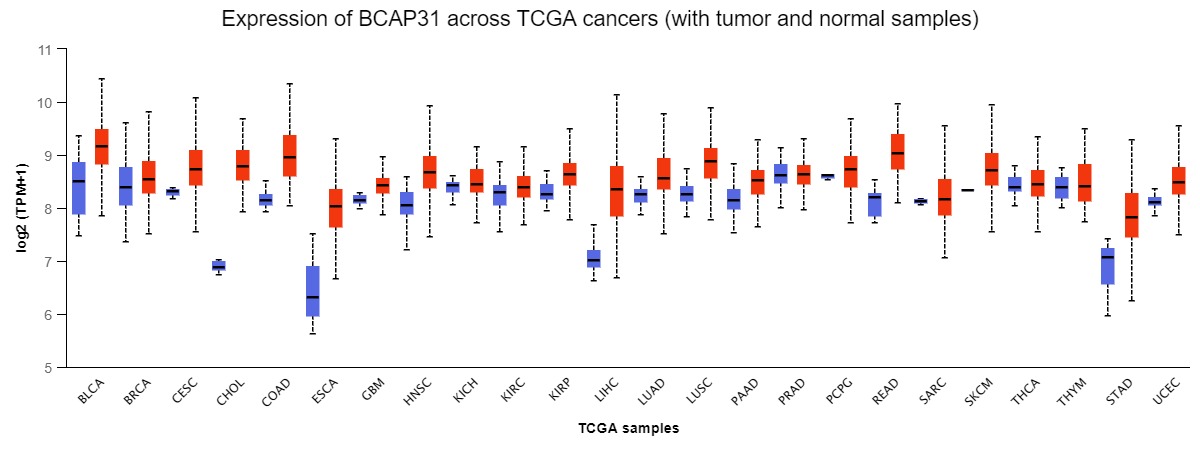


B


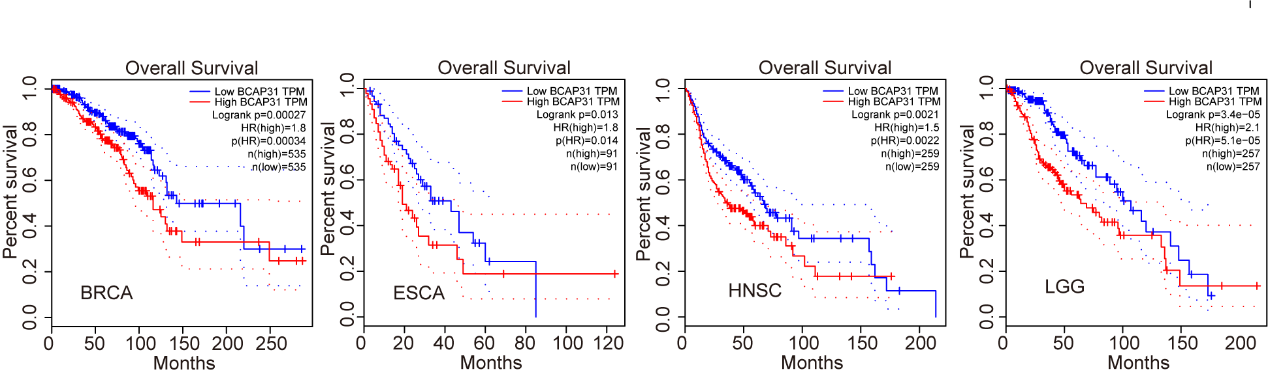


**Figure S1.** (A) The expression of BAP31 in tumor tissues compared with corresponding noncancerous tissues was analyzed using TCGA datasets. (B) Cancer patients with high expression levels of BAP31 had shorter overall survival than patients with low expression levels, as determined using TCGA datasets. BLCA: Bladder Cancer, BRCA: Breast Cancer, CESC: Cervical Cancer, CHOL: Bile Duct Cancer, COAD: Colon Cancer, ESCA: Esophageal Cancer, GBM: Glioblastoma, HNSC: Head and Neck Cancer, KICH : Kidney Chromophobe, KIRC: Kidney Clear Cell Carcinoma , KIRP: Kidney Papillary Cell Carcinoma, LIHC: Liver Cancer, LUAD: Lung Adenocarcinoma , LUSC: Lung Squamous Cell Carcinoma, PAAD: Pancreatic Cancer, PRAD: Prostate Cancer, PCPG: Pheochromocytoma & Paraganglioma, READ: Rectal Cancer, SARC: Sarcoma, SKCM: Melanoma, THCA: Thyroid Cancer, THYM, Thymoma, STAD: Stomach Cancer, UCEC: Endometrioid Cancer.


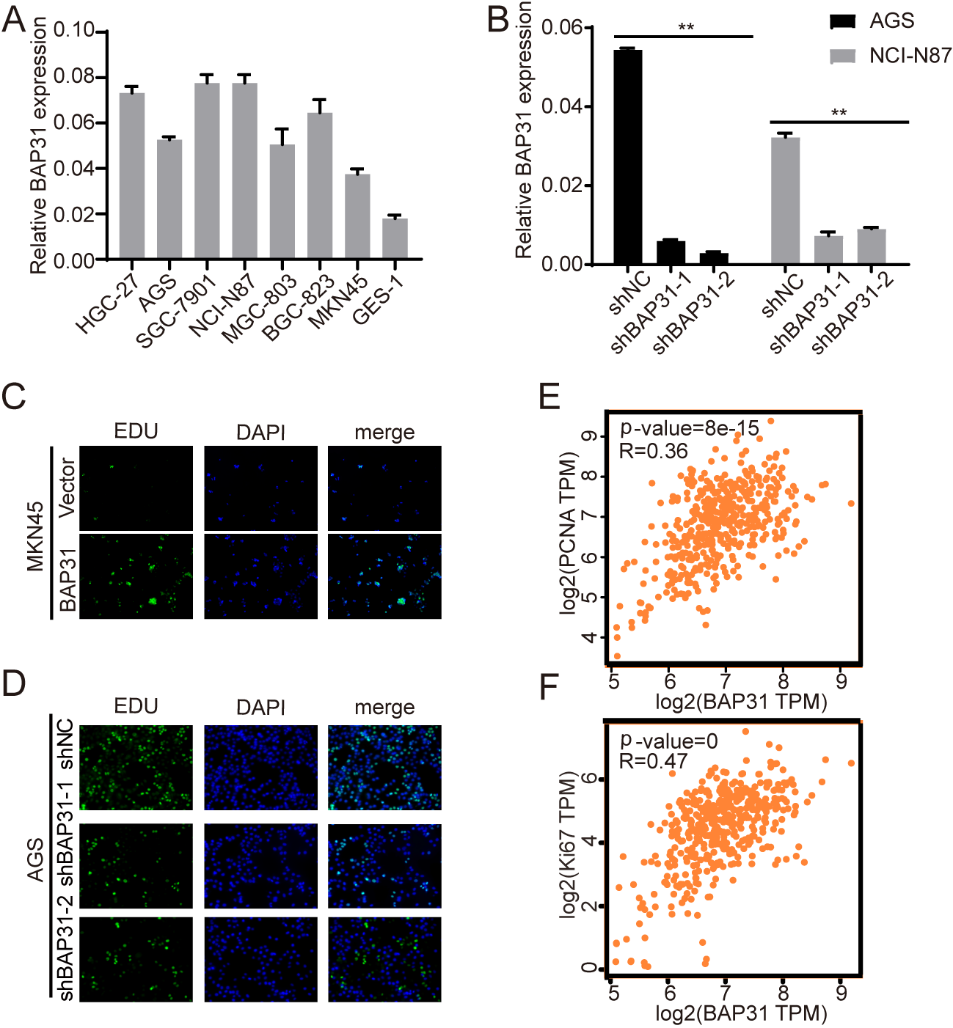


**Figure S2.** (A) The expression of BAP31 in GC cell lines were detected by qRT-PCR. (B) The expression of BAP31 was determined by qRT-PCR in GC cells with BAP31 knockdown. (C) Representative images of EdU incorporation assayin GC cells with BAP31 overexpression. (D) Representative images of EdU incorporation assay in GC cells with BAP31 knockdown. (E-F) The correlation of BAP31 and PCNA or Ki-67 expression in GC tissues using TCGA datasets. ***p* < 0.01.


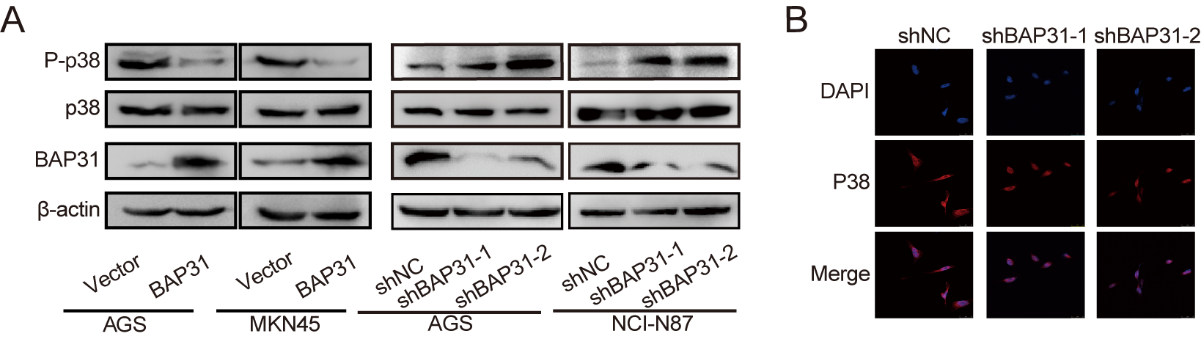


**Figure S3.** (A) The expression of P-p38 and P38 were detected by western blot in GC cells with BAP31 overexpression or knockdown. (B) Nuclear location of P38 was observed through immunofluorescence assays in AGS under BAP31 knockdown.


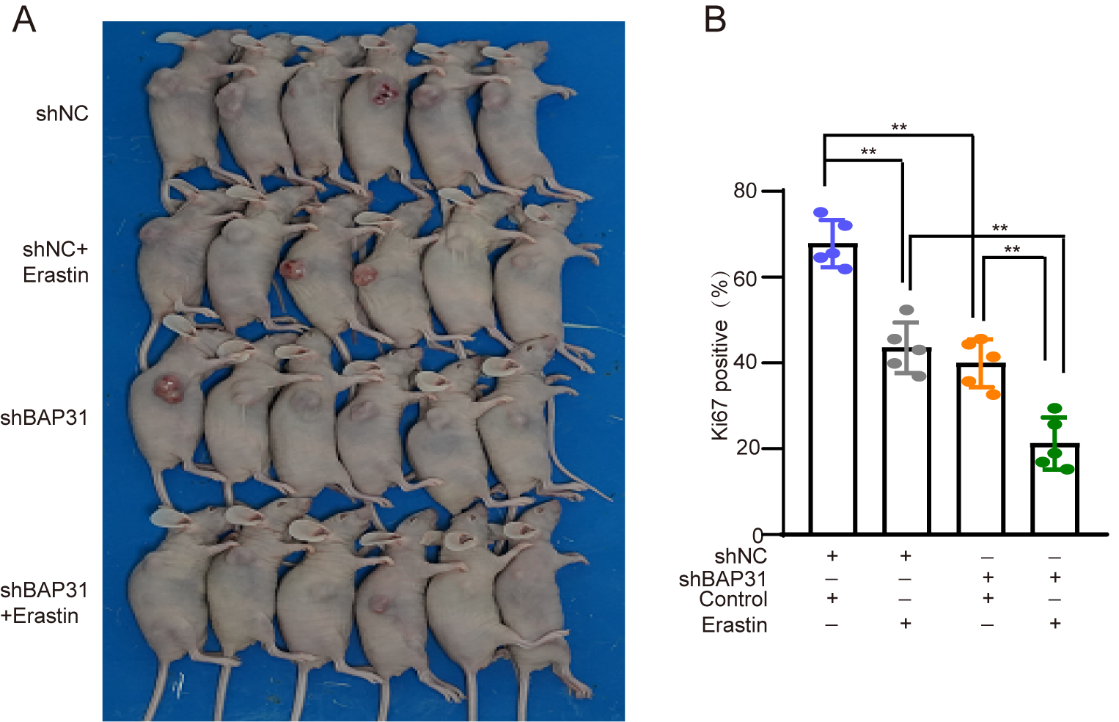


**Figure S4.** (A) Representative mice in each group were shown. (B) The results of Ki67 positive (%) in xenograft tumors derived from NCI-N87 cells with shNC or shBAP31 administrated without or with erastin. ***p* < 0.01.


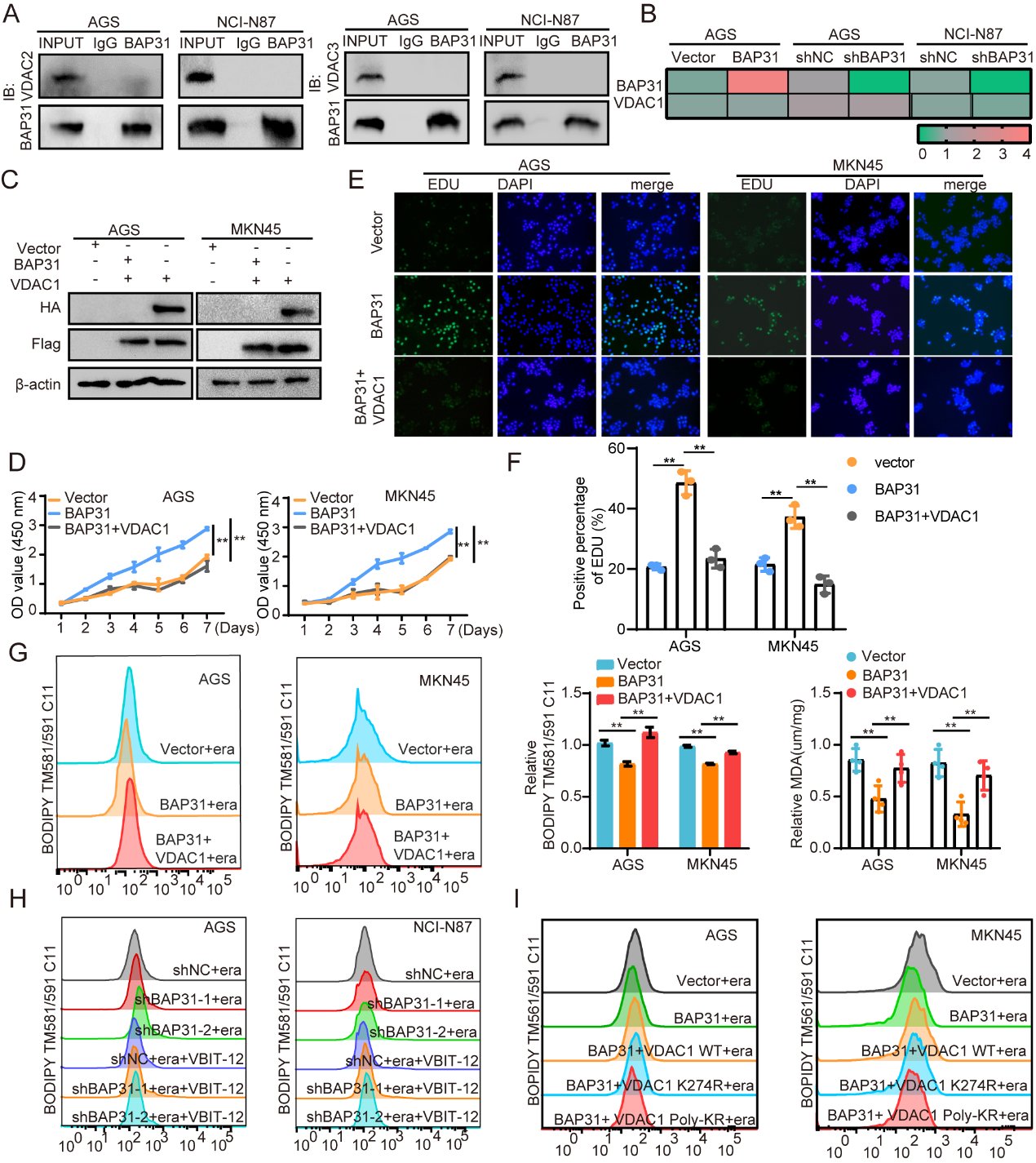


**Figure S5.** (A) Immunoprecipitation was conducted to examine the relationship between BAP31 and VDAC2 or VDAC3. (B) The qRT-PCR was used to evaluate the mRNA level of VDAC1 with BAP31-overexpressed or BAP31-knockdown. (C) The level of HA-VDAC1 and Flag-BAP31 was detected in BAP31-overexpressing cells transfected with HA-VDAC1 plasmid. (D) The effect of BAP31-overexpressing cells transfected with HA-VDAC1 plasmid on GC cell proliferation were assessed by the CCK8 assay. (E) EdU assay was used to assess growth on GC cells with BAP31-overexpressing cells transfected with HA-VDAC1 plasmid. (F) The positive percentage of EDU (%) on GC cells with BAP31-overexpressing cells transfected with HA-VDAC1 plasmid. (G) BAP31-overexpressing cells treated with VDAC1 plasmid treated with erastin (5 µM) for 12h, then lipid ROS production and intracellular MDA were assayed. (H) BAP31 knockdown GC cells treated with erastin (5 µM) for 12h in the presence or absence of 10 µM VBIT-12(VDAC1 oligomerization inhibitor), then lipid ROS production was assayed. (I) BAP31-overexpressing GC cells transfected with VDAC1 WT or two ubiquitination mutants treated with erastin (5 µM) for 12h, then lipid ROS production was assayed. ***p* < 0.01.


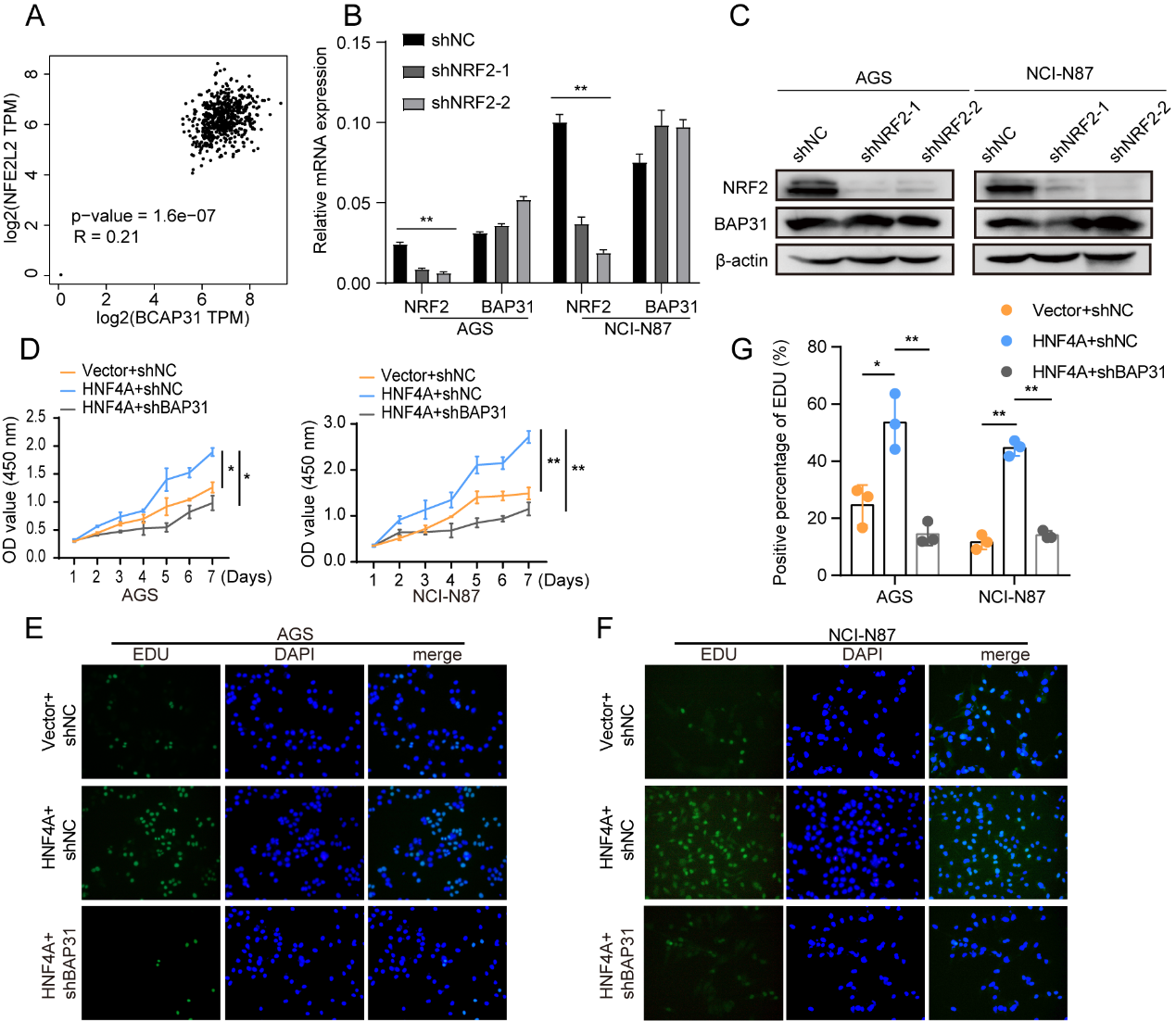


**Figure S6.** (A) The correlation of BAP31 and NRF2 in GC tumor tissues using TCGA datasets. (B-C) The expression levels of NRF2 and BAP31 was determined by qRT-PCR or western blot in GC cells with NRF2 knockdown. (D) The effect of HNF4A-overexpressing cells treated with BAP31 shRNA on GC cell proliferation were assessed by the CCK8 assay. (E-F) EdU assay was used to assess growth on GC cells with HNF4A-overexpressing cells treated with BAP31 shRNA. (G) The positive percentage of EDU (%) on GC cells with HNF4A-overexpressing cells treated with BAP31 shRNA. **p* < 0.05,***p* < 0.01.


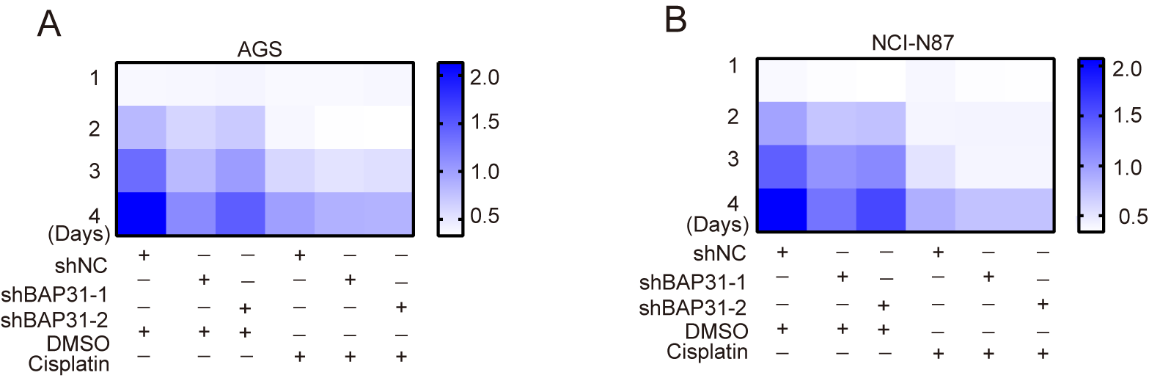


**Figure S7.** Knockdown of BAP31 barely affected cisplatin sensitivity in AGS cells (A) and NCI-N87(B) cells.
