# Supplementary material for: HNF4A-BAP31-VDAC1 axis synchronously regulates cell proliferation and ferroptosis in gastric cancer
Source: Cell Death Dis. 2023 Jun 9;14(6):356. doi: 10.1038/s41419-023-05868-z (PMC10256786; doi:10.1038/s41419-023-05868-z)
Supplement: Supplementary file 2 — Original Data File [file 41419_2023_5868_MOESM2_ESM.docx]

Fig 1D


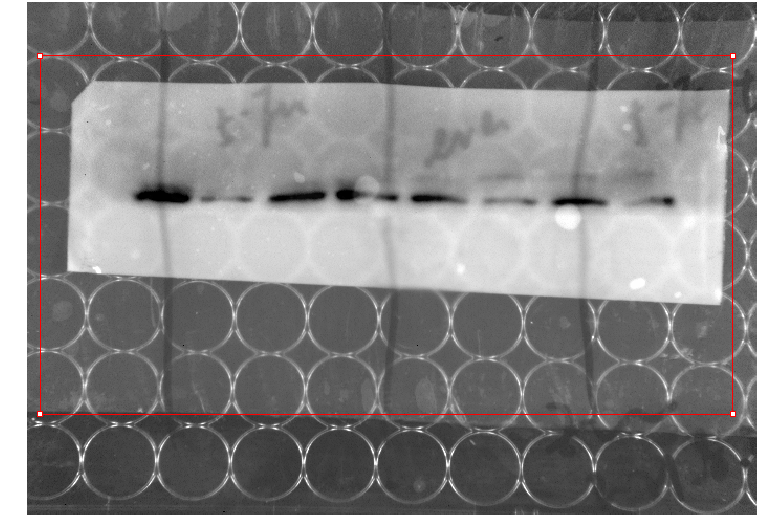
 BAP31（28kDa）


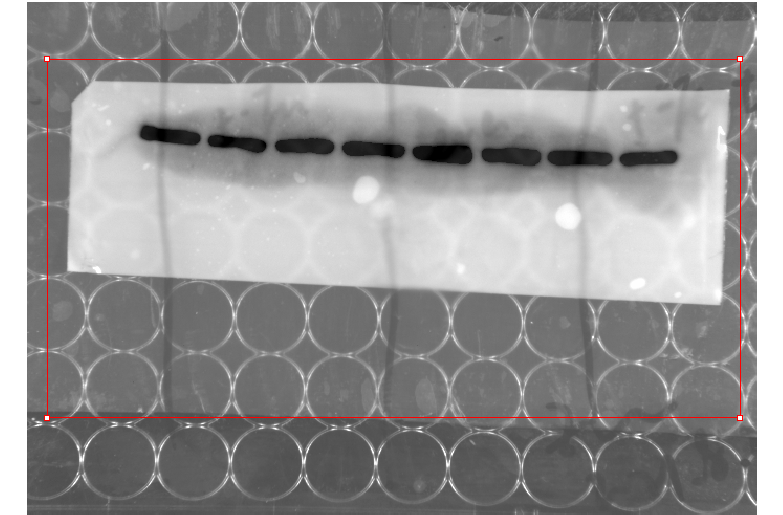
 β-actin（42KDa）


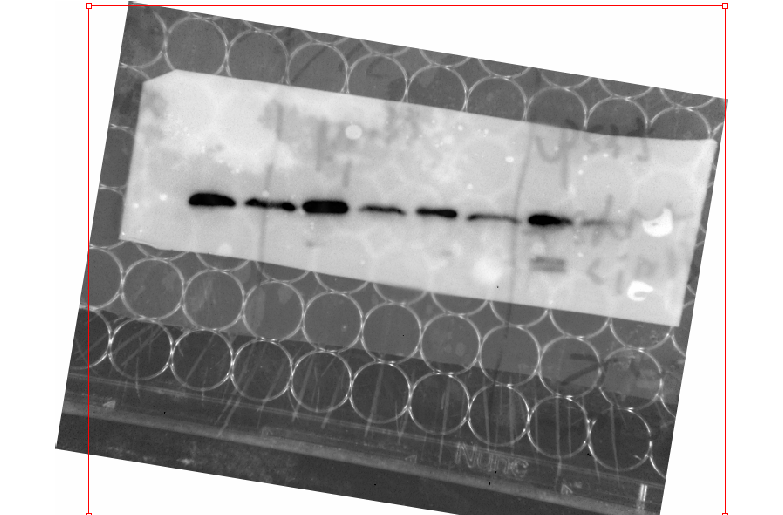
BAP31（28kDa）


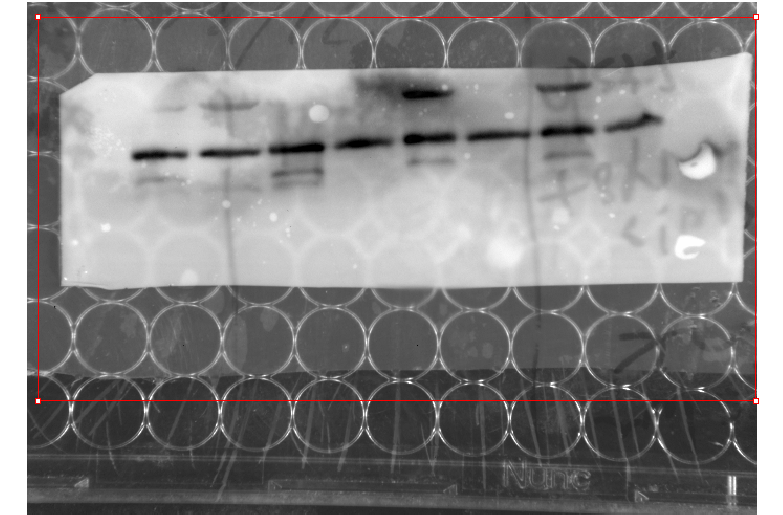
β-actin（42 kDa）

Fig 2A


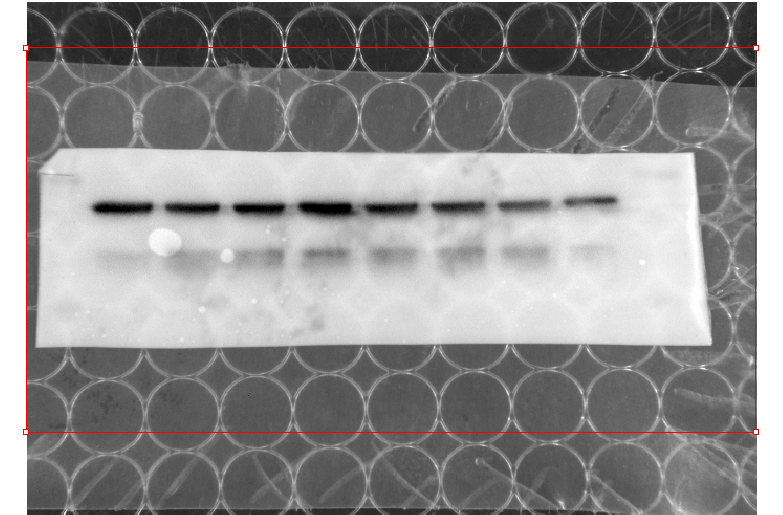
 BAP31（28kDa）


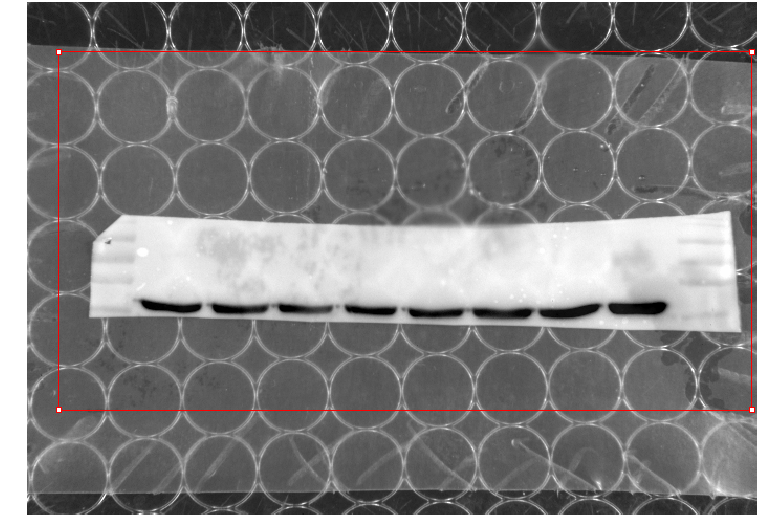
 β-actin（42KDa）

Fig 2B


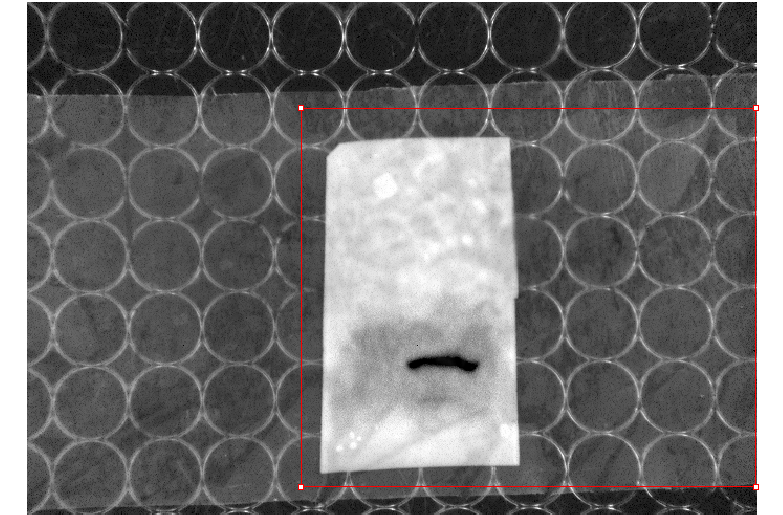
 Flag-BAP31（28kDa）
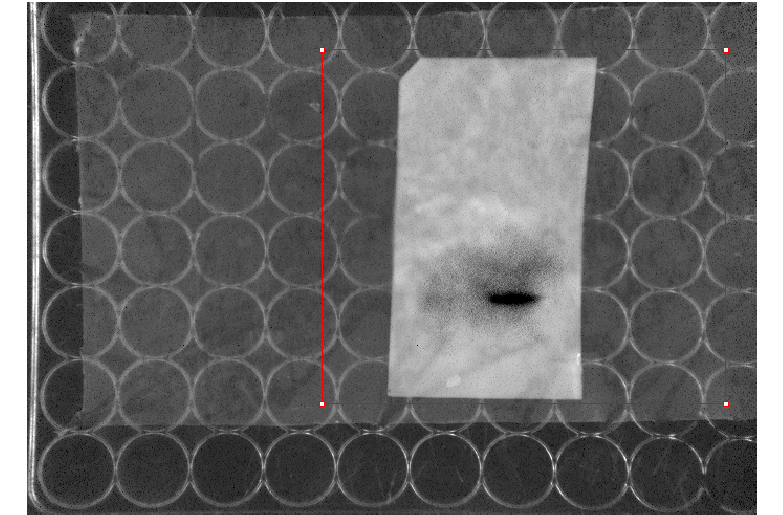
 Flag-BAP31（28kDa）


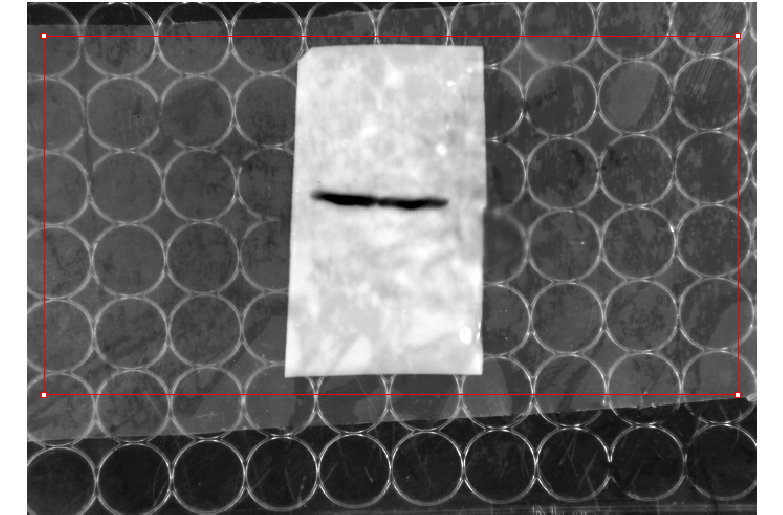
 β-actin（42KDa）
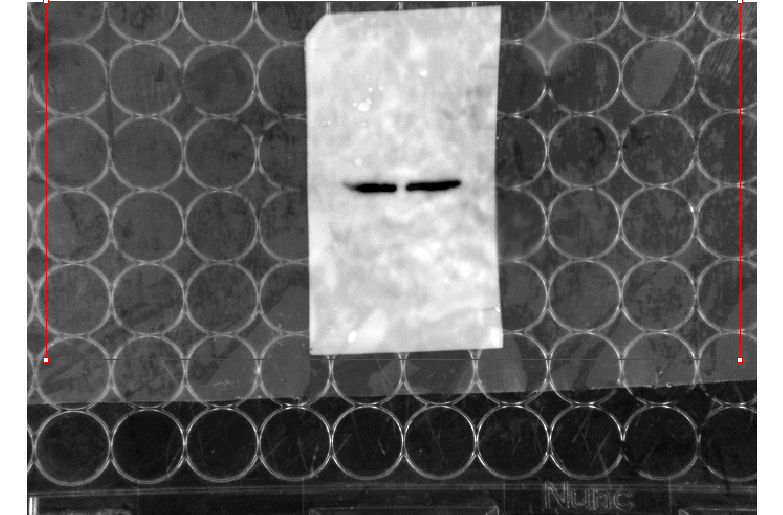
 β-actin（42KDa）

Fig 2E


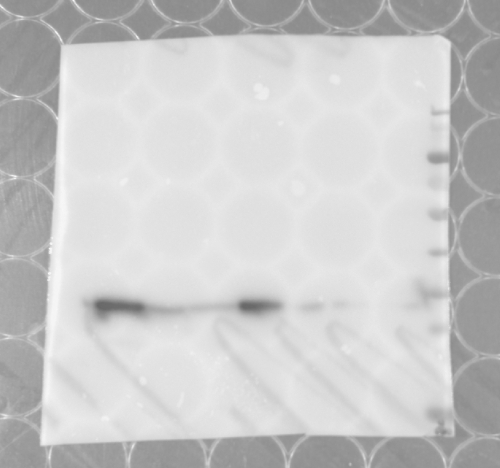
 BAP31（28kDa）


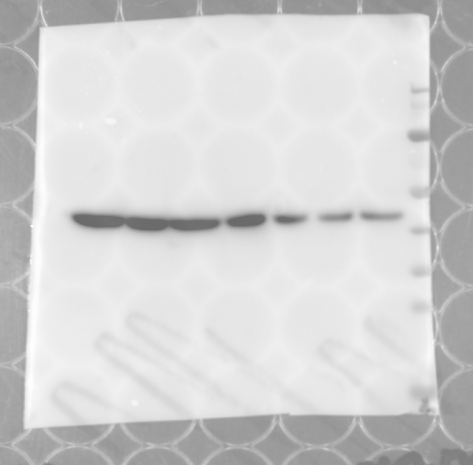
β-actin（42KDa）

Fig 2I


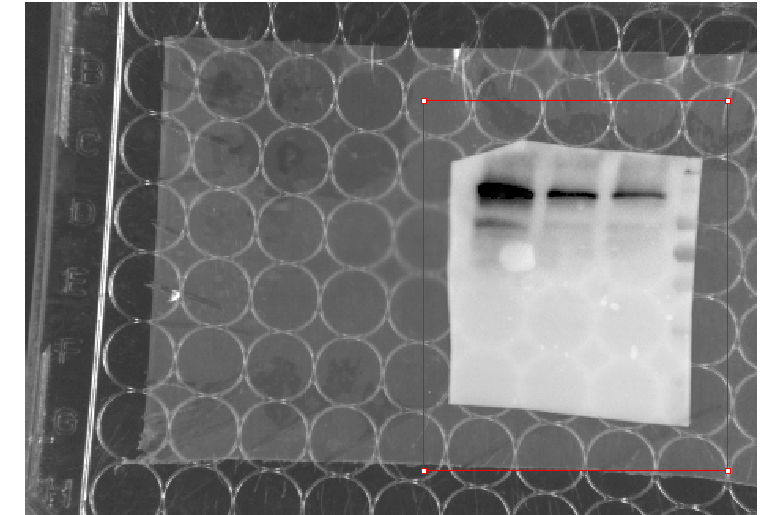
 p-Rb(110kDa)
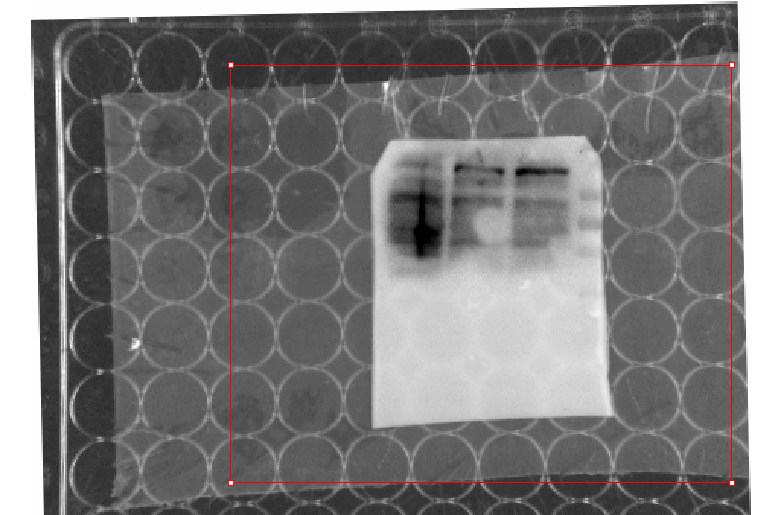
p-Rb(110kDa)


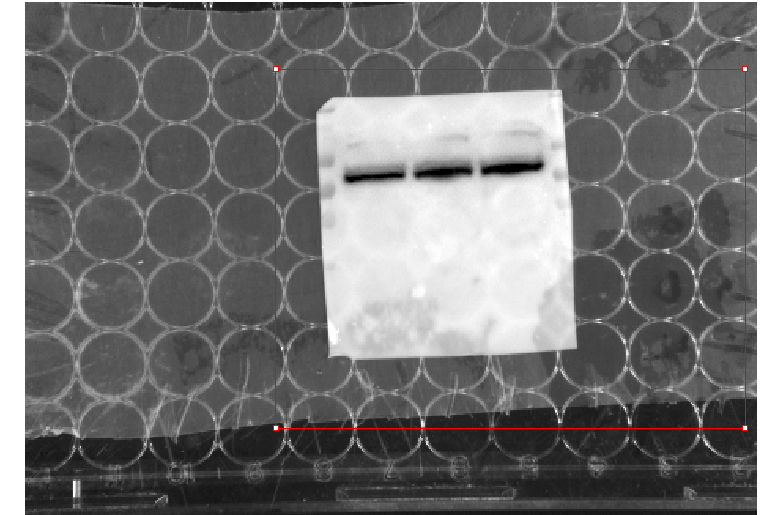
 Rb(106 KDa)
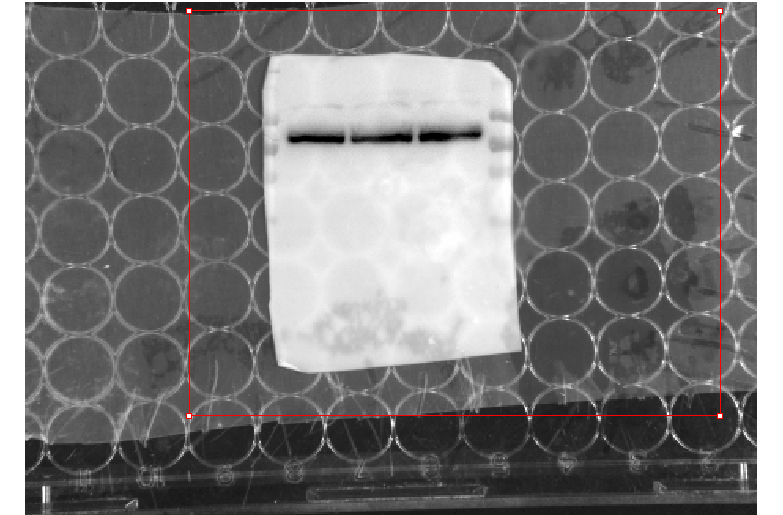
 Rb(106 KDa)


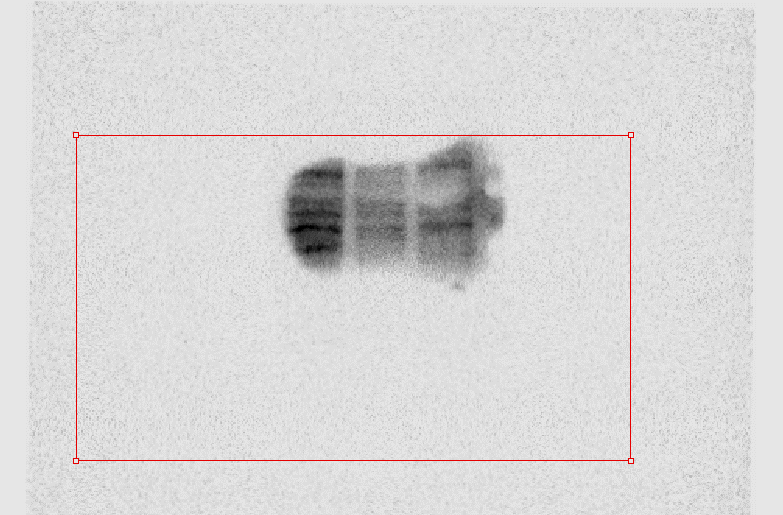


CyclinD1(37 kDa）
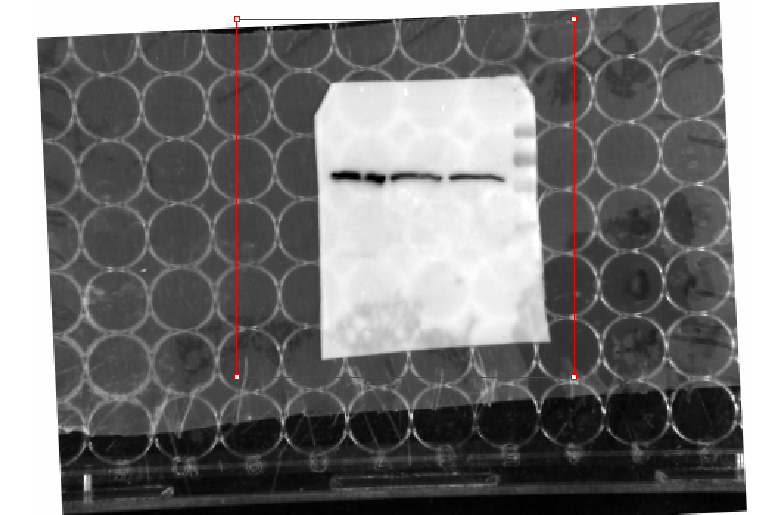
 CyclinD1(37 kDa）


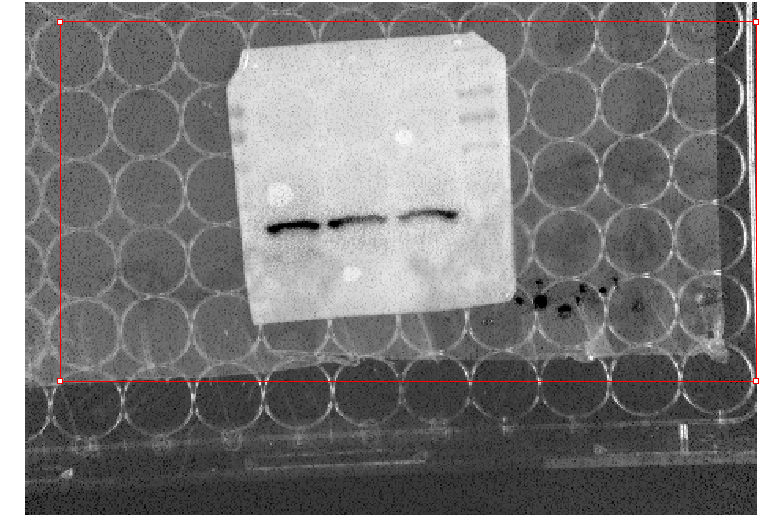
 CDK6(40 kDa)
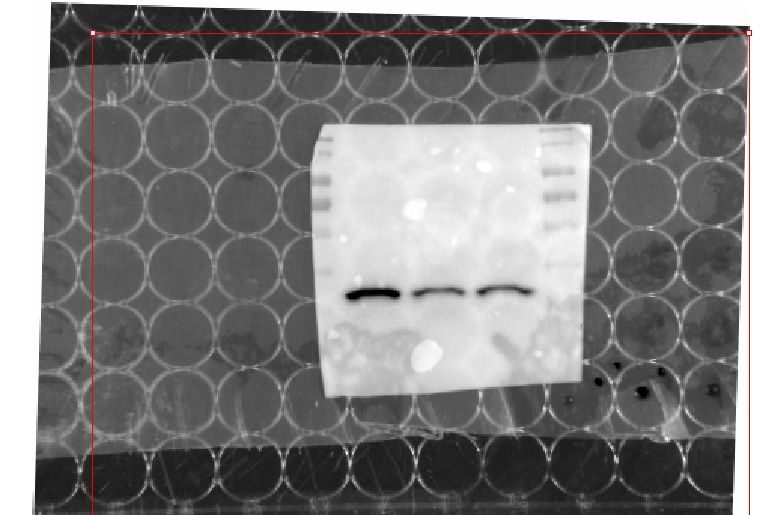
 CDK6(40 kDa)


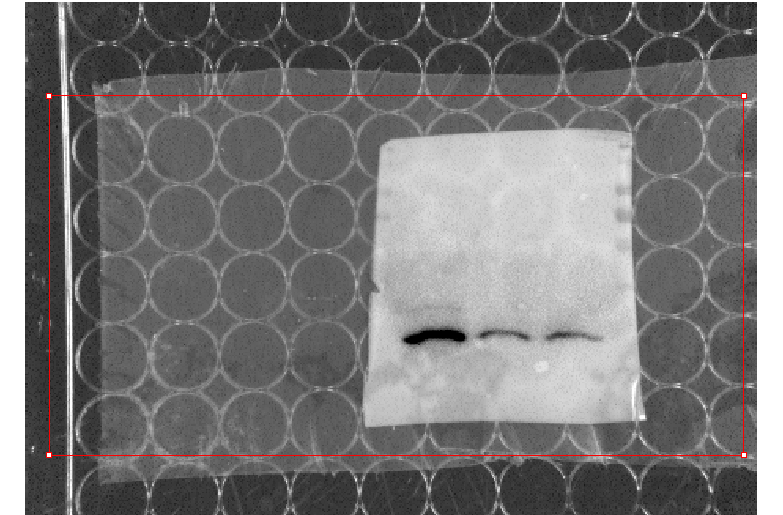
CDK4(34 kDa)
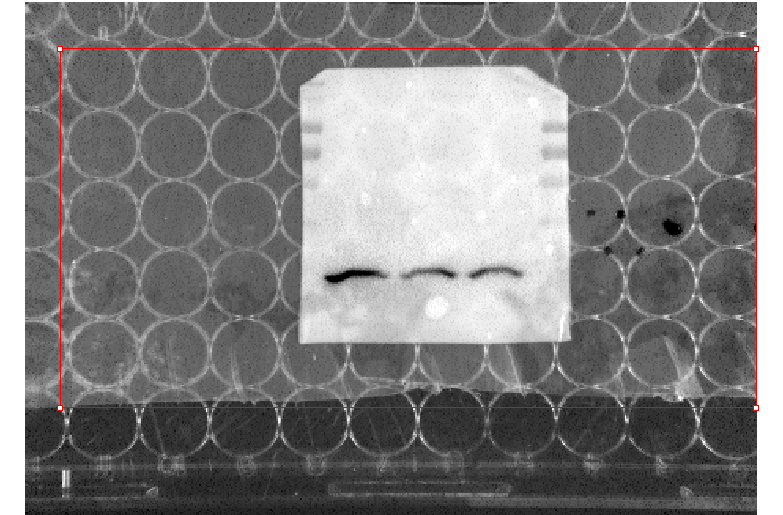
 CDK4(34 kDa)


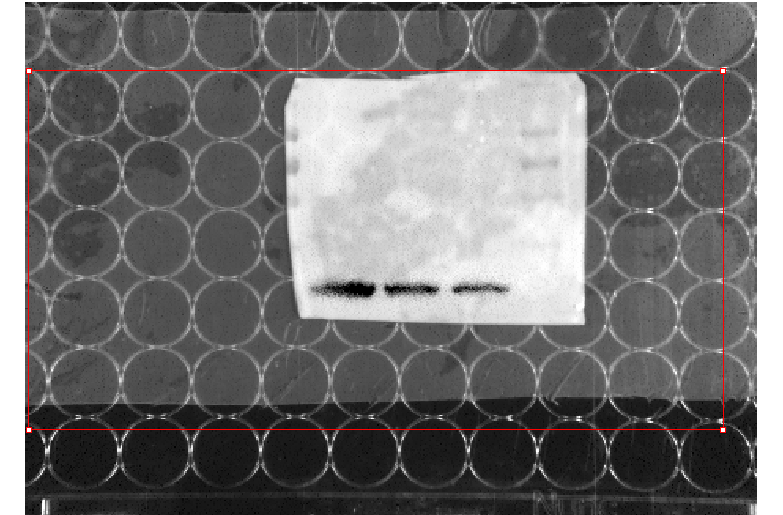
PCNA（29 kDa）
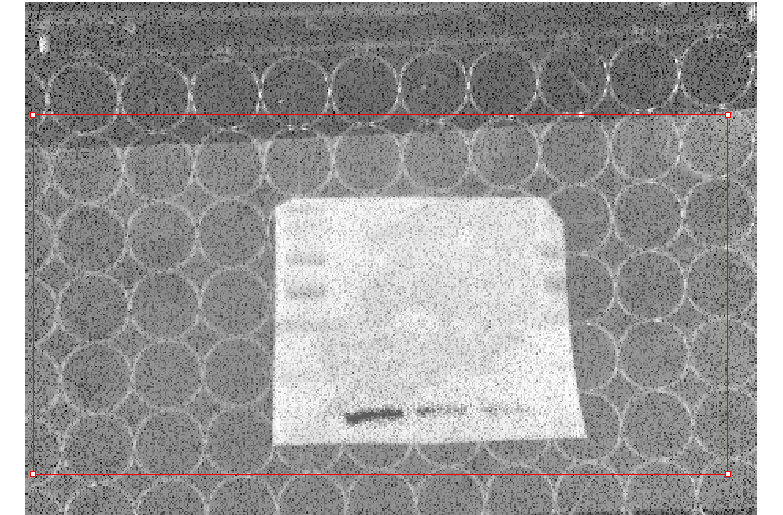
 PCNA（29 kDa）


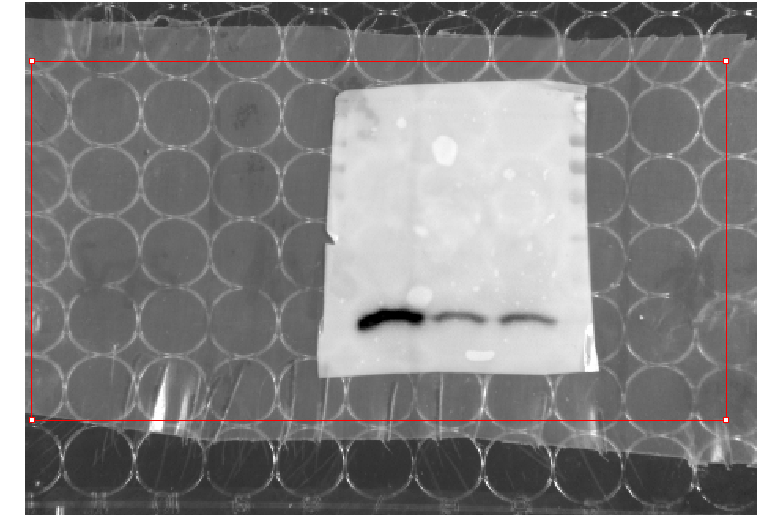
 BAP31（28kDa）
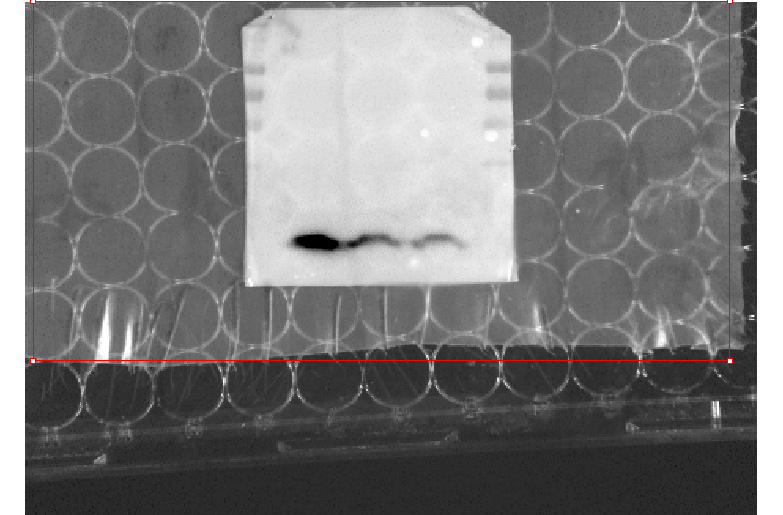
 BAP31（28kDa）


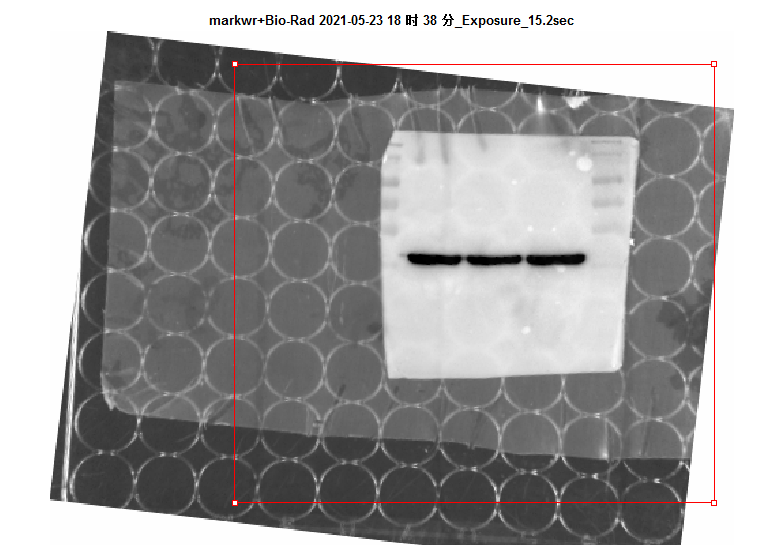
 β-actin（42KDa）
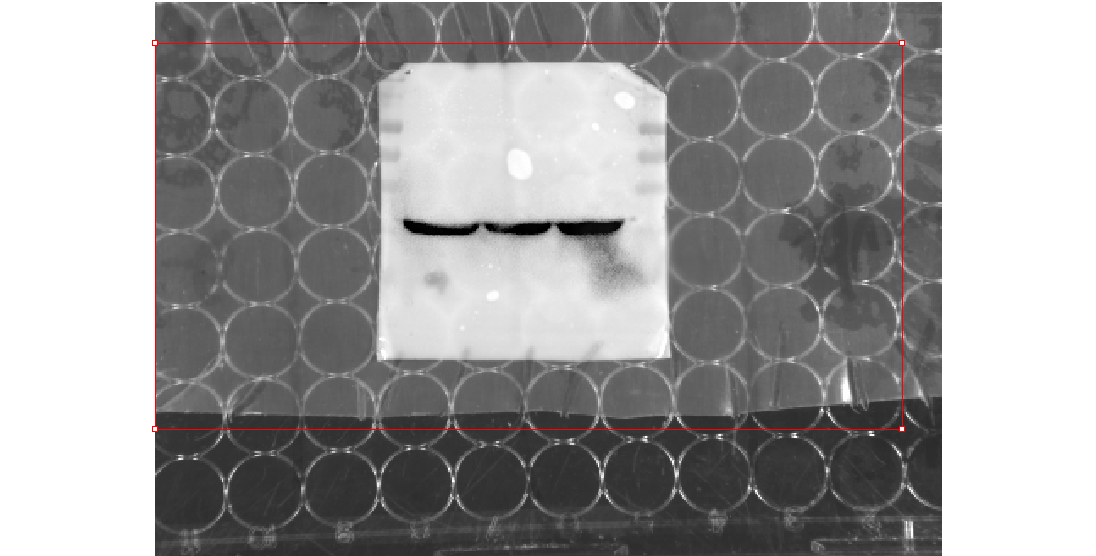
 β-actin（42KDa）

Fig5B


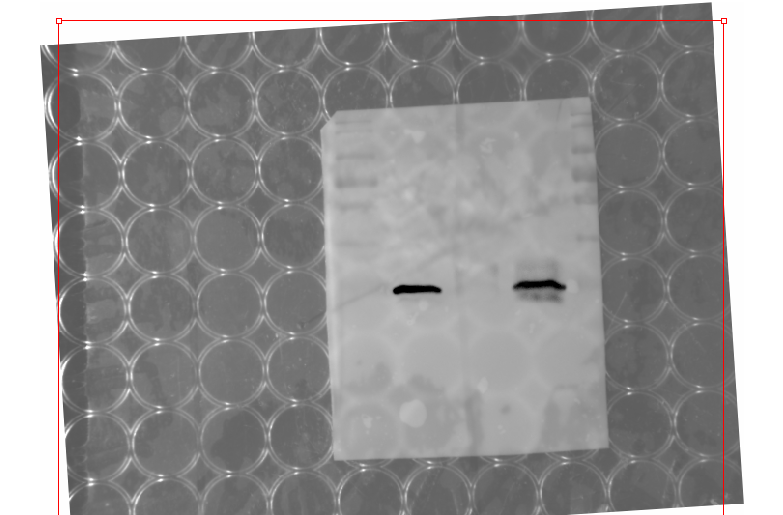
 VDAC1(31 kDa)
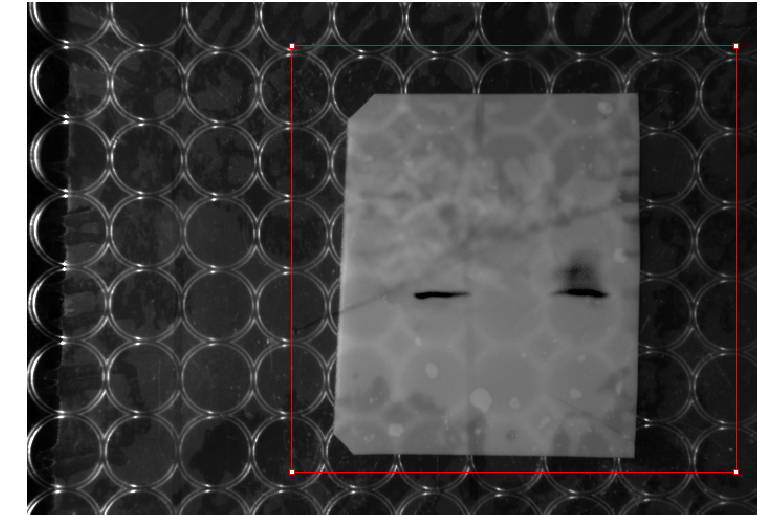
 VDAC1(31 kDa)


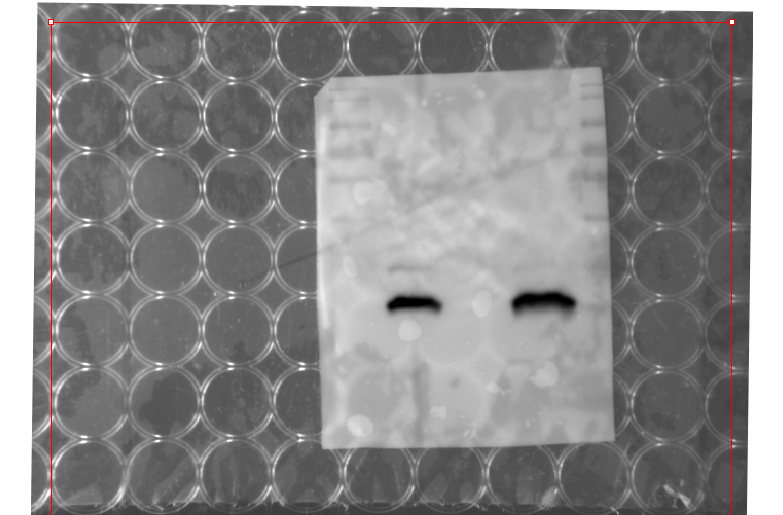
 BAP31（28kDa）
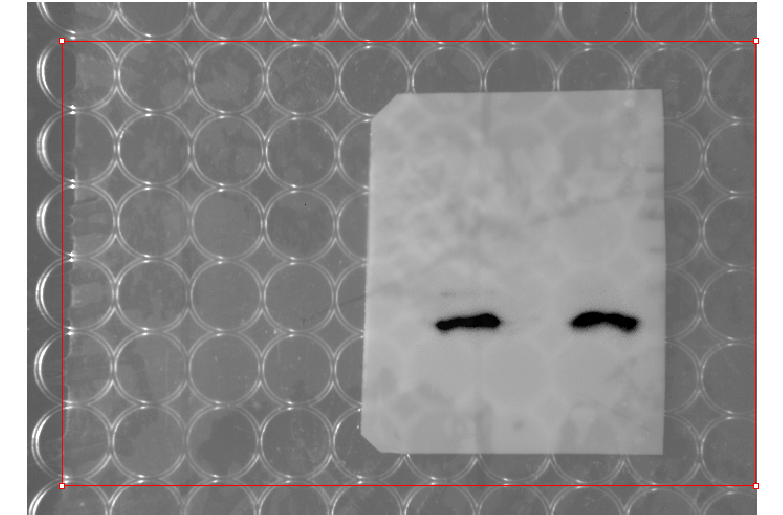
 BAP31（28kDa）


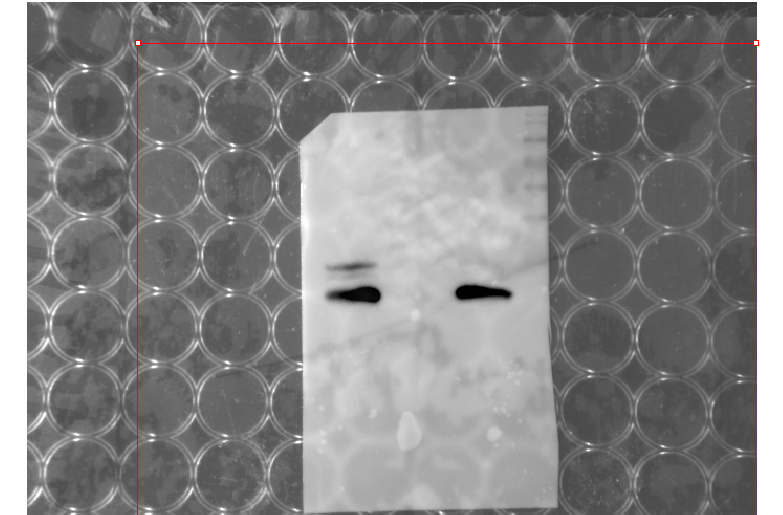
 BAP31（28kDa）
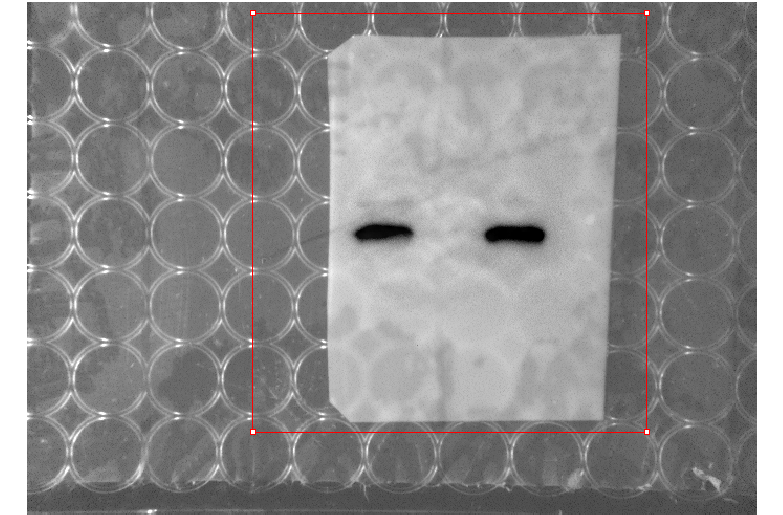
 BAP31（28kDa）


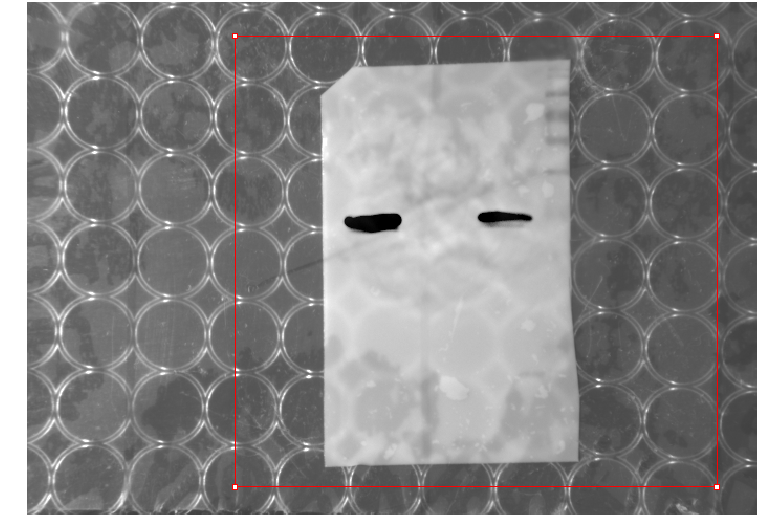
 VDAC1(31 kDa)
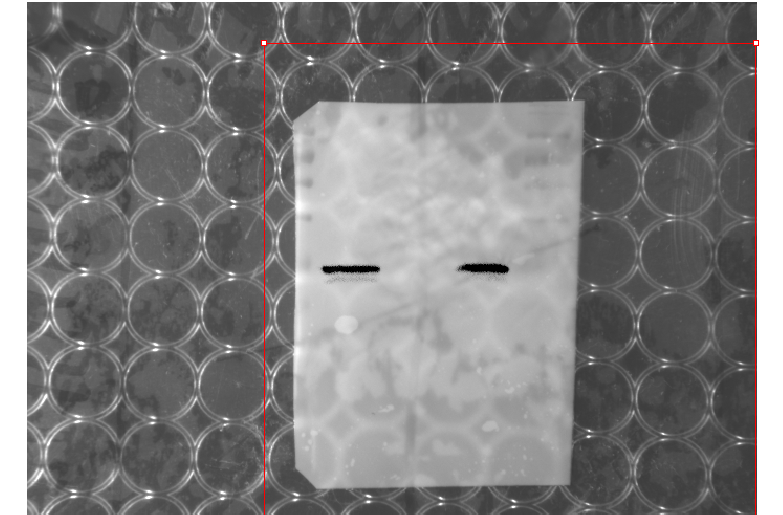
VDAC1(31 kDa)

Fig 5E


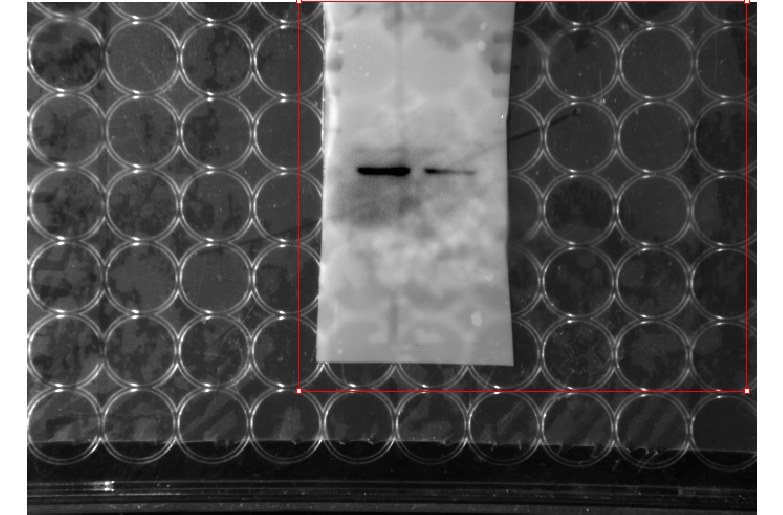
 VDAC1(31 kDa)
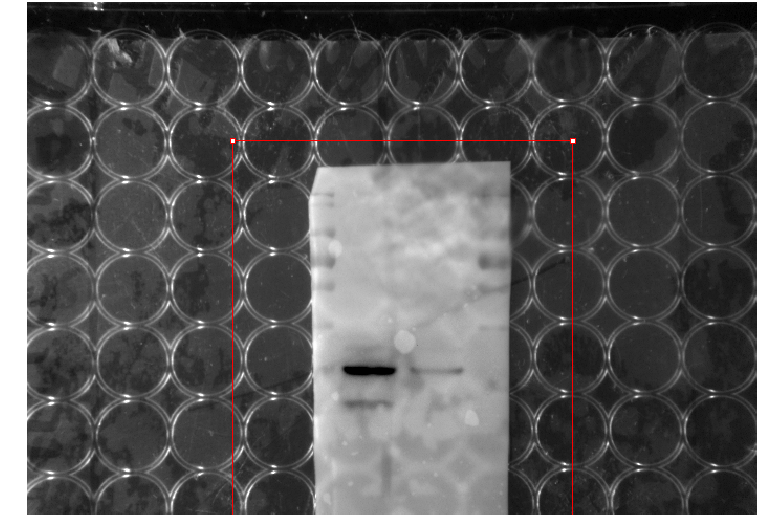
 VDAC1(31 kDa)


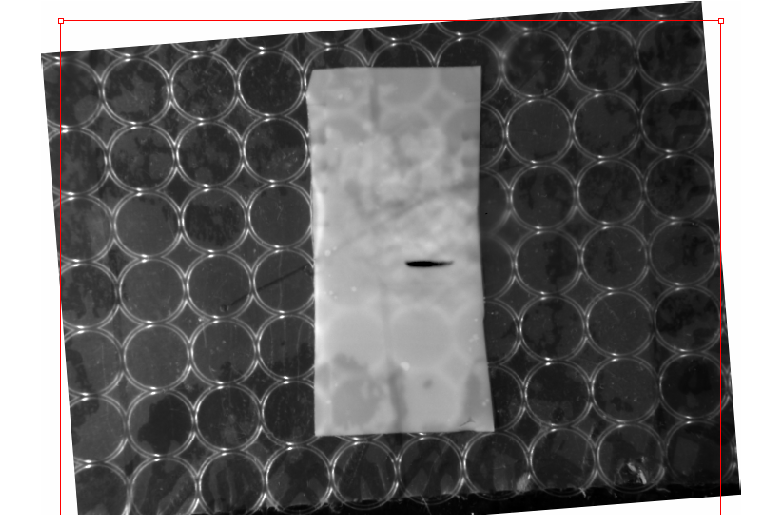
 Flag-BAP31（28kDa）
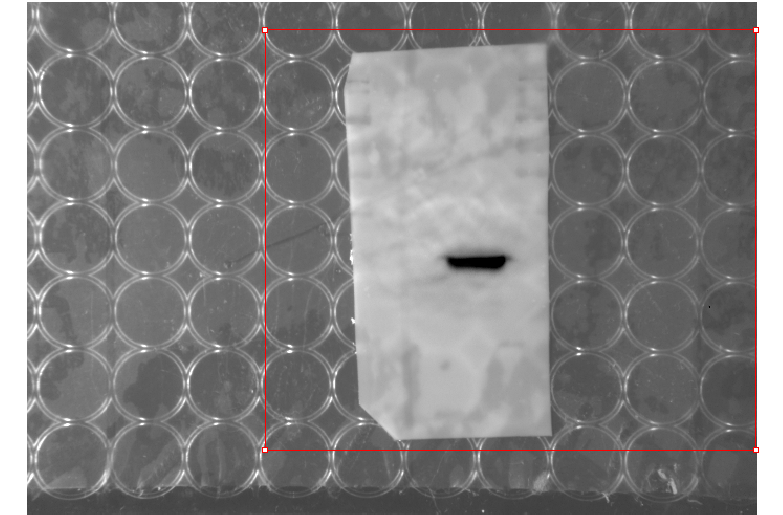
 Flag-BAP31（28kDa）


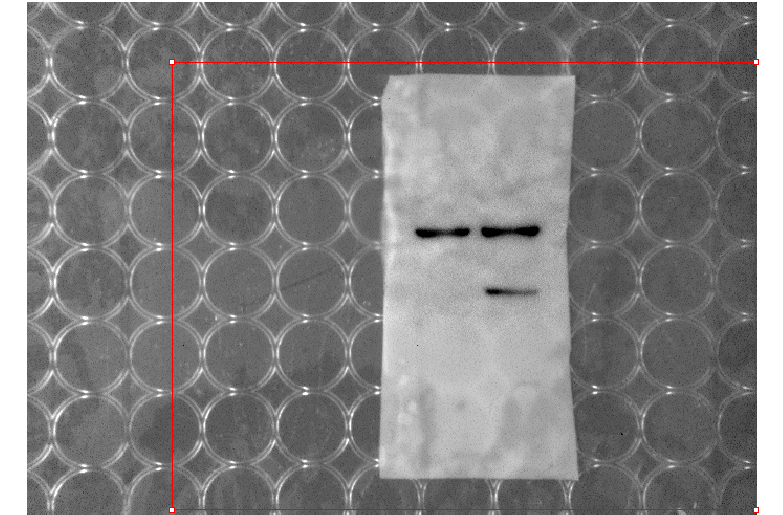
 β-actin（42KDa）
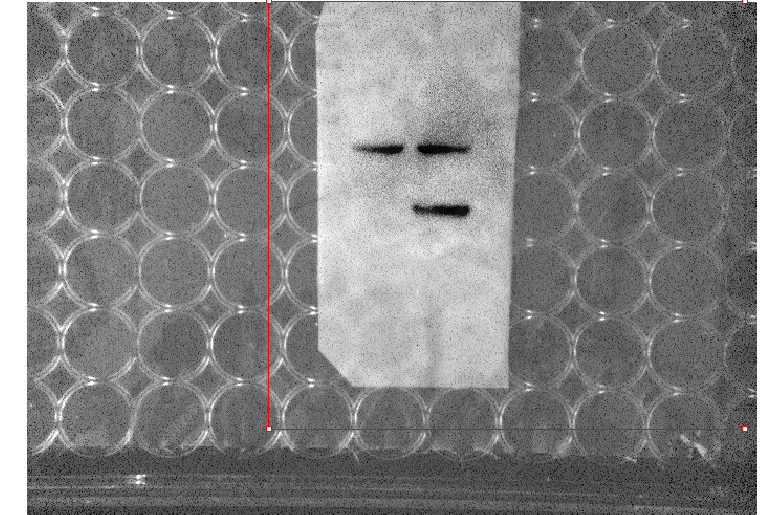
 β-actin（42KDa）


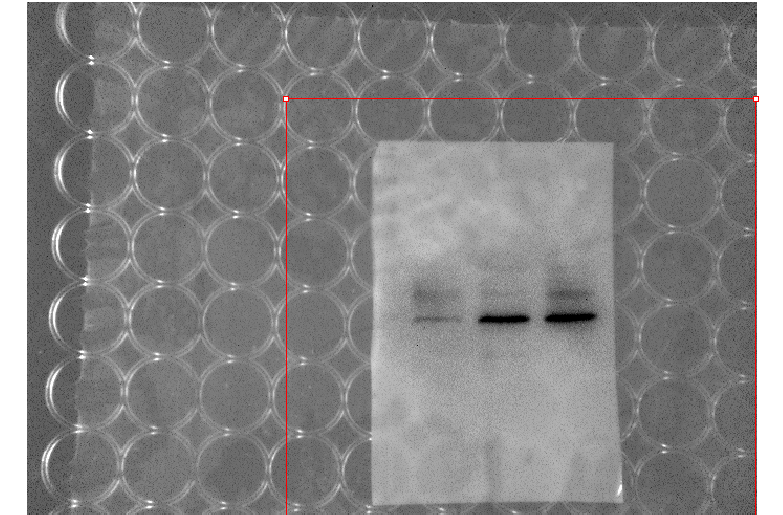
 VDAC1(31 kDa)
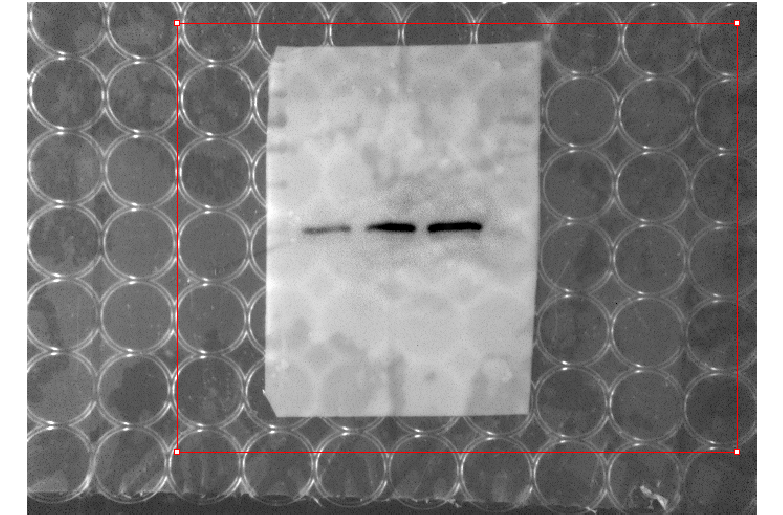
 VDAC1(31 kDa)


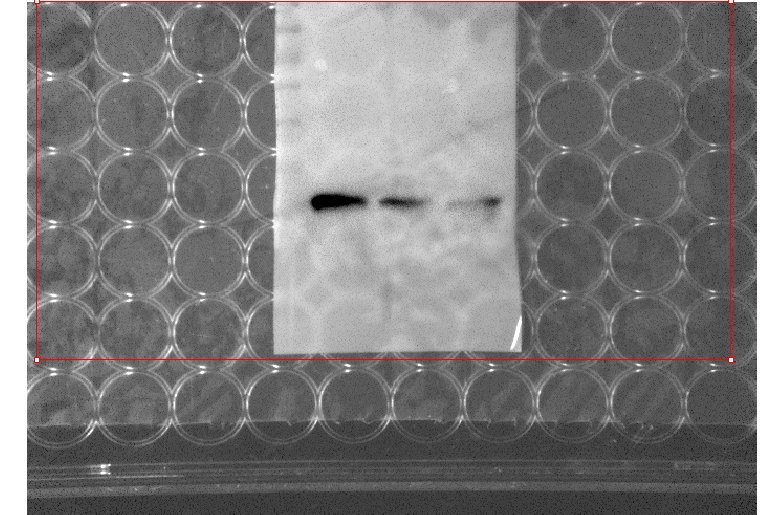
 BAP31（28kDa）
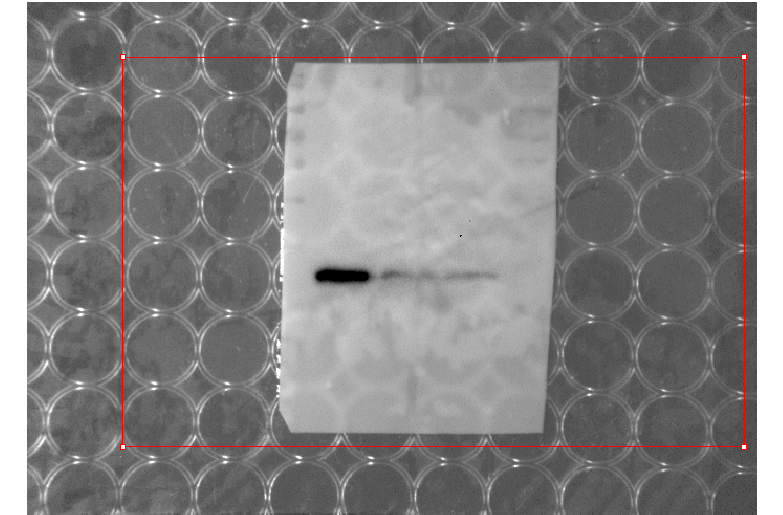
 BAP31（28kDa）


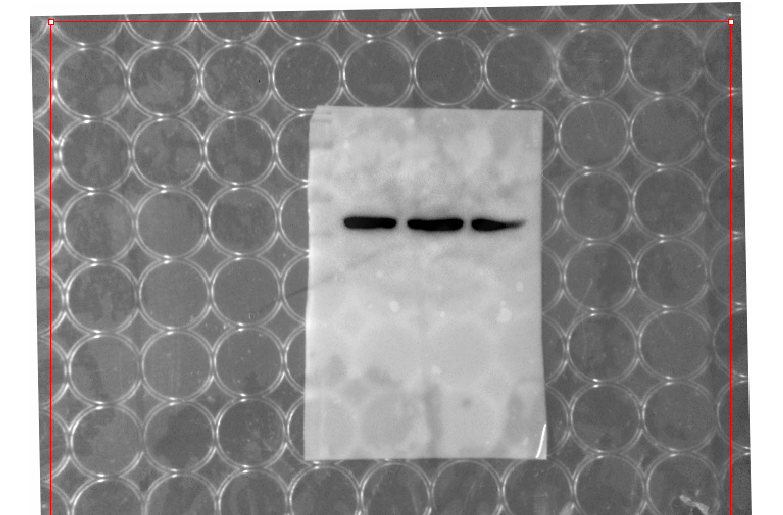
 β-actin（42KDa）
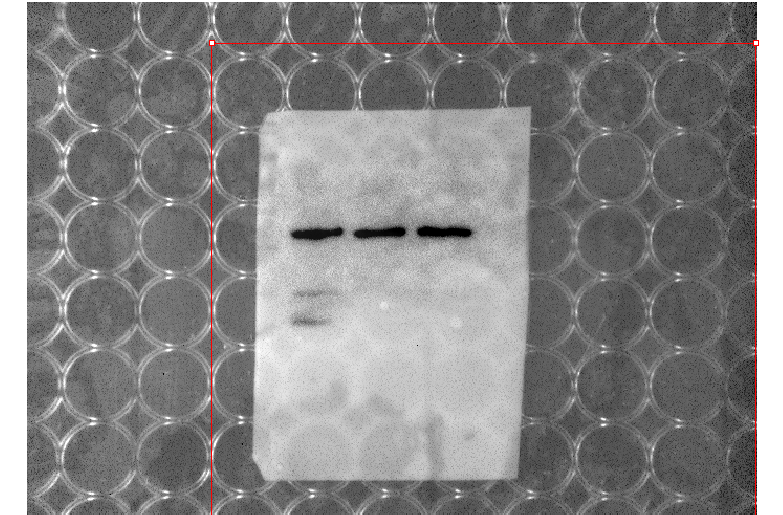
 β-actin（42KDa）

Fig 5F


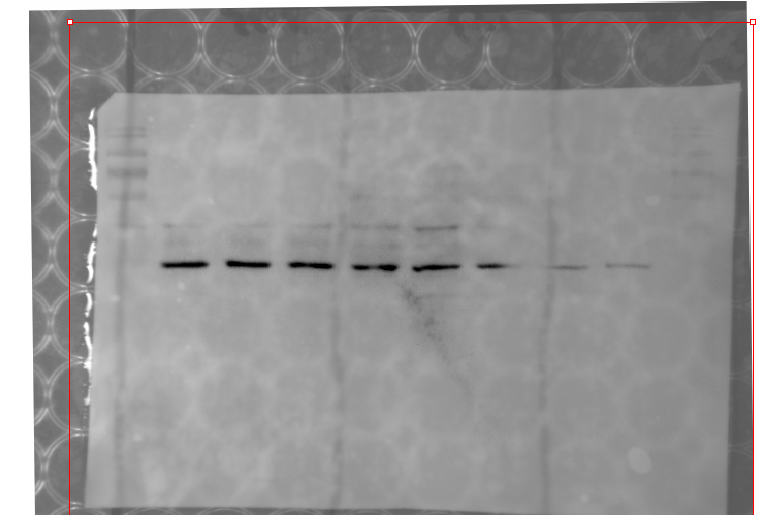
 VDAC1(31 kDa)
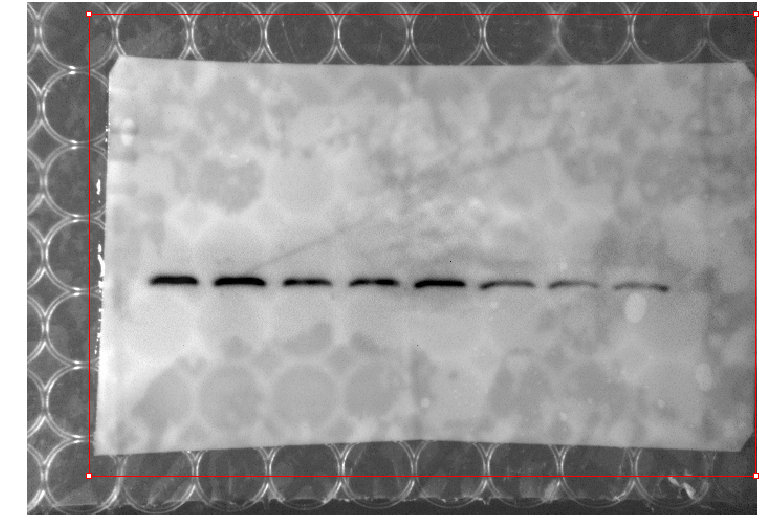
 VDAC1(31 kDa)


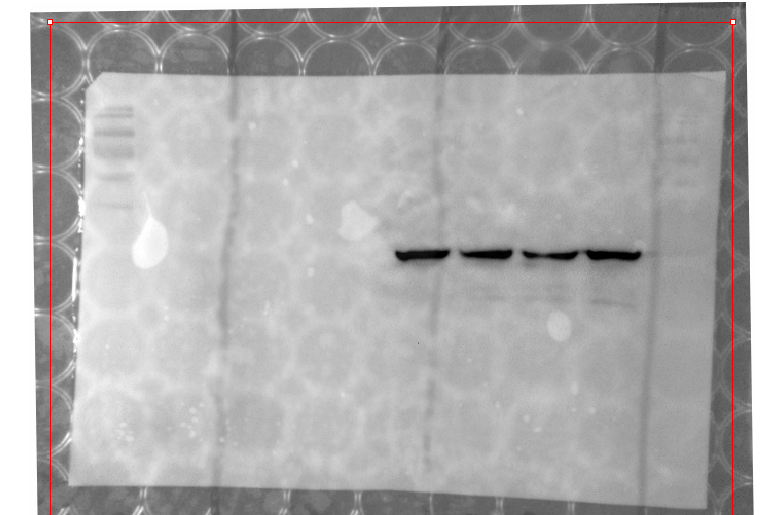
 Flag-BAP31（28kDa）
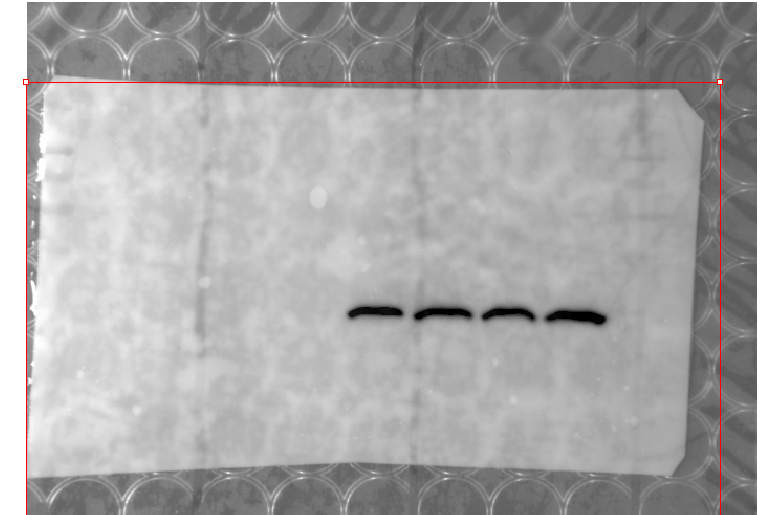
 Flag-BAP3（28kDa）


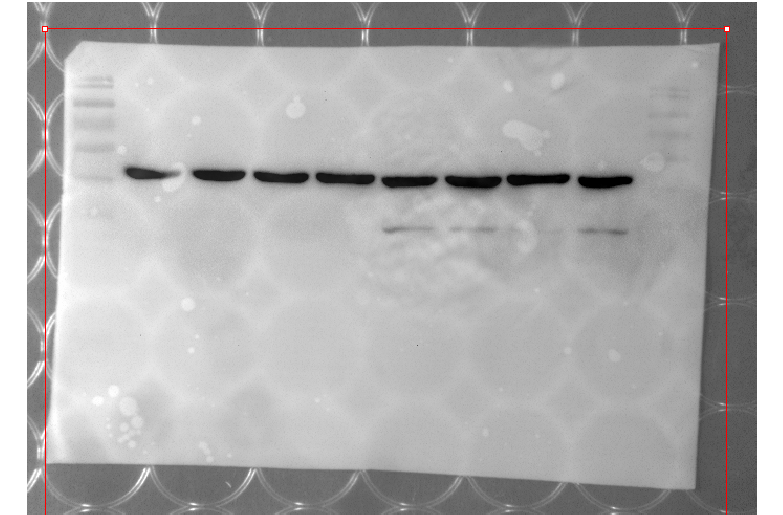
 β-actin（42KDa）
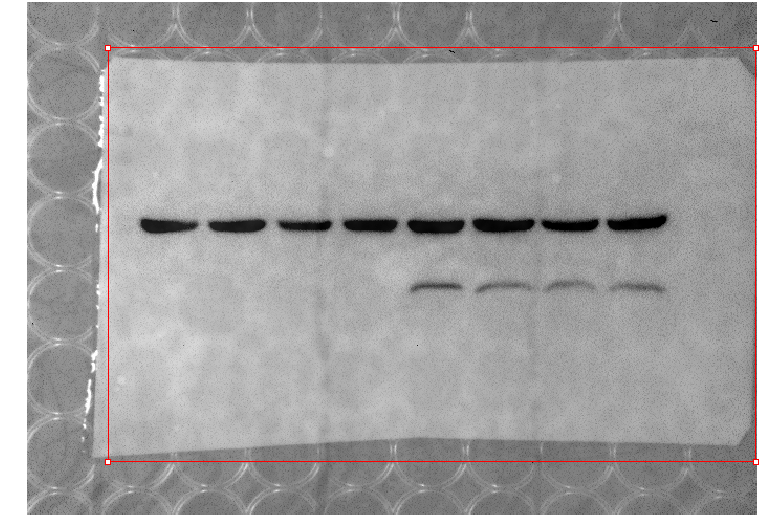
 β-actin（42KDa）

Fig 5G


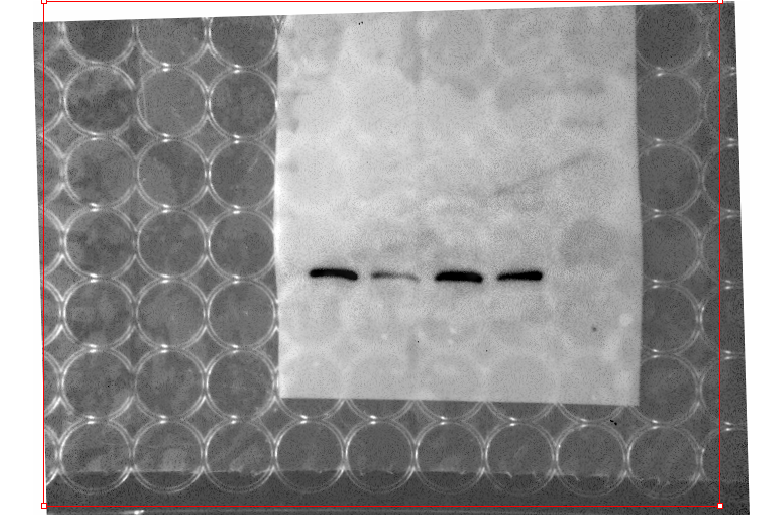
 VDAC1(31 kDa)
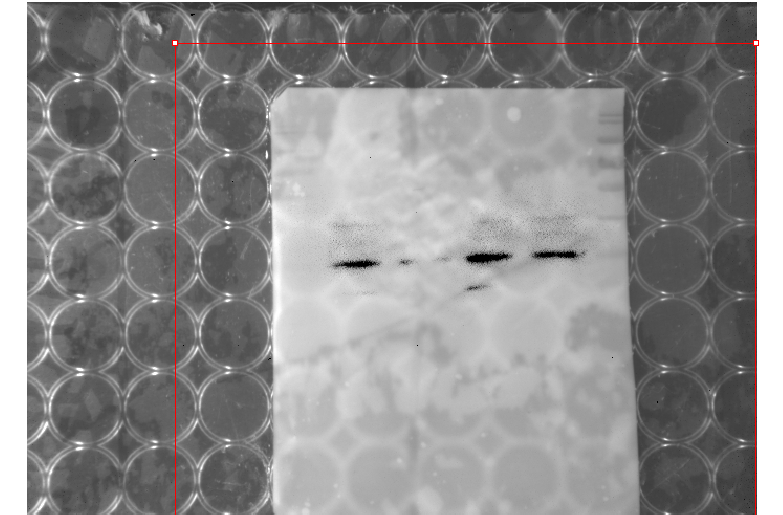
 VDAC1(31 kDa)


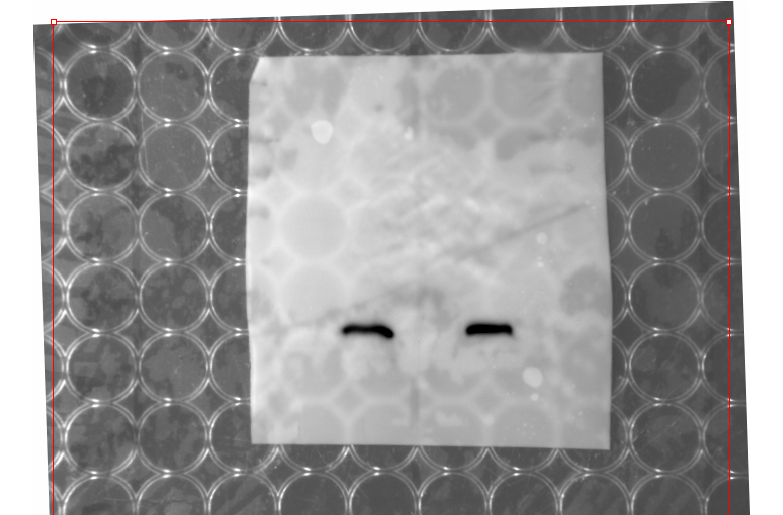
 Flag-BAP31（28kDa）
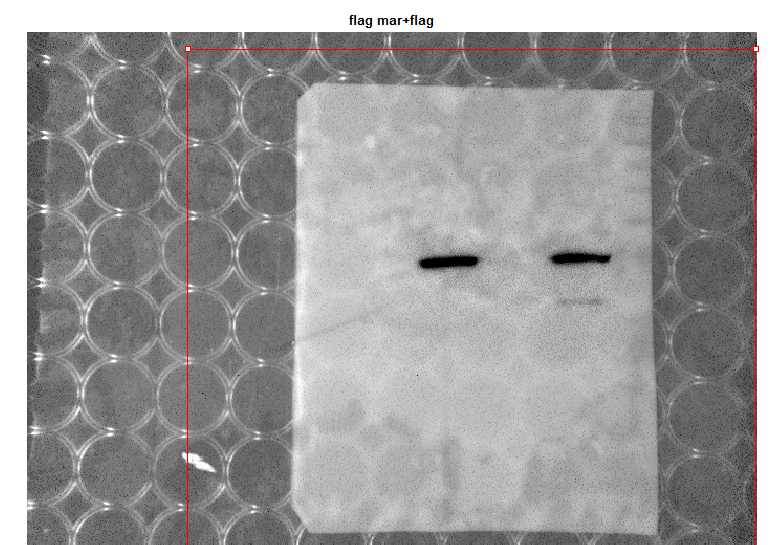
 Flag-BAP31（28kDa）


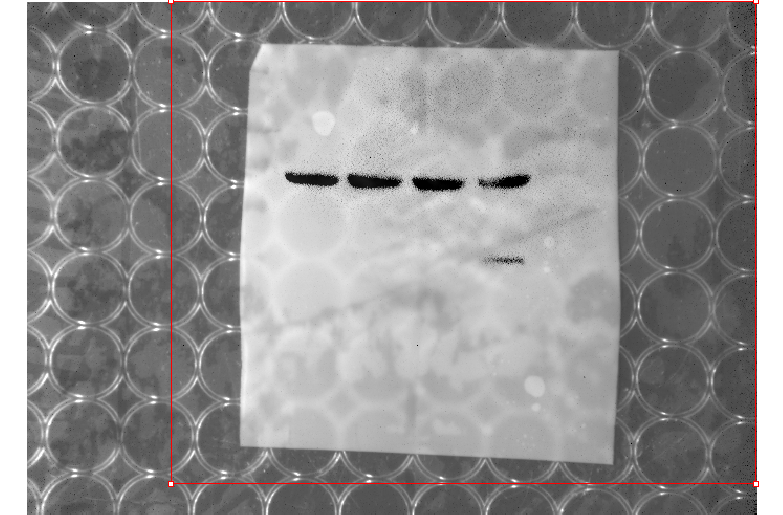
β-actin（42KDa）
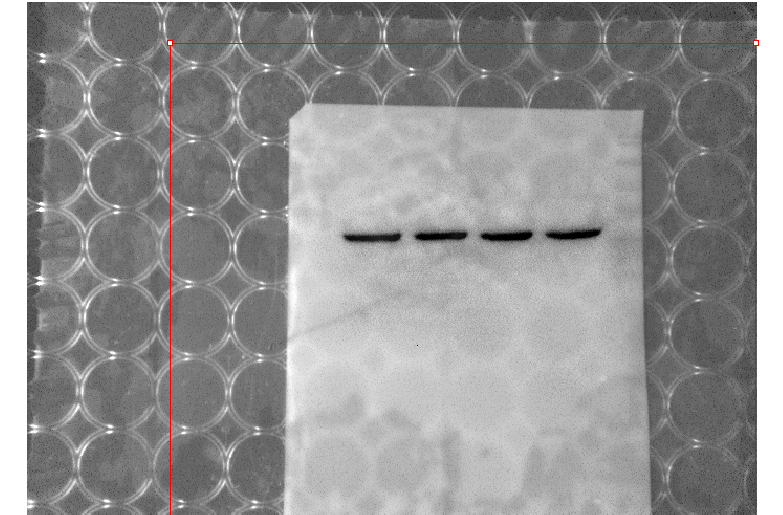
 β-actin（42KDa）

Fig 5H


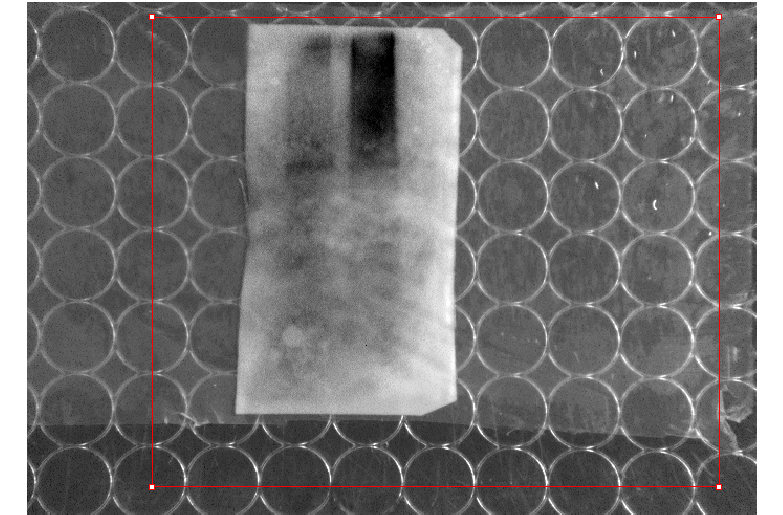
 Ub
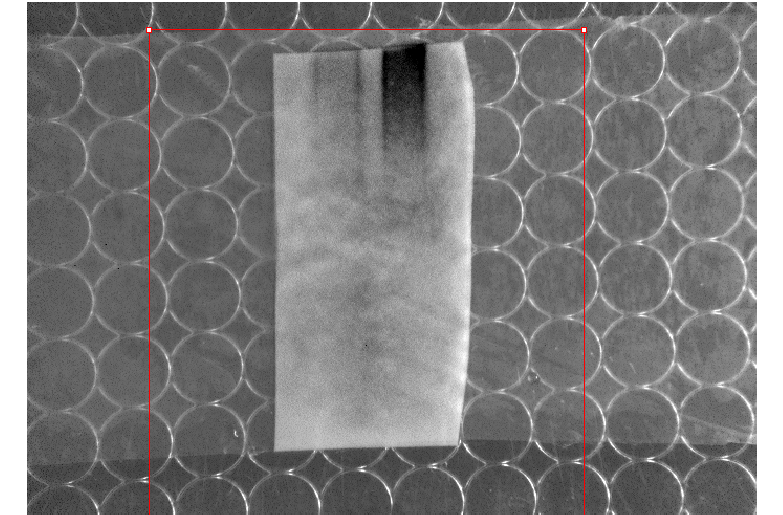
 Ub


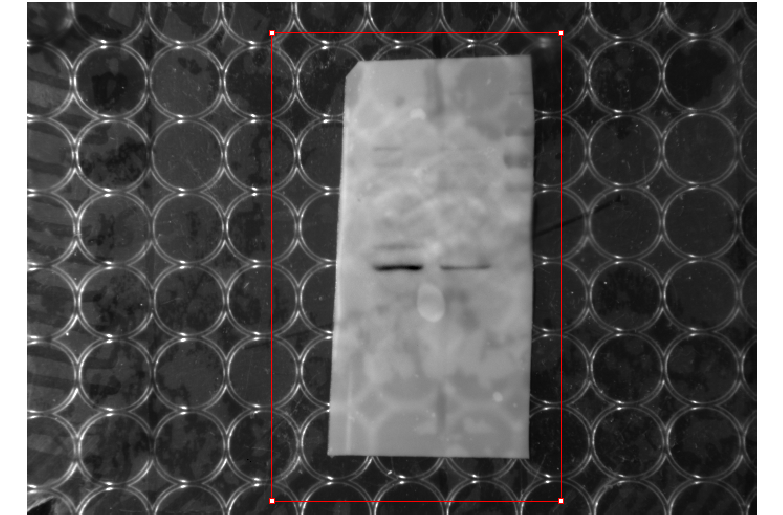
 VDAC1(31 kDa)
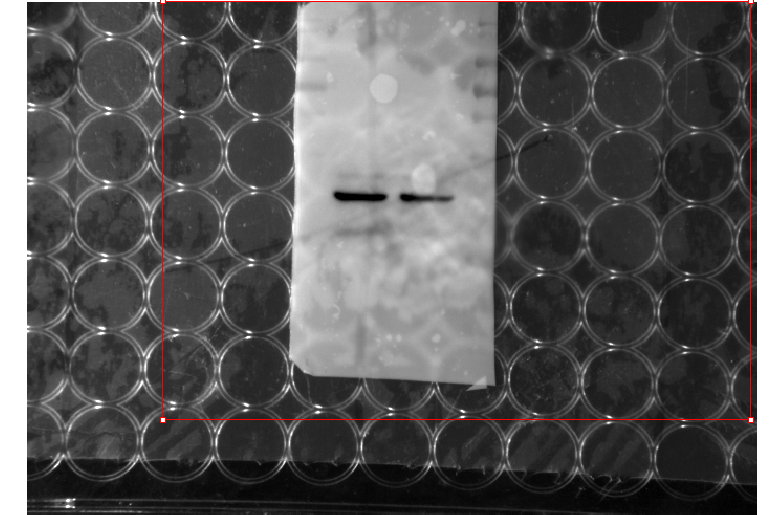
 VDAC1(31 kDa)


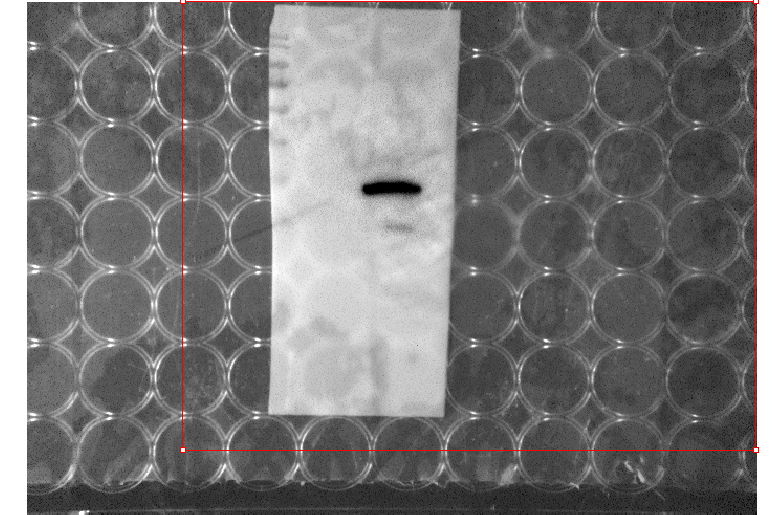
 Flag-BAP31（28kDa）
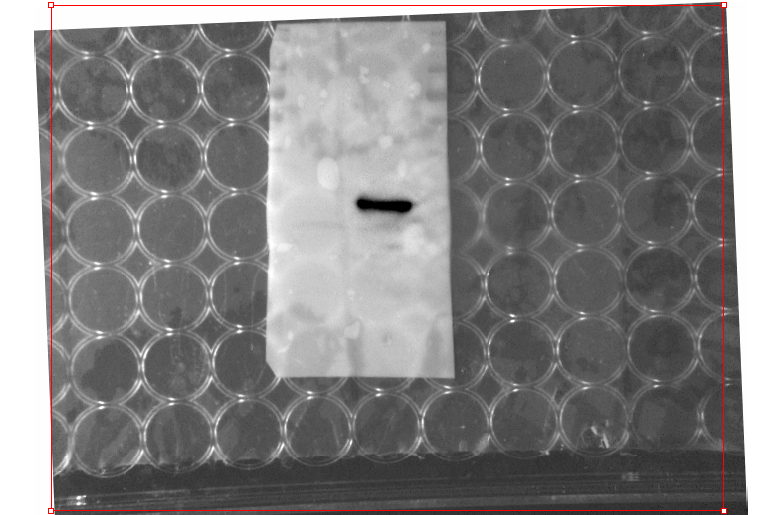
 Flag-BAP31（28kDa）


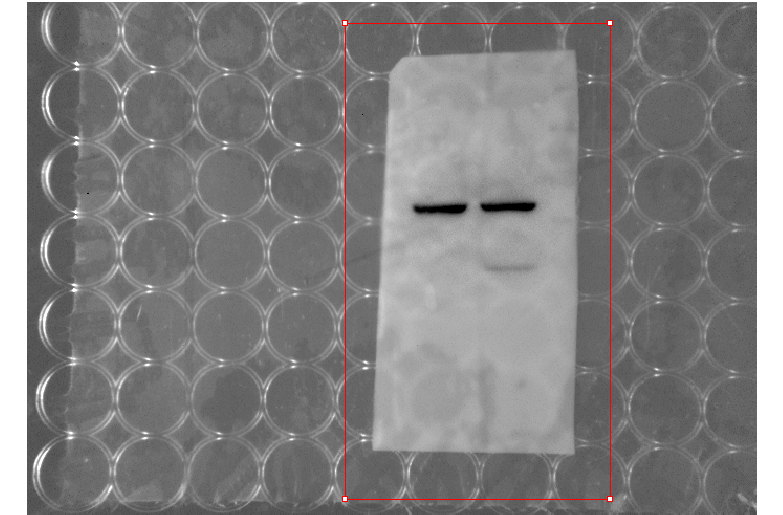
β-actin（42KDa）
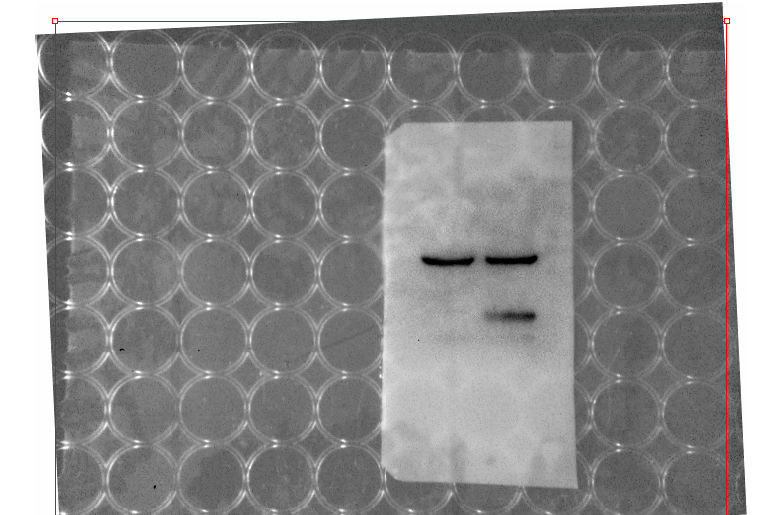
β-actin（42KDa）

Fig 5I


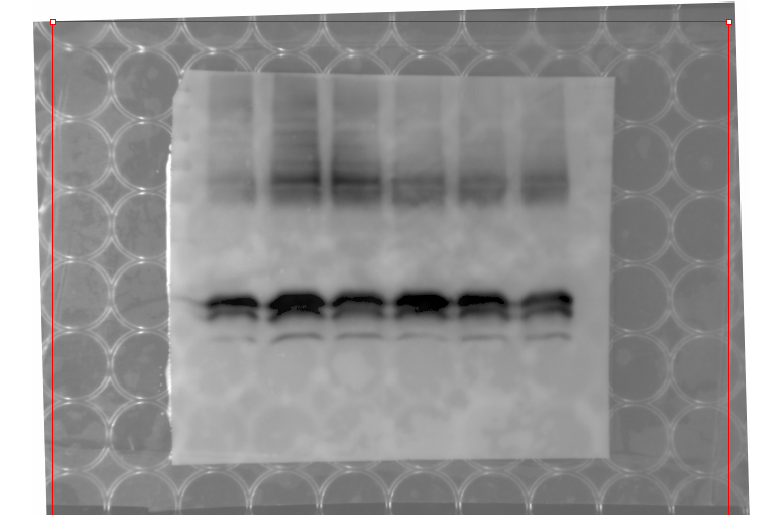

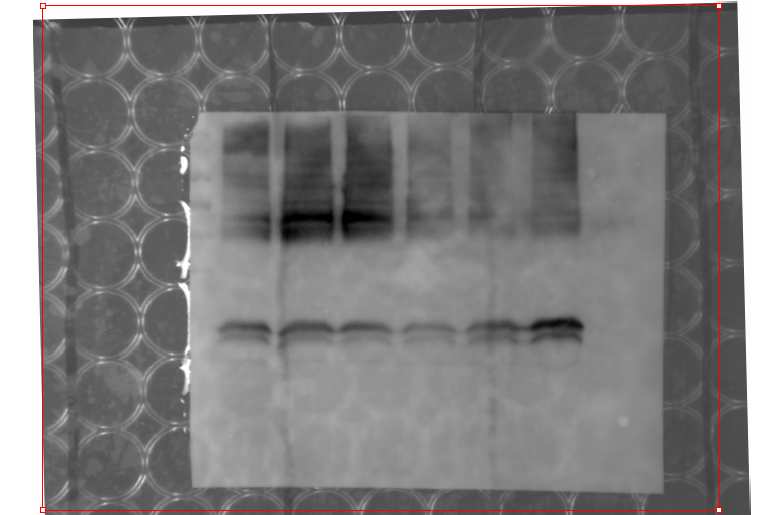


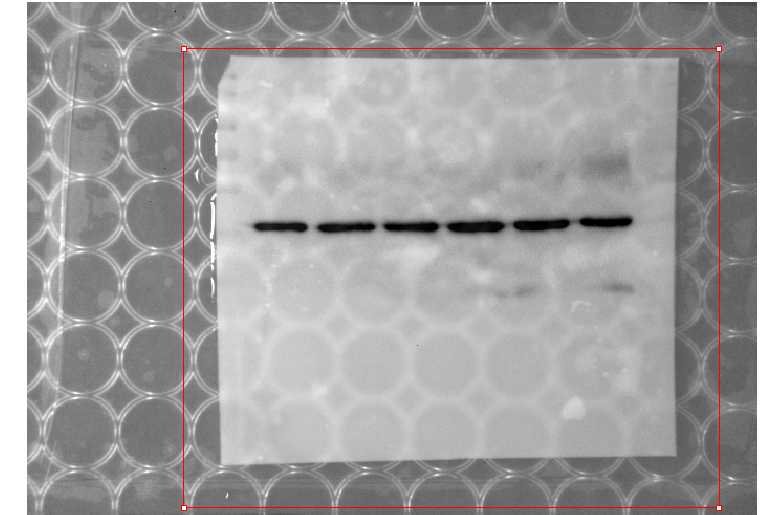
β-actin（42KDa）
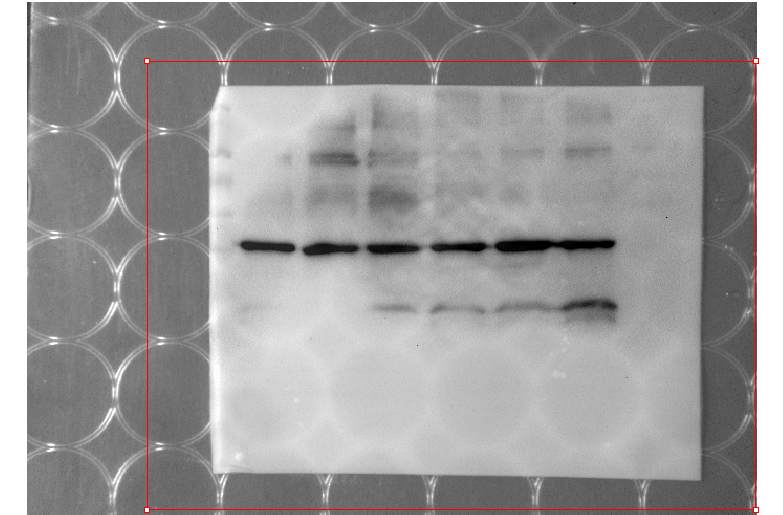
β-actin（42KDa）

Fig 5L


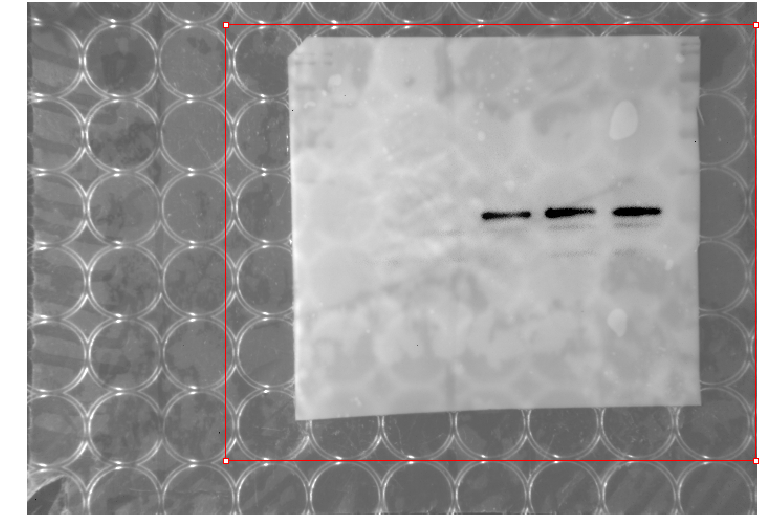
 HA-VDAC1(31 kDa)
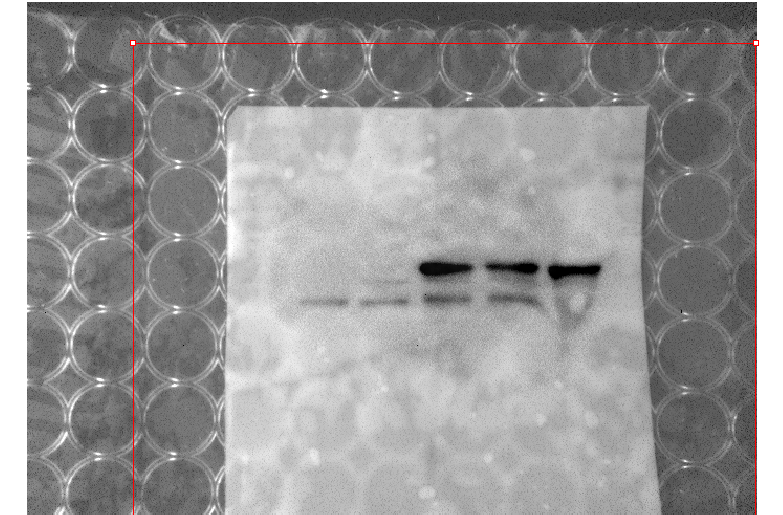
 HA-VDAC1(31 kDa)


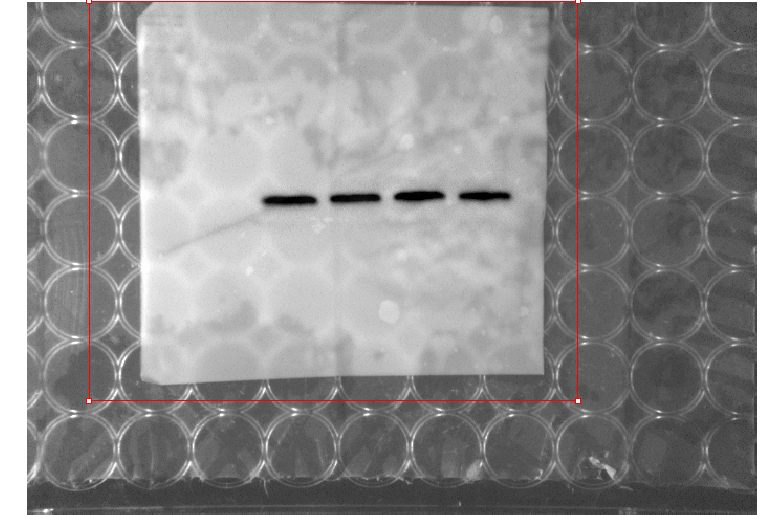
 Flag-BAP31（28kDa）
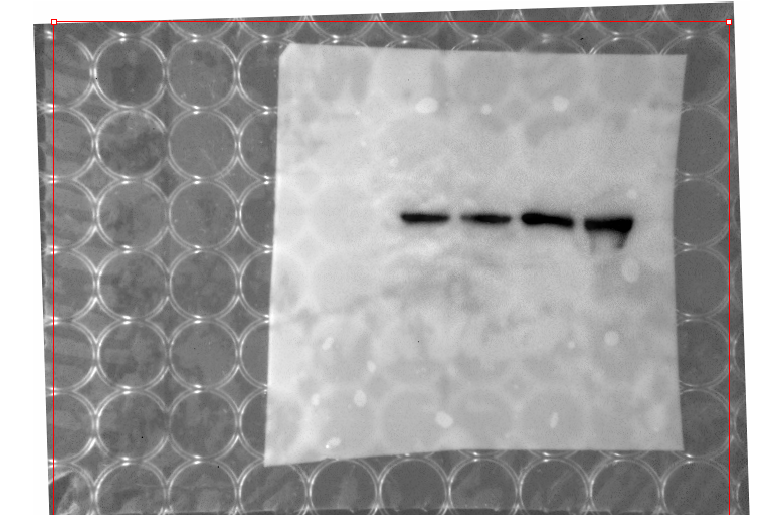
 Flag-BAP31（28kDa）


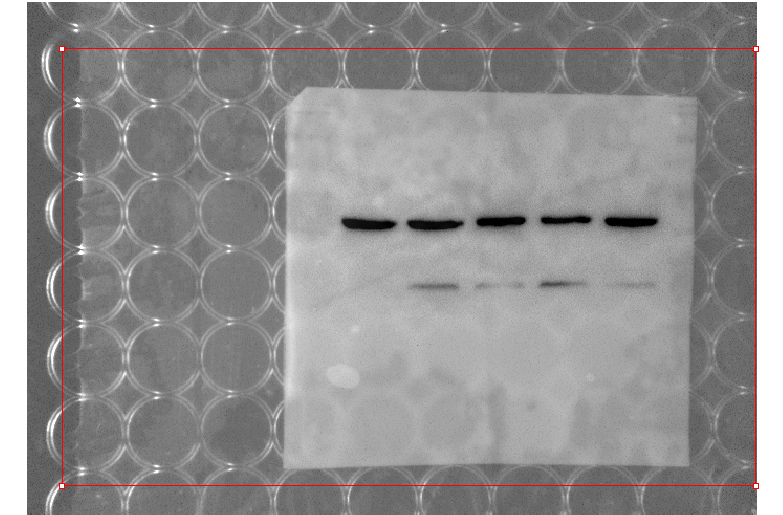
β-actin（42KDa）
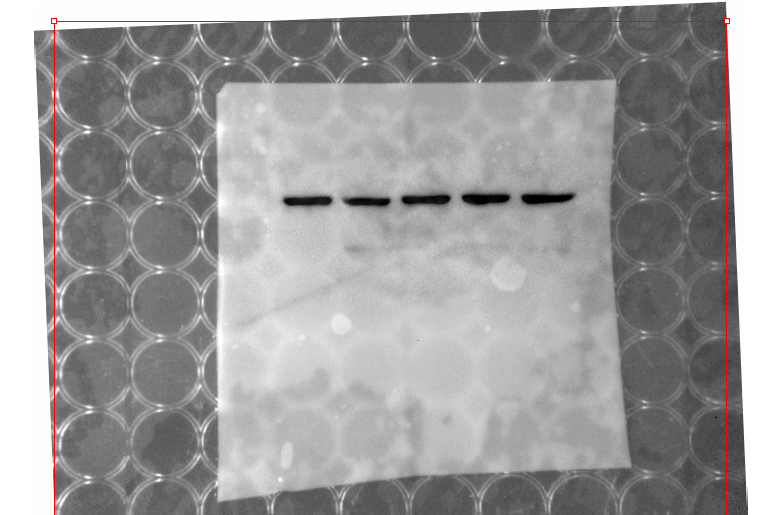
 β-actin（42KDa）

Fig 6B


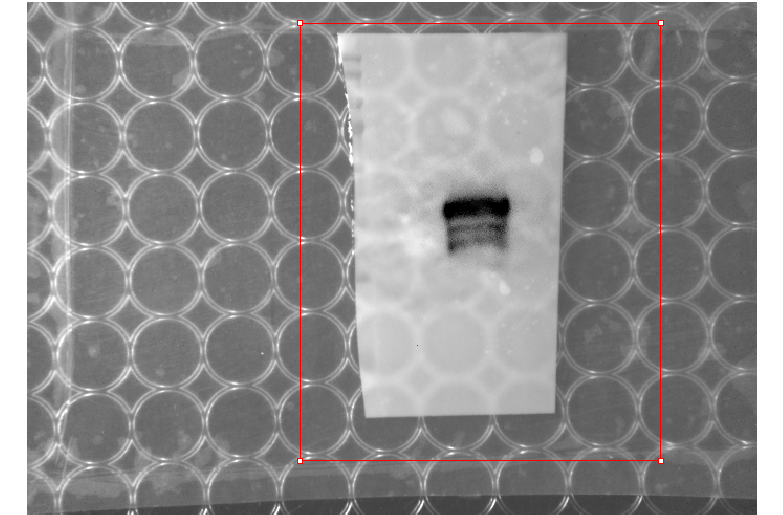
 HNF4A(53 kDa)
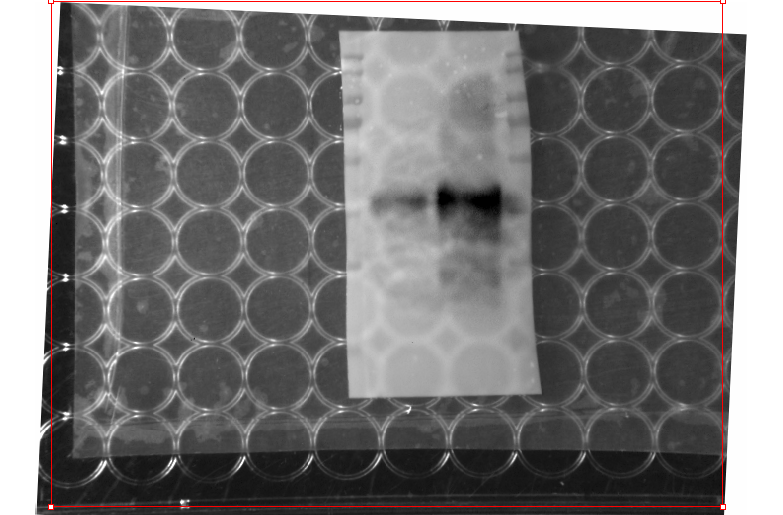
HNF4A(53 kDa)


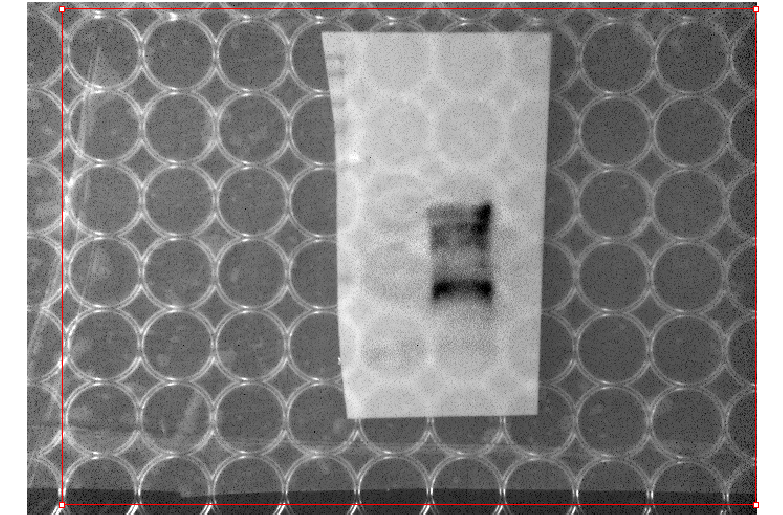
 BAP31（28kDa）
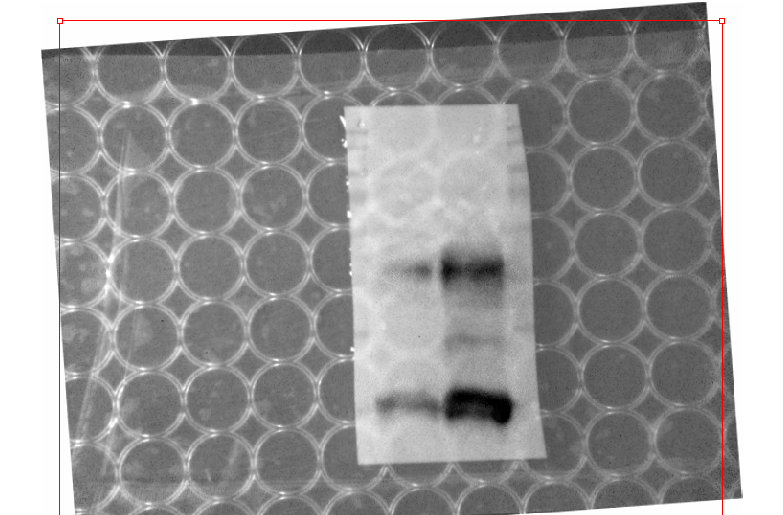
BAP31（28kDa）


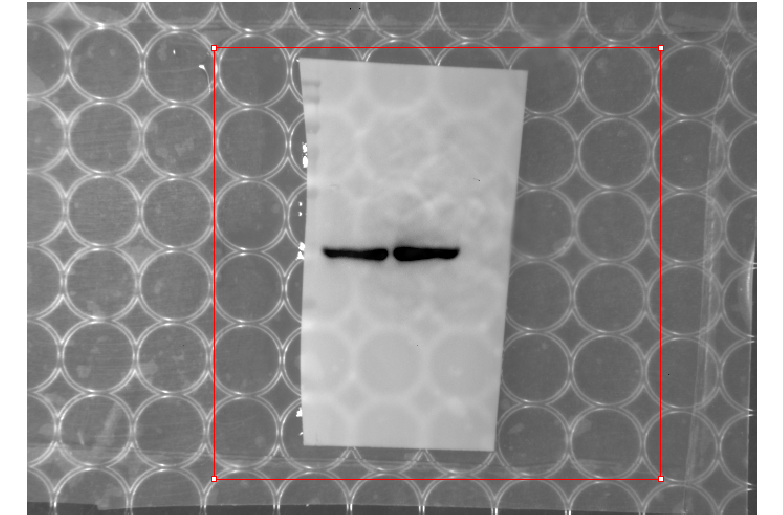
β-actin（42KDa）
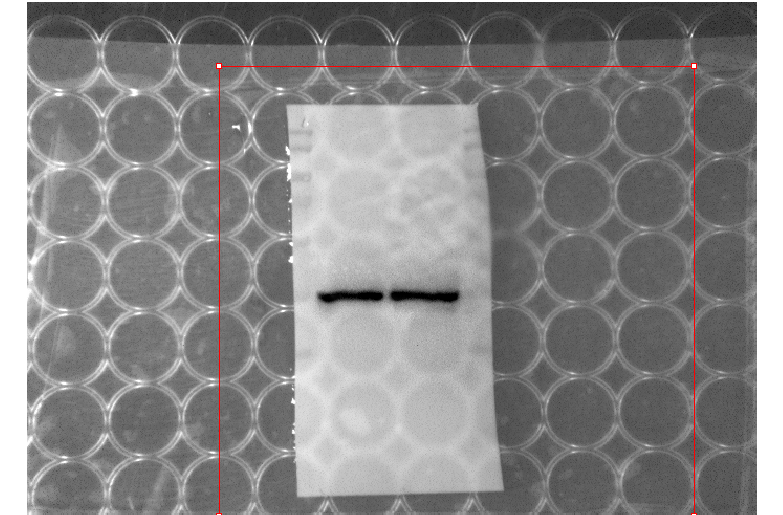


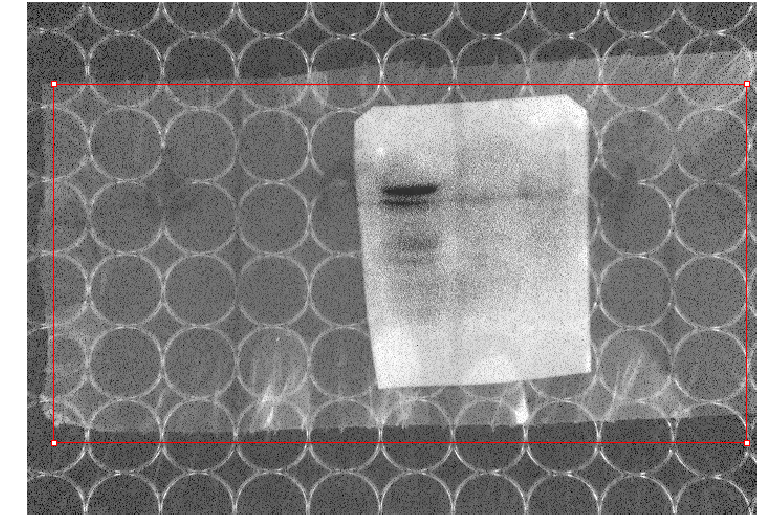
HNF4A(53 kDa)
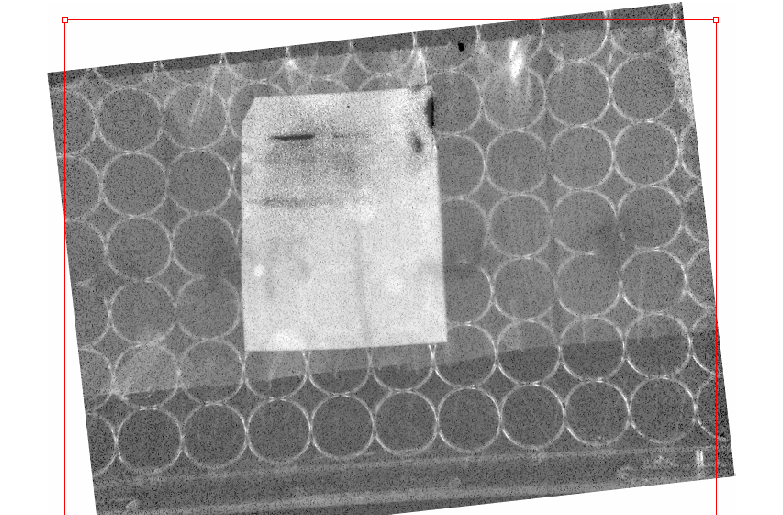
 HNF4A(53 kDa)


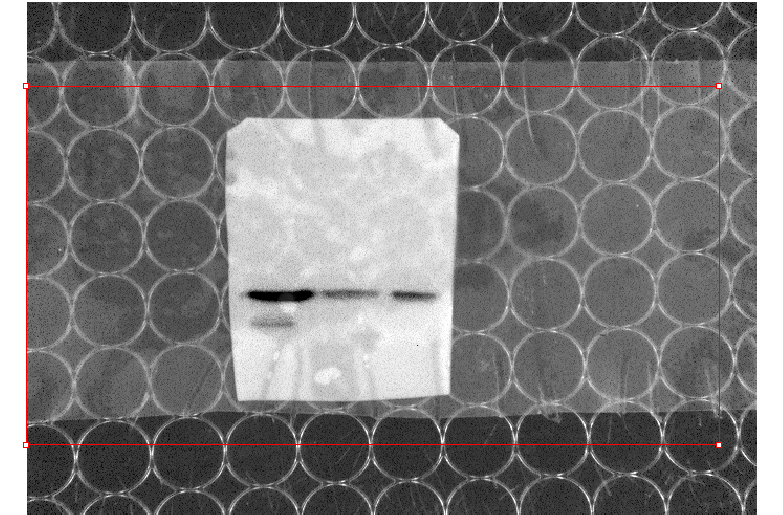
 BAP31（28kDa）
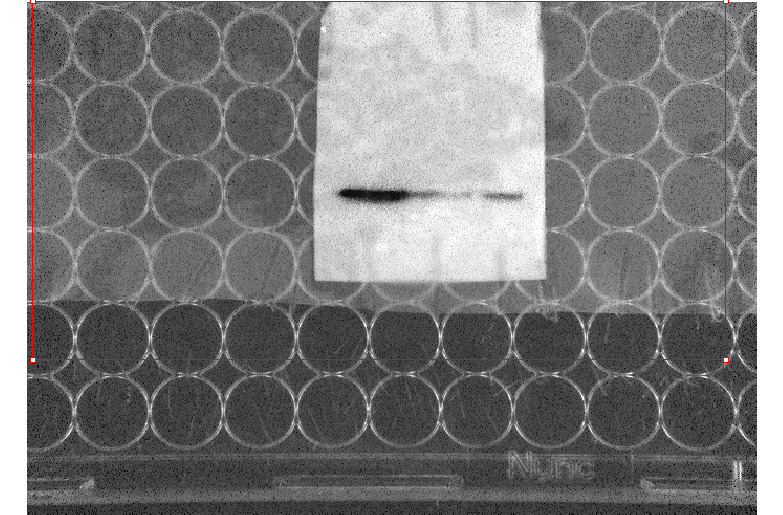
 BAP31（28kDa）


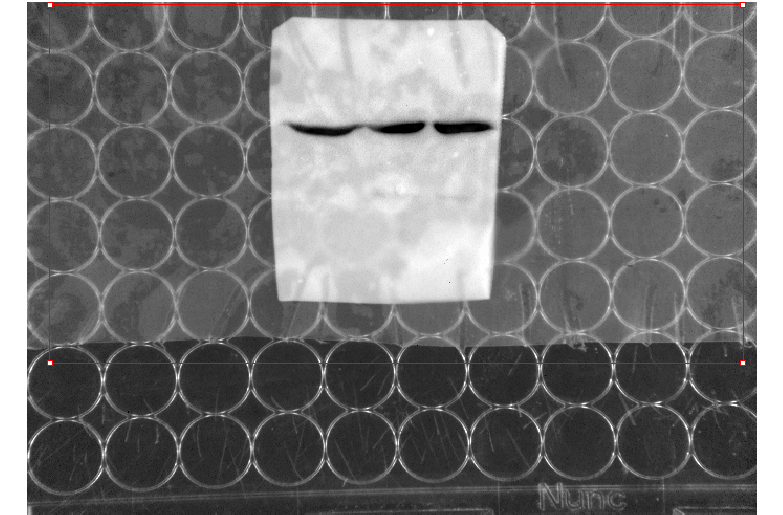
β-actin（42KDa）
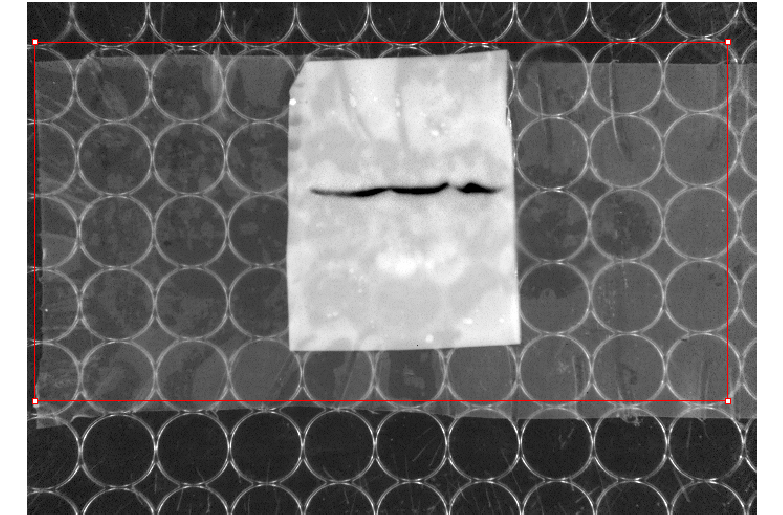
 β-actin（42KDa）

Fig 6C


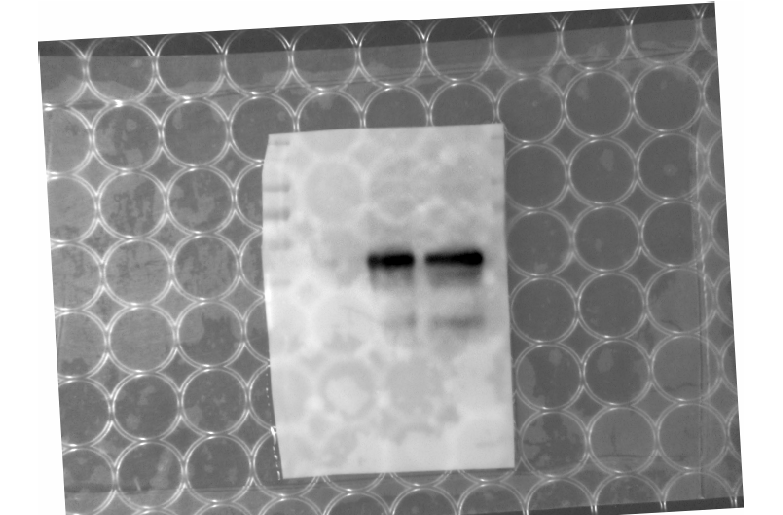
 HNF4A(53 kDa)
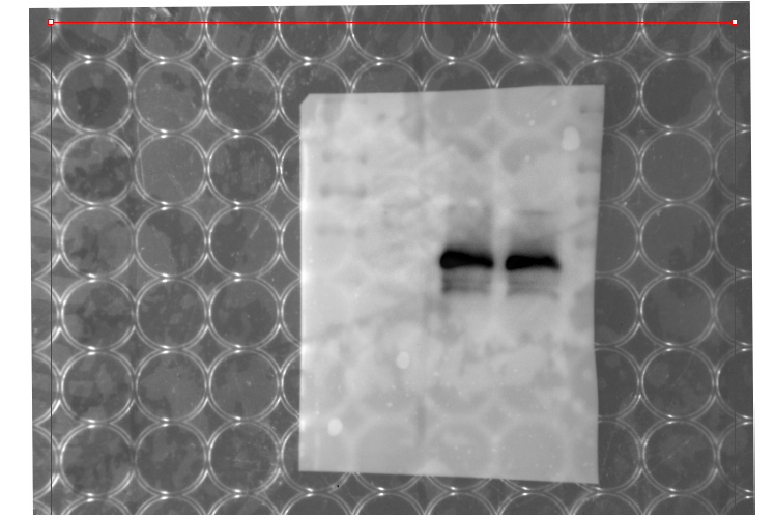
 HNF4A(53 kDa)


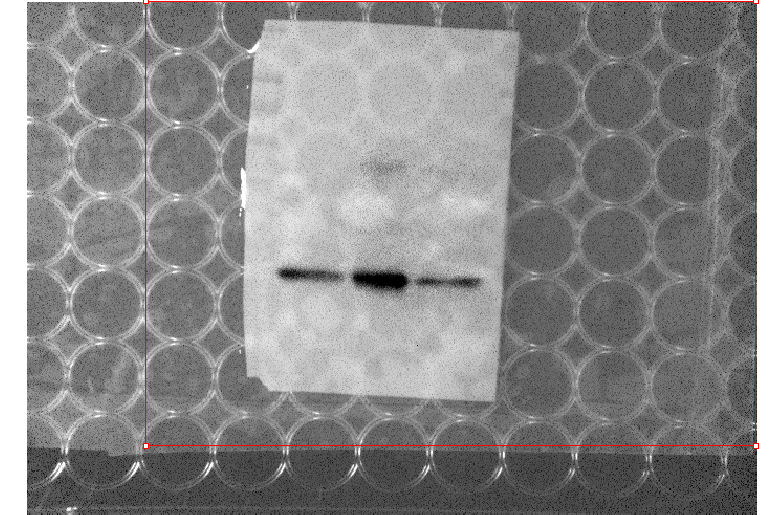
 BAP31（28kDa）
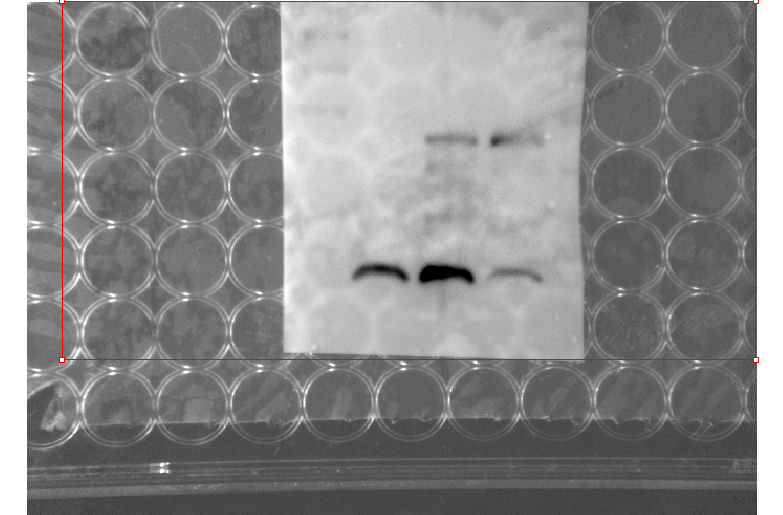
 BAP31（28kDa）


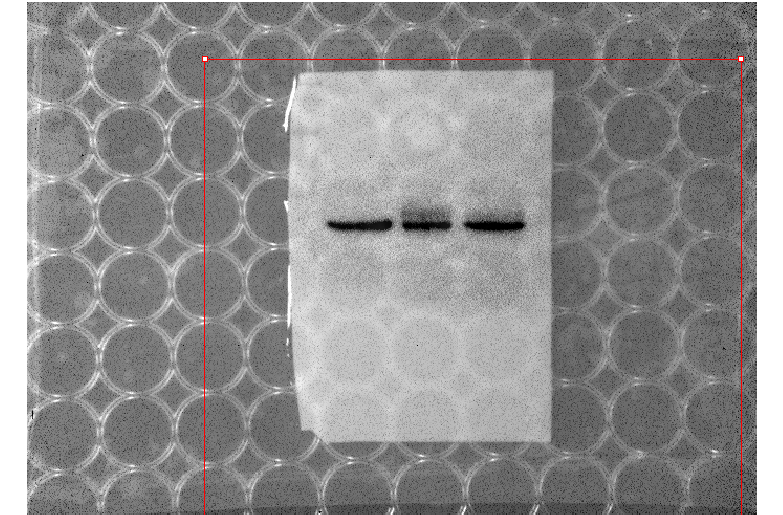
β-actin（42KDa）
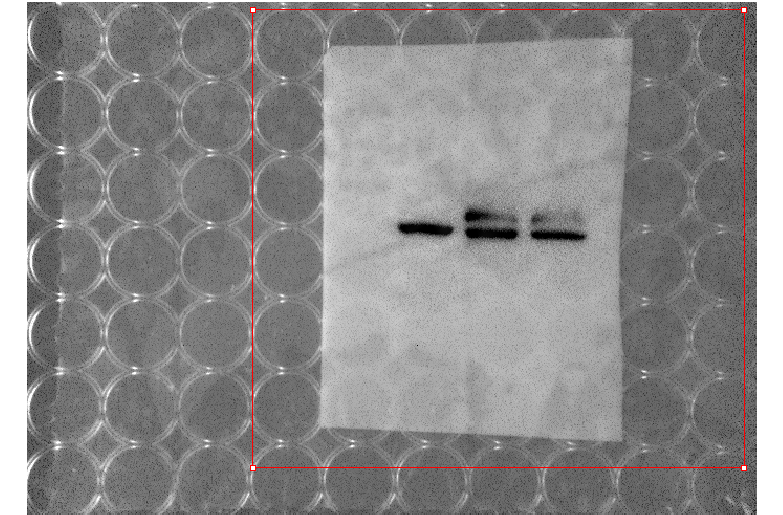
β-actin（42KDa）

Fig 6O


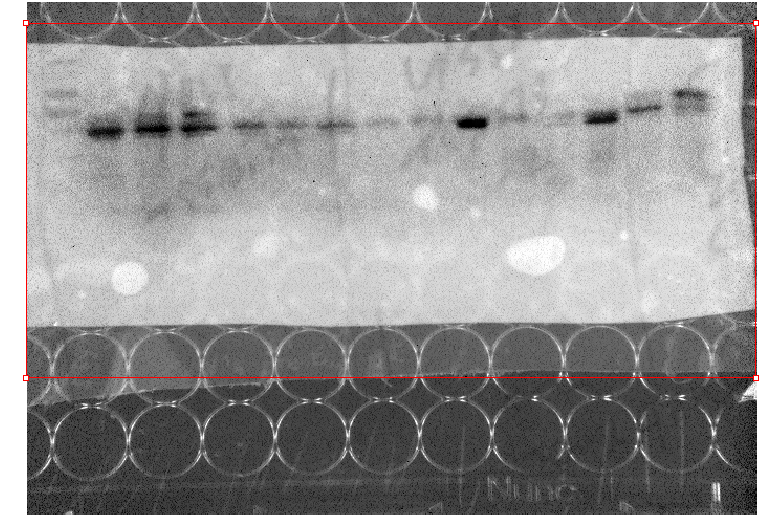
 HNF4A(53 kDa)
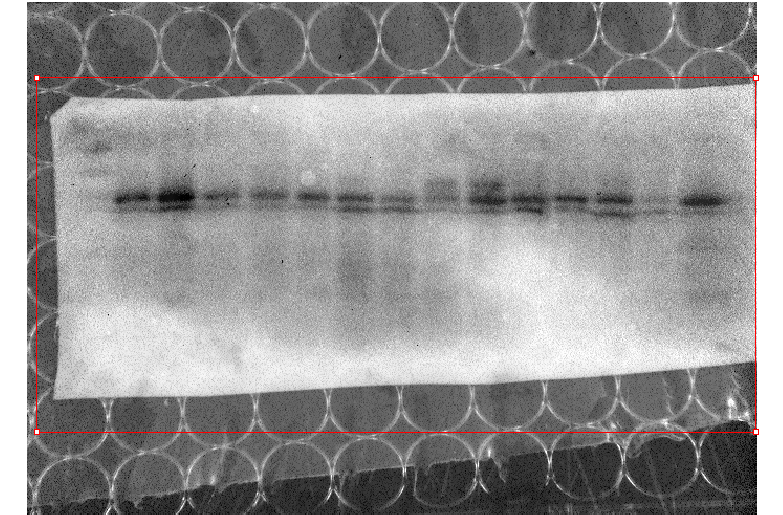
 HNF4A(53 kDa)


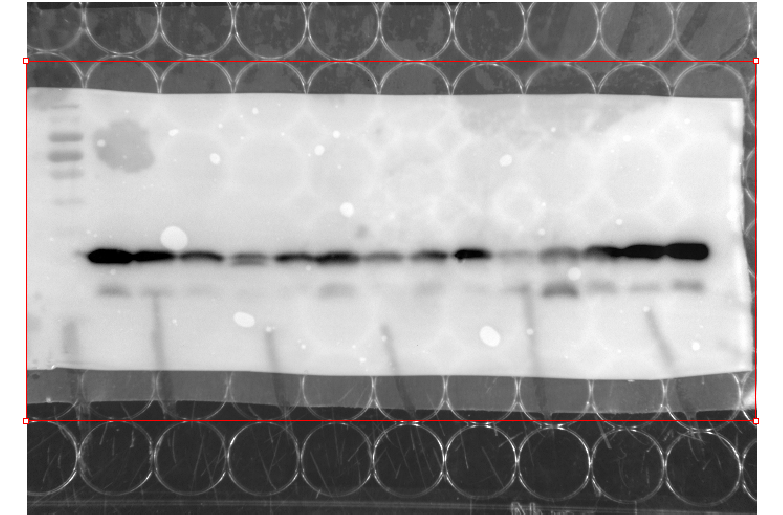
 BAP31（28kDa）
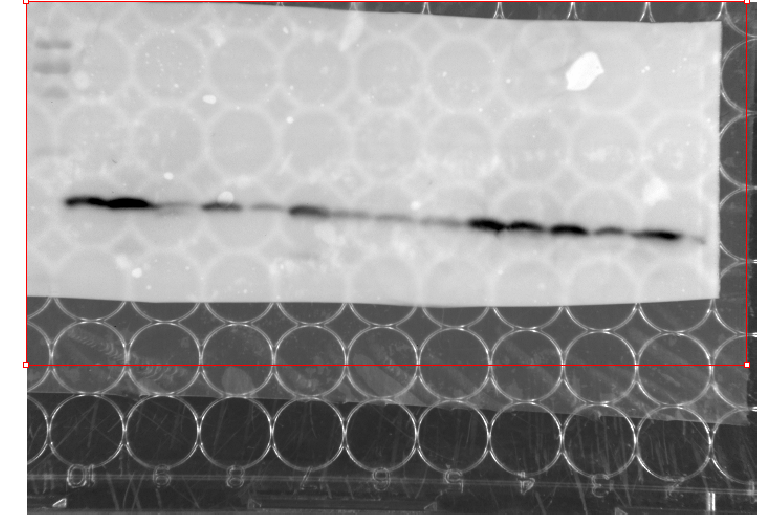
 BAP31（28kDa）

β-actin（42KDa）β-actin（42KDa）

Fig S3A

P-p38（43 kDa） P-p38（43 kDa）

p38(40 kDa) p38(40 kDa)

BAP31（28kDa） BAP31（28kDa）

β-actin（42KDa） β-actin（42KDa）

P-p38（43 kDa） P-p38（43 kDa）

p38(40 kDa) p38(40 kDa)

BAP31（28kDa） BAP31（28kDa）

β-actin（42KDa） β-actin（42KDa）

Fig S5A

VDAC2(32kDa) VDAC2(32kDa)

BAP31（28kDa） BAP31（28kDa）

VDAC3(31kDa) VDAC3(31kDa)

BAP31（28kDa） BAP31（28kDa）

Fig S5C

HA-VDAC1(31 kDa) HA-VDAC1(31 kDa)

Flag-BAP31（28kDa） Flag-BAP31（28kDa）

β-actin（42KDa） β-actin（42KDa）

Fig S6C

NRF2(110 kDa) NRF2(110 kDa)

BAP31（28kDa） BAP31（28kDa）

β-actin（42KDa）β-actin（42KDa）
